# A framework for the analysis of self-confirming policies

**DOI:** 10.1007/s11238-021-09862-9

**Published:** 2022-02-10

**Authors:** P. Battigalli, S. Cerreia-Vioglio, F. Maccheroni, M. Marinacci, T. Sargent

**Affiliations:** 1 Università Bocconi and IGIER, Milan, Italy; 2 New York University and Hoover Institution, Stanford, USA

**Keywords:** Self-confirming equilibrium, Partial identification, Monetary policy

## Abstract

This paper provides a general framework for analyzing self-confirming policies. We study self-confirming equilibria in recurrent decision problems with incomplete information about the true stochastic model. We characterize stationary monetary policies in a linear-quadratic setting.

## Introduction

** Perspective** Policies often persist. Absent switching costs, the reason must be that the goals and beliefs of the policy maker also persist, which is possible only if long-run data coincide with what the policy maker expected. This belief-confirmation property does *not* imply that a persistent policy is justified by *correct* beliefs. Indeed, even if the stochastic consequences of the persistent policy are observable in the long run, a policy maker’s expectations about the consequences of alternative policies may be incorrect. We call *self-confirming* a policy justified by beliefs consistent with the long-run data affected by the policy itself. This paper provides a framework for the analysis of such self-confirming, stationary policies. We first develop a general analysis of self-confirming policies in recurrent decision problems with incomplete information about the true stochastic model. Next we apply and illustrate the theory with a characterization of stationary monetary policies in a linear-quadratic setting.

Consider an either moderately patient or impatient agent (she) who makes recurrent decisions under uncertainty. In each period she takes an action *a* that, via a feedback function *f*, delivers an observable outcome, or message, $m=f\left( a,s\right)$ that depends on an unobservable state of nature *s*. A fixed, unknown stochastic model $\sigma ^{*}$(that is, a probability measure over the states) determines an i.i.d. process of state realizations. The agent knows the feedback function *f*, but not the stochastic model $\sigma ^{*}$. Note that, for some action *a* , the same outcome *m* may result from multiple states, i.e., $f\left( a,\cdot \right)$ need not be injective; in this case, the realized outcome does not reveal the realized underlying state as exemplified below. There are no structural links between periods, but the agent observes the realized outcome in each period *t* and therefore updates her subjective belief $\mu _{t}$ about the fixed unknown model $\sigma ^{*}$. Over time, given a true model $\sigma ^{*}$ and a prior belief, the intertemporal subjective expected utility maximizing strategy yields a convergent active learning process, i.e., a stochastic process of actions and updated beliefs $\left( {\mathbf {a}}_{t},\varvec{\mu }_{t}\right)$ that converges almost surely.^1^ The realization $\left( a^{*},\mu ^{*}\right)$ of the stochastic limit satisfies almost surely the following two properties:

- Confirmed beliefs: $\mu ^{*}$ assigns probability 1 to the set of models $\sigma$ that are observationally equivalent to the true model $\sigma ^{*}$ *given* action $a^{*}$;^2^
- Subjective best reply: action $a^{*}$ maximizes the agent’s one-period subjective expected utility given belief $\mu ^{*}$.

We take “confirmed beliefs” and “subjective best reply” to be the characterizing properties of stationary actions and beliefs. We call * self-confirming equilibrium* an action-belief pair $\left( a^{*},\mu ^{*}\right)$ with these properties. Indeed, conceptually this is a special case of the self-confirming equilibrium idea of Battigalli (1987) and Fudenberg and Levine (1993a, b), applied to one-person games with incomplete information about the probabilities of states.^3^ The key observation is that the confirmed belief $\mu ^{*}$ need not assign probability one to the true stochastic model $\sigma ^{*}$ and, therefore, action $a^{*}$ may differ from the objective best reply to $\sigma ^{*}$. In other words, although equilibrium beliefs are disciplined by long-run empirical frequencies of observations, they do not necessarily concentrate on the true model $\sigma ^{*}$, so the long-run action $a^{*}$ may be objectively sub-optimal. This can happen even if the decision maker is quite patient: in the learning phase a positive discount factor can induce experimentation with actions that do not maximize one-period subjective expected utility, but the option value of experimentation vanishes in the limit.^4^

Consider the following heuristic example. A decision maker is asked to repeatedly bet on the color of a ball that will be drawn from an urn that contains 90 black, green, or yellow balls. After the draw, she is told whether she won (in which case she receives 1 euro) or not (in which case he receives 0 euros). Thus, there are three states, $S=\left\{ B,G,Y\right\}$, three actions $A=\left\{ b,g,y\right\}$, and two monetary outcomes $M=\left\{ 0,1\right\}$. The feedback function attains value 1 when the action matches the state ($f(B,b)=f\left( G,g\right) =f(Y,y)=1$) and value 0 otherwise. Thus, winning reveals the realized state, but losing only rules out one state out of three. Suppose the urn contains 45 black balls, 35 green balls, and 10 yellow balls, i.e., $\sigma ^{*}\left( B\right) =\frac{1}{2}$, $\sigma ^{*}\left( G\right) =\frac{7}{18}$, and $\sigma ^{*}\left( Y\right) =\frac{1}{9}$. The objective best reply is to bet on *B*, but the decision maker does not know this. Suppose she keeps betting on *G*. With high objective probability, she is going to win more than $\frac{1}{3}$ of the times and she may come to deem very likely that the urn contains more green than black or yellow balls. Indeed, betting on *G* infinitely many times she will almost certainly observe that the winning frequency is $\frac{7}{18}$ and in the long run her limit belief $\mu _{\infty }$ will assign probability 1 to the set models $\left\{ \sigma :\sigma \left( G\right) =\frac{7}{18}\right\}$. As long as her limit belief $\mu _{\infty }$ also assigns a sufficiently high probability to the set of models $\left\{ \sigma :\sigma \left( G\right) =\frac{7}{18}>\max \left\{ \sigma \left( B\right) ,\sigma \left( Y\right) \right\} \right\}$, she will find it optimal to keep betting on *G*, which is an objectively sub-optimal choice. Indeed, betting on *G* with such beliefs is a self-confirming equilibrium given the true model $\sigma ^{*}$. Even if she initially experiments betting on *B*, sufficiently many unlucky outcomes will dissuade her from doing it again. In other words, the vagaries of the active learning process may well drive her in the trap of choosing forever the “satisficing” action *g* that wins more that $\frac{1}{3}$ of the times rather than to experiment with *b* long enough to realize that it yields an even higher winning frequency.

Thus, in a self-confirming equilibrium, the decision maker may be best-replying to empirically confirmed but wrong views about the actual data generating model. She may thus get trapped in self-confirming behavior that differs substantially from the objectively optimal behavior postulated by rational expectations models.^5^ This trap and the resulting welfare loss is, at the same time, especially relevant and disturbing for policy making. It is relevant when policy makers cannot obtain enough reliable evidence before choosing (e.g., with externally valid lab or field experiments), but instead have to rely on evidence that is a by-product of their actual policies; it is disturbing because welfare in self-confirming equilibria can be lower than in rational expectations equilibria. The main contribution of the present paper is to provide a formal steady-state framework in which this important policy issue can be rigorously studied. We then use this framework and illustrate the macroeconomic relevance of our analysis in the context of a 70’s U.S. policy debate about whether there is a trade-off between inflation and unemployment that can be systematically exploited by a benevolent policy maker.

**Illustrative application** We consider a stylized model economy in which a policy maker chooses average inflation *a* and observes an unemployment/inflation outcome $\left( u,\pi \right) =f\left( a,s\right)$ that depends on the unobservable random state *s* of the economy. This model economy can be interpreted as reflecting an aggregate response function of a continuum of market agents. Assuming a quadratic loss function, we completely characterize the self-confirming equilibrium map that associates each conceivable model economy with a corresponding set of self-confirming beliefs and monetary policies. Given a fixed policy, the monetary authority infers from long-run data the first and second moments of the joint distribution of *u* and $\pi$, and hence the slope of the Phillips curve; but it cannot infer the true policy multiplier. We show that observing (in the long run) the distribution of $\left( u,\pi \right)$ leaves the monetary authority with a residual one-dimensional uncertainty about the model economy, parameterized by the direct impact of policy on unemployment (i.e., neglecting the impact on *u* through $\pi$).

For example, even if the true model is a rational expectations augmented Phillips curve, in equilibrium the monetary authority may believe that its policy does not shift the Phillips curve and hence that there is an exploitable trade-off given by the slope of the Phillips regression; the (Keynesian) monetary policy is optimal given a (falsely) conjectured trade-off, the subjectively expected unemployment rate coincides with the natural rate, and average inflation is (objectively) excessive. But we do not take a stand on what the true model is and so also consider self-confirming equilibria where the monetary authority pushes average inflation to zero falsely believing that there is no exploitable trade-off. Whatever the case, our analysis shows how partial identification may trap policy makers in inferior, yet self-confirming, policies that result in significant losses compared to the objectively optimal policies.

**Manifesto** Partial identification pervades economic policy debates: despite the use of sophisticated econometric techniques, economists disagree about how the economy works. Therefore, at least some economists must be wrong, but all of them should hold beliefs consistent with the data, which indeed only partially identify the relevant unknowns. The agents that inhabit our models —in particular, policy makers— are in a similar position, but their partial identification problem is exacerbated because what they can infer about the relevant unknowns depends on their own behavior, so it is endogenous. Thus, different policies justified by different beliefs —so, ultimately, by different (possibly conflicting) economic views— may be self-confirming. Such beliefs may even be dogmatic, for example because they assign probability one to a parameter vector resulting from observed long-run frequencies and untested, possibly false, identifying hypotheses.

To escape the partial identification trap more experimentation may be advisable. But we do not see an easy way out: large-scale social experiments can have huge costs, captured in our framework by the opportunity cost of not using a one-period subjective best reply, while small-scale ones may have little external validity.

**Roadmap** As anticipated, the first part of this paper (Sects. 2, 3, 4) develops an abstract analysis of self-confirming choices. The general contribution of this part is to provide a theoretical framework that is:

- *broad enough* to include the finite one-period setting in which self-confirming analysis was originally developed within game theory as well as the infinite setup relevant for many economic applications, including macroeconomic policy analysis;
- *specific enough* to provide welfare implications for relevant policy questions with the backdrop of a neat learning foundation.

The main issues that we address at this abstract level concern the scope of partial identification (Sect. 3), equilibrium values, and the resulting effects on the decision maker’s welfare (Sect. 4.2). The most novel results of the abstract part concern the latter topic. We show that if among two policies justified by different self-confirming beliefs one allows better identification of the true model, even if only partially, then this policy yields higher welfare. Similarly, self-confirming equilibria justified by sharper beliefs yield higher welfare. The theoretical concepts of this first part are illustrated and clarified by a running example of a monopolist facing an uncertain demand.

The second part of the paper (Sect. 5) builds on the abstract analysis to gain a better perspective and novel results on the classical debate on the possibility of systematically exploiting unemployment/inflation trade-offs. In particular, the scope of partial identification is characterized in Sect. 5.2, while equilibria, their values, and the welfare effects of model uncertainty are analyzed in Sect. 5.3. Sections 5.4 and 5.5 illustrate the analysis by considering two important special cases. Section 6 offers some concluding remarks.

Appendix A collects some more technical material and all the formal proofs.^6^

**Related literature** Our analysis contributes to and provides a bridge between two strands of literature, one in game theory and the other in macroeconomics, that are concerned with related issues, but have so far proceeded with limited cross fertilization and very different languages.

In the game-theoretic literature, a strategy profile that satisfies the properties of confirmed beliefs and subjective best reply has been called “conjectural equilibrium” (Battigalli, 1987; Battigalli & Guaitoli, 1988), “self-confirming equilibrium” (Fudenberg & Levine, 1993a) and “subjective equilibrium” (Kalai & Lehrer, 1993, 1995). Here we adopt the more self-explanatory terminology of Fudenberg and Levine. We refer the reader to Battigalli et al. (2015) for an up-to-date discussion of this literature. Here we point out that, although we focus on one-person decision problems with uncertainty, our abstract analysis extends seamlessly to *n*-person games except for the aforementioned comparative results about equilibrium values. To our knowledge, papers in the extant literature either consider finite (one-period) games, or games with no randomness. We extend the analysis of self-confirming equilibria to settings with inherent randomness and possibly infinite spaces of strategies and states of nature. Technically, this extension is not straightforward, it requires mathematical precision and care. We also point out that the learning foundation of the equilibrium concept is more solid in the one-person case analyzed here: while self-confirming equilibria of multi-person games represent the steady states of learning dynamics, convergence to a steady state is not guaranteed under general conditions, as is instead the case when the model can be effectively reduced to a one-person decision problem. Finally, note that Battigalli et al. (2015) is focused on the interaction between ambiguity aversion and self-confirming equilibria in games. Here instead we consider a decision maker who maximizes her subjective expected utility (i.e., she is ambiguity neutral). This simplifies the general analysis without affecting the illustrative examples and the application. Indeed, they feature conditions under which the degree of ambiguity aversion does not affect the set of self-confirming equilibria (see Sect. 6 and Battigalli et al., 2021), although it may well affect learning dynamics and the likelihood to be trapped in the long run in self-confirming equilibria with an objectively suboptimal choice (see Battigalli et al., 2019).

The macroeconomic literature focuses on policy making and learning dynamics. Sargent (1999) explains the rise and fall of US inflation assuming that the monetary authority sequentially estimates a Phillips curve, ignoring its impact on expectations, and best replies to updated beliefs. Standard OLS estimation leads to a Keynesian self-confirming equilibrium, but if instead recent observations are given more weight, because the monetary policy maker’s decisions make the Phillips curve slowly shift and rotate over time, the process first approaches a neighborhood of this equilibrium, but then recurrently abandons it when the Phillips curve looks “more vertical” leading the monetary policy maker to lower inflation.^7^ Cho et al. (2002) and Sargent and Williams (2005) sharpen the theoretical analysis of such learning dynamics.^8^ Cho and Kasa (2015) note that the low inflation outcome at the end of Sargent’s (1999) narrative —according to the postulated learning model— cannot persist either; therefore, they consider an alternative stochastic learning dynamic in which the policy maker best responds to the current estimate of an aggregate supply model, out of a set of conceivable functional forms, as long as the model passes a statistical test; when the model is rejected, a new model is selected at random and the process is restarted. Also, in their model the Keynesian self-confirming equilibrium cannot persist, because, in the very long-run, the monetary authority adopts a vertical Phillips curve model.^9^ In our paper, we focus only on the set of possible limit points of learning dynamics. Furthermore, in our monetary policy application, we follow Sargent (2008) and assume that the monetary authority may believe in the exploitability of a trade-off between unemployment and inflation. Unlike the papers we have mentioned, we do not take a stand on a true model economy. Thus, instead of assuming that the true model economy features a rational-expectations augmented Phillips curve, we characterize the self-confirming equilibria and values for many conceivable models.

Other papers in the literature focus, like ours, mainly on self-confirming equilibrium policies rather than learning dynamics. In particular, Battigalli and Guaitoli (1988) analyze the self-confirming equilibria with rationalizable beliefs of a stylized policy game with incomplete information, showing that there are equilibria with Keynesian features and equilibria with new-classical features. Fudenberg and Levine (2009) discuss the Lucas critique through the analysis of refined self-confirming equilibria in some insightful illustrative examples; they emphasize the role of rationalizable beliefs and of robustness to experimentation. Unlike the foregoing papers, we formally analyze a one-agent framework, which makes the issue of the rationalizability of beliefs mute. According to the application, when the one-agent framework is interpreted as a reduced form of a multi-person game, the shape of outcome/feedback function *f* may implicitly represent such rationalizability constraints, e.g., the decision maker is a leader and the outcome function captures the best-reply behavior of followers; this is clarified by our monopoly example. As for the monetary policy application, only a genuinely game-theoretic model of the economy would allow a thorough analysis of the rationalizability of self-confirming beliefs, but tackling such difficult issue is beyond the scope of this article. In a series of papers, Saint Paul (e.g., 2013, 2018) considers an expert who knows the true model and advises the policy maker while pursuing her own policy agenda; the policy maker and the agents in the market fully trust the expert as long as the data are consistent with her advice. With this, the expert manipulates the policy maker and market agents under a self-confirmation constraint. Finally, Gaballo and Marimon (2021) analyze a directed search model of the credit market where lenders post excessively high interest rates because of confirmed pessimistic beliefs about returns on investments, but the monetary authority can break the spell by easing credit. The main difference with our monetary policy application is that we study the self-confirming actions and beliefs of the monetary authority, not of the agents in the market.^10^

To the best of our knowledge, besides the novelty of several results, our paper is unique in integrating an abstract analysis of self-confirming policies with an economic application. There are under-appreciated complementarities between abstract theory and applications. The former allows to focus on key concepts and properties uncluttered by specific modeling features, the latter helps to better understand the abstract theory and points to its relevance. Here we consider a monetary policy application, but the scope of our analysis goes well beyond that. For example, the difficulty of thorough experimentation and its consequences for welfare naturally arise in the context of environmental policies.

## Preliminaries

### Mathematics

Differently from Battigalli et al. (2015), the Phillips curve exploitation model that motivates and illustrates this paper features infinite action, state, and consequence spaces as well as unbounded payoff functions. The necessary adaptation is conceptually natural, but technically nontrivial. In particular, it requires that the analysis be carried out within a *standard Borel space *$\left( X,{\mathcal {X}}\right)$, where *X* is a completely metrizable and separable topological space and $\mathcal { X}$ is its Borel sigma algebra. The Borel sets $B\in {\mathcal {X}}$ are themselves standard Borel spaces under the relative sigma algebra $\mathcal {X }\cap B$.^11^ When *X* is countable (i.e., finite or denumerable), standardness requires ${\mathcal {X}}$ to be the power set of *X* (see Appendix A.1).

We denote by $\Delta \left( X\right)$ the collection of all probability measures on ${\mathcal {X}}$, endowed with the natural sigma algebra,^12^ which in turn makes $\Delta \left( X\right)$ a standard Borel space too. With this, the Borel subsets $\Sigma$ of $\Delta \left( X\right)$ with their relative sigma algebras are standard Borel spaces themselves. The meaning of $\Delta \left( \Sigma \right)$ is then obvious. Finally, $\delta :X\rightarrow \Delta \left( X\right)$ denotes the canonical Dirac embedding of *X* into $\Delta \left( X\right)$, that is, $\delta \left( x\right)$ is the probability measure on ${\mathcal {X}}$ which assigns probability 1 to each Borel set containing $x\in X$.^13^

Let $\left( Y,{\mathcal {Y}}\right)$ be another standard Borel space. The Cartesian product $X\times Y$ is a standard Borel space with respect to the product sigma algebra. Moreover, each measurable function $\varphi :X\rightarrow Y$ induces a measurable distribution map ${\hat{\varphi }}:\Delta (X)\rightarrow \Delta (Y)$ defined by

$$\begin{aligned} {\hat{\varphi }}(\xi )=\xi \circ \varphi ^{-1} \end{aligned}$$

for each probability measure $\xi \in \Delta (X)$. That is, ${\hat{\varphi }} (\xi )\left( B\right) =\xi (\varphi ^{-1}(B))$ for all sets *B* in $\mathcal { Y}$.^14^

#### Lemma 1

*Let* $\varphi :X\rightarrow Y$ *be a measurable function. The following conditions are equivalent:*

(i) $\varphi$ *is one-to-one,*
(ii) ${\hat{\varphi }}$ *is one-to-one,*
(iii) $$\varphi ^{-1}\left( {\mathcal {Y}}\right) ={\mathcal {X}}$$.

Interpreting *x* as a state and $y=\varphi \left( x\right)$ as an observable outcome, we can phrase these equivalent conditions as follows:

(i) $\varphi$ reveals the state *x* in *X*,
(ii) ${\hat{\varphi }}$ reveals the distribution $\xi$ in $\Delta (X)$,
(iii) $\varphi$ generates the sigma-algebra ${\mathcal {X}}$.

Finally, we say that *X* and *Y* are *isomorphic*, written $X\cong Y$, if there is a bimeasurable bijection $\varphi :X\rightarrow Y$, that is, $\varphi$ is measurable and $\varphi ^{-1}:Y\rightarrow X$ is a well defined measurable function.

### Classical subjective expected utility

Let *S* be a space of *states* of nature, *A* a space of *actions* available to the decision maker, *C* a space of *consequences*, and $\rho :A\times S\rightarrow C$ a measurable *consequence function* that associates a consequence $\rho \left( a,s\right) \in C$ with each pair $\left( a,s\right) \in A\times S$ of action and state. When consequences are monetary, *C* is a (Borel) subset of the real line.

The quartet

1 $$\begin{aligned} \left( A,S,C,\rho \right) \end{aligned}$$

is the basic structure of the decision problem. The inherent randomness characterizing the realization of states —often called physical uncertainty— is described by probability models $\sigma \in \Delta \left( S\right)$ that can be regarded as possible generative mechanisms. For each probability model $\sigma$, actions *a* are evaluated through their expected utility

$$\begin{aligned} \int _{S}v\left( \rho \left( a,s\right) \right) d\sigma \left( s\right) \end{aligned}$$

where $v:C\rightarrow {\mathbb {R}}$ is a measurable and bounded above von Neumann-Morgenstern utility function. It is often convenient to write the criterion in the expected-payoff form

$$\begin{aligned} R\left( a,\sigma \right) =\int _{S}r\left( a,s\right) d\sigma \left( s\right) \end{aligned}$$

where $r:A\times S\rightarrow {\mathbb {R}}$ is the *payoff* (or * reward*) *function* $r=v\circ \rho$. Also the payoff function is easily seen to be measurable and bounded above. All our integrals are thus well defined, but may take value $-\infty$.

The decision maker may not know the true probability model $\sigma ^{*}$ but is able to posit a (measurable) collection $\Sigma \subseteq \Delta \left( S\right)$ of probability models that contains the true one; that is, $\sigma ^{*}\in \Sigma$. We thus abstract from misspecification issues. We call *structural* the kind of information that allows the decision maker to posit the collection $\Sigma$. For example, if the problem is to bet on the color, white or black, of a ball drawn from a two-color urn, and it is only known that the urn contains *n* balls, then $\Sigma$ has $n+1$ elements and is isomorphic to the set $\left\{ 0,\frac{1}{n},\ldots ,\frac{n-1}{ n},1\right\}$ of possible fractions of white balls. When $\Sigma$ is a singleton, i.e., the true model is known, the decision maker confronts only *risk*. Otherwise, she faces *model uncertainty*.^15^ We can also give $\Sigma$ a somewhat different interpretation: it represents a *backdrop theory* accepted by the decision maker, which happens to be correct (i.e., such that $\sigma ^{*}\in \Sigma$). In particular, as we assume that the same decision problem is faced infinitely often, representing uncertainty with $\Sigma$ rests on the assumption that the process of states is i.i.d.^16^

The decision maker ranks actions according to the *classical subjective expected utility *(SEU) criterion:^17^

2 $$\begin{aligned} V\left( a,\mu \right) =\int _{\Sigma }R\left( a,\sigma \right) d\mu \left( \sigma \right) , \end{aligned}$$

where $\mu \in \Delta \left( \Sigma \right)$ is a subjective *prior probability *over models that reflects personal beliefs about models that the decision maker may have, in addition to the structural information behind $\Sigma$.^18^ This representation admits the reduced form

$$\begin{aligned} \int _{\Sigma }R\left( a,\sigma \right) d\mu \left( \sigma \right) =\int _{S}r\left( a,s\right) d\sigma _{\mu }\left( s\right) =R\left( a,\sigma _{\mu }\right) , \end{aligned}$$

where $\sigma _{\mu }\in \Delta \left( S\right)$ is the subjective *predictive probability* defined by $\sigma _{\mu }\left( E\right) =\int _{\Sigma }\sigma \left( E\right) d\mu \left( \sigma \right)$ for each $E\in {\mathcal {S}}$. This reduced form is the original representation of Savage (1954), who derived $\sigma _{\mu }$ from preferences over bets.

The *decision problem* can then be summarized by the sextet

3 $$\begin{aligned} D=\left( A,S,C,\rho ,\Sigma ,v\right) \end{aligned}$$

that combines the basic structure (1) with the information and taste traits $\Sigma$ and *v*. A few special cases are noteworthy.

(i) When the support of $\mu$ is a singleton $\left\{ \sigma \right\}$, that is, $\mu =\delta \left( \sigma \right)$, the decision maker believes (maybe wrongly) that $\sigma$ is the true model. The predictive probability trivially coincides with $\sigma$ and criterion (2) reduces to the Savage expected payoff criterion $R\left( a,\sigma \right)$. Being a predictive probability, $\sigma$ here is a subjective probability measure, albeit one derived from a dogmatic belief.
(ii) When $\Sigma$ is a singleton $\left\{ \sigma ^{*}\right\}$, the decision maker has maximal structural information and, as a result, knows that $\sigma ^{*}$ is the true model. In this case, there is only physical uncertainty, quantified by $\sigma ^{*}$, without any model uncertainty. Criterion (2) again reduces to the expected payoff criterion $R\left( a,\sigma ^{*}\right)$, but now interpreted as a von Neumann-Morgenstern criterion. For instance, if the decision maker either observed infinitely many draws from a given urn or were just able to count the balls of each color, she would learn/know the urn composition and $\Sigma$ would be a singleton.
(iii) When $\Sigma \subseteq \left\{ \delta \left( s\right) :s\in S\right\}$, there is no physical uncertainty, but only model uncertainty, quantified by $\mu$. We can identify prior and predictive probabilities: with a slight abuse of notation, we can write $\mu \in \Delta \left( S\right)$ and so (2) takes the form $R\left( a,\mu \circ \delta \right)$.^19^

Throughout this part (Sects. 2, 3, 4), we illustrate the abstract theoretical concepts with a stripped-down monopoly example.

#### Example 1

(*Monopoly: Unknown demand*) A monopolist choosing output $a\ge {\underline{a}}$ faces an imperfectly known (state-dependent) inverse demand function $a\mapsto P\left( a,s\right)$. We interpret the lower bound ${\underline{a}}\ge 0$, when strictly positive, as a pre-commitment to a minimum level of production. If ${\underline{a}}=0$ there is no pre-commitment. The firm knows the slope, but not the intercept, which has a permanent component $\theta$ modified by an additive noise $\varvec{\varepsilon }$:

$$\begin{aligned} P\left( a,s\right) =\max \left\{ 0,s-a\right\} =\max \left\{ 0,\theta +\varepsilon -a\right\} \text {,} \end{aligned}$$

where $s\in S=\left[ {\underline{s}},{\bar{s}}\right] \subseteq {\mathbb {R}}_{++}$, $\theta \in \left[ {\underline{\theta }},{\bar{\theta }}\right] =\left[ {\underline{s}}+{\bar{\varepsilon }},{\bar{s}}-{\bar{\varepsilon }}\right]$, and $\varepsilon \in \left[ -{\bar{\varepsilon }},{\bar{\varepsilon }}\right]$ is the realization of a random variable $\varvec{\varepsilon }$ with *known* distribution $\eta$ and 0 mean.^20^ The firm has a known linear cost function, with average and marginal cost $c>0$. To further simplify the analysis, we assume that *price is certainly strictly positive on the relevant range of outputs*, i.e., also for the largest subjective best reply across all possible beliefs. This is the case if^21^

$$\begin{aligned} {\underline{\theta }}-\frac{3}{2}{\bar{\varepsilon }} -\frac{{\bar{\theta }}-c}{2}>0 . \end{aligned}$$

With this, we can ignore the 0-price floor, and the relevant inverse demand map becomes $a\mapsto \left( \theta +\varepsilon -a\right)$. For each $\theta \in \left[ {\underline{\theta }},{\bar{\theta }}\right]$, let $T_{\theta }:\left[ -{\bar{\varepsilon }},{\bar{\varepsilon }}\right] \rightarrow S$ denote the translation map $\varepsilon \mapsto \left( \theta +\varepsilon \right)$. We can parameterize $\Sigma$ as follows:^22^

$$\begin{aligned} \Sigma =\left\{ \sigma \in \Delta \left( S\right) :\exists \theta \in \left[ {\underline{\theta }},{\bar{\theta }}\right] ,\sigma =\eta \circ T_{\theta }^{-1}\right\} \cong \left[ {\underline{\theta }},{\bar{\theta }}\right] \text {.} \end{aligned}$$

The consequence function (again, in the relevant range of outputs) is the profit function

$$\begin{aligned} \rho \left( a,s\right) =a\left( s-a-c\right) . \end{aligned}$$

Under *risk neutrality*, *v* is the identity on the range of $\rho$; thus, $r=\rho$. Given the parameterization of $\Sigma$, the objective expected payoff and subjective expected utility can be written as

$$\begin{aligned} R\left( a,\theta \right) ={\mathbb {E}}\left( a\left( \theta +\varvec{\varepsilon }-a-c\right) \right) =a\left( \theta -a-c\right) \end{aligned}$$

and

$$\begin{aligned} V\left( a,\mu \right) =\int _{{\underline{\theta }}}^{{\bar{\theta }}}a\left( \theta -a-c\right) d\mu \left( \theta \right) =\left( {\mathbb {E}}_{\mu }\left( \theta \right) -c\right) a-a^{2}\text {.} \end{aligned}$$

We obtain case (i) if the firm is certain of $\theta$, case (ii) if it knows $\theta ^{*}$, and case (iii) if there is no noise, i.e., $\bar{ \varepsilon }=0$ and $S=\left[ {\underline{\theta }},{\bar{\theta }}\right]$.

The example clarifies that decision problem *D* could be the reduced form of a multi-agent model where the unknown state *s* represents the behavior of other agents, such as buyers. Such behavior is unaffected by choice *a*, either literally, or because it represents a profile of strategies (decision functions) rather than actual actions. In the quantity-setting monopoly, *s* may be determined by a distribution of private valuations, with output *a* sold in a multi-unit uniform price auction and with unit-demand buyers bidding their valuations as their dominant bid. For a price-setting monopolist valuations determine individual demand functions as optimal reactions to the set price. In these cases, the map $a\mapsto \rho _{s}\left( a\right)$ is determined by rational behavior of the un-modeled agents. In an alternative interpretation, the firm is a monopolistic competitor of negligible size and *s* represents general market conditions.

## Partial identification

### Feedback

The decision maker faces decision problem *D* recurrently in a stationary environment with an i.i.d. process of states determined by unknown probability model $\sigma ^{*}$. To determine what actions and beliefs can be stable given $\sigma ^{*}$, we have to specify the information obtained ex post by the decision maker for each action *a* and state *s*. We model such information through a (measurable) *feedback function*

$$\begin{aligned} f:A\times S\rightarrow M \end{aligned}$$

where *M* is a space of messages. By selecting an action $a\in A$, the decision maker receives a *message*

$$\begin{aligned} m=f_{a}\left( s\right) \end{aligned}$$

when *s* occurs.^23^ The decision maker’s (ex post) information about the state is thus endogenous. When *M* is finite, such endogenous information is represented by the partition $\left\{ f_{a}^{-1}\left( m\right) :m\in M\right\}$ of the state space *S* that the messages induce, which depends on the choice of action *a*. This partition generates the algebra of events whose probability can be inferred from the long-run frequencies of messages. When *M* is infinite, it may be the case that this collection of events cannot be recovered from the partition. Hence, it is technically convenient to represent information with the sigma algebra

$$\begin{aligned} {\mathcal {F}}_{a}=\left\{ f_{a}^{-1}\left( B\right) :B\in {\mathcal {M}}\right\} . \end{aligned}$$

A *decision problem with feedback* is described by the octet

4 $$\begin{aligned} \left( A,S,C,\rho ,\Sigma ,v,M,f\right) \end{aligned}$$

where a feedback function and a message space are added to the decision problem (3).

When information does not depend on action *a*, we say that there is *own-action independence* of feedback about the state; formally, ${\mathcal {F}} _{a}={\mathcal {F}}_{a^{\prime }}$ for all $a,a^{\prime }\in A$. The most important instance of own-action independence is *perfect feedback*, which occurs when each section $f_{a}$ of the feedback function *f* generates ${\mathcal {S}}$ —that is, in view of Lemma 1, when $f_{a}$ is one-to-one for each $a\in A$. In this case, messages reveal to the decision maker which state obtained, regardless of the chosen action. When this is not the case, feedback about the state is *imperfect*, maximally so when each section $f_{a}$ is constant, so that ${\mathcal {F}} _{a}=\left\{ \emptyset ,S\right\}$ and all states return the same message.

An action *a* is *fully revealing* if $f_{a}$ is one-to-one, that is, if it allows the decision maker to learn which state obtained. Under perfect feedback, all actions are fully revealing. The existence of fully revealing actions is a weak form of “endogenous” perfect feedback.

We assume throughout that *consequences are observable*. Formally, this amounts to assuming that, for each action $a\in A$, the section $\rho _{a}$ of the consequence function $\rho$ is ${\mathcal {F}}_{a}$-measurable. The next result, which will play an important role in our analysis, characterizes this assumption within a decision problem with feedback (4).

#### Proposition 1

*Consequences are observable if and only if, for each action* $a\in A$*, there exists a measurable function* $g_{a}:M\rightarrow C$ *such that*

$$\begin{aligned} \rho _{a}=g_{a}\circ f_{a}\text {.} \end{aligned}$$

*In this case, the payoff* $r_{a}=v\circ \rho _{a}$ *of each action* *a* *is* ${\mathcal {F}}_{a}$-*measurable.*

In words, messages encode consequences and so payoffs. In particular, when the consequences of the actions are the only observed messages, we have $C=M$ and $f=\rho$. This is the most common and important case of feedback, which is also featured by our macroeconomic application.

#### Example 2

(*Monopoly: Feedback*) A natural assumption about feedback for the quantity-setting monopoly of Example 1 is that the firm observes the realized market price, that is (for the relevant range of outputs)

$$\begin{aligned} f\left( a,s\right) =P\left( a,s\right) =s-a \end{aligned}$$

and $g_{a}$ is the affine map $p\mapsto a\left( p-c\right)$ from market price to profit. Note, however, that if the firm has a zero lower bound ${\underline{a}}=0$, assuming that a realized price can be observed even with 0 output to sell is contrived; indeed, the most plausible assumption is that nothing is observed. The same observability pattern occurs with an alternative assumption about feedback: the firm only observes its revenue, $f\left( a,s\right) =\rho \left( a,s\right) +ca$, e.g., because it is the grower of a unique variety of weed sold to a dealer who returns the proceeds from an auction. With 0 production, nothing can be observed, with positive production $a>0$, the unit price and realized state can be backed out: $p=\rho /a+c$ (per-unit revenue) and $s=p+a$. To sum up, both feedback functions satisfy observability of consequences, and each interior choice ($a>0$) is revealing. Thus, own-action independence of feedback about the state holds if there is a strictly positive lower bound on production ${\underline{a}}>0$. Absent this constraint (${\underline{a}}=0$), own-action independence of feedback about the state fails because 0-output reveals nothing, while positive output is revealing.

### Partial identification correspondence

In our steady state setting, a message distribution $\nu \in \Delta \left( M\right)$ can be interpreted as a long-run empirical frequency of messages received by the decision maker. Specifically, for each Borel set $B\in {\mathcal {M}}$, $\nu \left( B\right)$ is the long-run empirical frequency with which messages *m* belong to *B*. For any action $a\in A$, consider the distribution map ${\hat{f}}_{a}:\Sigma \rightarrow \Delta \left( M\right)$ defined by ${\hat{f}}_{a}\left( \sigma \right) =\sigma \circ f_{a}^{-1}$. That is,

$$\begin{aligned} {\hat{f}}_{a}\left( \sigma \right) \left( B\right) =\sigma \left( s\in S:f_{a}\left( s\right) \in B\right) \end{aligned}$$

for each $B\in {\mathcal {M}}$.^24^ Then ${\hat{f}}_{a}\left( \sigma \right) \left( B\right)$ is the long-run empirical frequency with which the decision maker receives messages *m* in *B*, when action *a* is chosen and $\sigma$ is the true model. The inverse correspondence ${\hat{f}}_{a}^{-1}$ from $\Delta \left( M\right)$ to $\Delta \left( S\right)$ partitions the latter set into classes

$$\begin{aligned} {\hat{f}}_{a}^{-1}\left( \nu \right) =\left\{ \sigma \in \Delta \left( S\right) :{\hat{f}}_{a}\left( \sigma \right) =\nu \right\} \end{aligned}$$

of models that are observationally equivalent given that action *a* is chosen infinitely often and that the frequency distribution of messages $\nu$ is observed in the long-run conditional on *a*. In other words, ${\hat{f}} _{a}^{-1}\left( \nu \right)$ is the collection of all probability models that may have generated $\nu$ given *a*.

If action *a* is fully revealing, then ${\hat{f}}_{a}$ is one-to-one and so ${\hat{f}}_{a}^{-1}\left( \nu \right)$ is at most a singleton for every $\nu$ . In this case the decision problem is identified under *a* since different models generate different message distributions, which thus uniquely pin down models. Otherwise, when ${\hat{f}}_{a}^{-1}\left( \nu \right)$ is nonsingleton for some $\nu$, we have *partial identification* under action *a*. In the extreme case when ${\hat{f}}_{a}$ is constant —that is, when all models generate the same message distribution— the decision problem is completely unidentified under action *a*. Interestingly, ${\hat{f}} _{a}$ is constant if and only if $f_{a}$ is constant, that is, all states generate the same message (see Lemma 6 in Appendix A.1).

Now recall that the decision maker posits a set of models $\Sigma$ determined by structural information or a backdrop theory. Upon observing $\nu$, one can conclude that the data generating model belongs to

$$\begin{aligned} \underset{\text {data}}{\underbrace{{\hat{f}}_{a}^{-1}\left( \nu \right) }}\cap \underset{\text {theory}}{\underbrace{\Sigma }}\text {.} \end{aligned}$$

For this reason, models $\sigma$ and $\sigma ^{\prime }$ in $\Sigma$ such that ${\hat{f}}_{a}\left( \sigma \right) ={\hat{f}}_{a}(\sigma ^{\prime })=\nu$ are observationally equivalent under action *a*. Formally, given an action *a*, two models $\sigma ,\sigma ^{\prime }\in \Sigma$ are * observationally equivalent *if

$$\begin{aligned} {\hat{f}}_{a}\left( \sigma \right) ={\hat{f}}_{a}(\sigma ^{\prime }). \end{aligned}$$

We denote the class of models observationally equivalent to $\sigma$ given *a* by

5 $$\begin{aligned} {\hat{\Sigma }}_{a}\left( \sigma \right) =\left\{ \sigma ^{\prime }\in \Sigma : {\hat{f}}_{a}(\sigma ^{\prime })={\hat{f}}_{a}\left( \sigma \right) \right\} = {\hat{f}}_{a}^{-1}({\hat{f}}_{a}\left( \sigma \right) )\cap \Sigma \text {.} \end{aligned}$$

In other words, ${\hat{\Sigma }}_{a}\left( \sigma \right)$ is the * partially identified set* of models given action *a*.^25^ We can thus regard the map ${\hat{\Sigma }} _{a}\left( \cdot \right) :\Sigma \rightrightarrows \Sigma$, which associates to each element of $\Sigma$ its observational equivalence class, as the *partial* *identification correspondence* determined by action *a*.

It is easy to see that ${\hat{\Sigma }}_{a}$ has convex values if the collection $\Sigma$ is convex. Moreover, if ${\hat{f}}_{a}$ is one-to-one, then ${\hat{\Sigma }}_{a}$ is the identity: ${\hat{\Sigma }}_{a}\left( \sigma \right) =\left\{ \sigma \right\}$ for all $\sigma \in \Sigma$. In this case, message distributions identify the true model. In contrast, when $\hat{ \Sigma }_{a}\left( \sigma \right)$ is nonsingleton there is genuine partial identification.

Summing up, the collection $\{{\hat{\Sigma }}_{a}(\sigma )\}_{\sigma \in \Sigma }$ is a measurable partition of $\Sigma$ and its cells consist of probability models that are observationally equivalent under action *a*. Clearly, the dependence on *a* is lost under own-action independence of feedback about the state.

#### Example 3

(*Monopoly: Partial Identification*) Consider the quantity-setting monopoly of Example 1. If feedback is the realized price and output $a>0$ is chosen infinitely often, the firm observes in the long-run the average price ${\mathbb {E}}\left( {\mathbf {p}} _{a}\right) =\theta -a$, and $\theta$ is identified: $\theta ={\mathbb {E}} \left( {\mathbf {p}}_{a}\right) +a$. With a 0-lower bound (${\underline{a}}=0$ ), producing 0 instead reveals nothing. Thus, given the parameterization of $\Sigma$, the partial identification correspondence is

$$\begin{aligned} {\hat{\Sigma }}_{a}\left( \sigma \left( \theta \right) \right) \cong \hat{\Theta }_{a}\left( \theta \right) =\left\{ \begin{array}{ll} \left\{ \theta \right\} &{} \quad {\text{if}}\, a>0, \\ \left[ {\underline{\theta }},{\bar{\theta }}\right] &{} \quad {\text{if}}\, a=0. \end{array} \right. \end{aligned}$$

### Comparative statics

The extent of partial identification depends, intuitively, on how informative is the underlying feedback function. To formalize this intuition, we need to compare feedback functions according to their informativeness. To this end, we say that a feedback function $f^{\prime }$ is *coarser* (or less *fine*) than a feedback function *f* if, for each $a\in A$, $f_{a}^{\prime }$ is ${\mathcal {F}}_{a}$-measurable or, equivalently, if there exists a measurable function $h_{a}:M\rightarrow M^{\prime }$ such that

$$\begin{aligned} f_{a}^{\prime }=h_{a}\circ f_{a}. \end{aligned}$$

In the monopoly example, with a 0-lower bound (${\underline{a}}=0$) and under the assumption that a notional realized price could be observed even at 0 output, realized revenue/profit $f_{a}^{\prime }\left( s\right) =\rho _{a}\left( s\right)$ is a coarser feedback than realized price $f_{a}\left( s\right) =P_{a}\left( s\right)$, with $h_{a}\left( p\right) =ap$.

A coarser feedback function is less informative. Using this comparative notion, we show that a less informative feedback function aggravates the decision maker’s partial identification problem, thus formalizing the previous intuition. Given feedback functions *f* and $f^{\prime }$, we let ${\hat{\Sigma }}_{a}\left( \cdot \right)$ and ${\hat{\Sigma }}_{a}^{\prime }\left( \cdot \right)$ denote the identification correspondences respectively derived from *f* and $f^{\prime }$.

#### Proposition 2

*Fix feedback functions* *f* *and* $f^{\prime }$. *If* $f^{\prime }$ *is coarser than* *f, then* ${\hat{\Sigma }}_{a}\left( \sigma \right) \subseteq {\hat{\Sigma }}_{a}^{\prime }\left( \sigma \right)$ *for all* $a\in A$ *and* $\sigma \in \Sigma$.

Coarser feedback functions thus determine, for each action, coarser observational equivalence relations: a worse information translates into a lower degree of statistical identification. In particular, the assumption that consequences are observable makes the consequence function $\rho$ the coarsest possible feedback. Perfect feedback is, instead, the finest.

## Self-confirming actions and beliefs

### Definition

Throughout this section, we fix a decision problem

$$\begin{aligned} \left( A,S,C,\rho ,\Sigma ,v,M,f\right) \end{aligned}$$

with feedback and observable consequences, where $\Sigma$ contains the true model $\sigma ^{*}$ that generates the states.

With this, we introduce a concept that is at the heart of our analysis and is motivated by the partial identification issues discussed in the previous section.

#### Definition 1

An action-belief pair $\left( a^{*},\mu ^{*}\right) \in A\times \Delta \left( \Sigma \right)$ is a *self-confirming equilibrium* given $\sigma ^{*}$ if

6 $$\begin{aligned} \forall a\in A,\quad V\left( a^{*},\mu ^{*}\right) \ge V\left( a,\mu ^{*}\right) \end{aligned}$$

and

7 $$\begin{aligned} \mu ^{*}\in \Delta ({\hat{\Sigma }}_{a^{*}}\left( \sigma ^{*}\right) ). \end{aligned}$$

The definition relies on two pillars: the optimality condition (6) that ensures that action $a^{*}$ is subjectively optimal under belief $\mu ^{*}$, and the belief confirmation condition (7) that guarantees that belief $\mu ^{*}$ is consistent with the data that action $a^{*}$ reveals in the long run.^26^ In fact, given model $\sigma ^{*}$, action $a^{*}$ determines the message distribution $\nu ^{*}={\hat{f}}_{a^{*}}\left( \sigma ^{*}\right)$, which is the long-run evidence that disciplines the subjective belief $\mu ^{*}$. In this respect, note that

$$\begin{aligned} {\hat{\Sigma }}_{a^{*}}\left( \sigma ^{*}\right) ={\hat{f}}_{a^{*}}^{-1}\left( {\hat{f}}_{a^{*}}\left( \sigma ^{*}\right) \right) \cap \Sigma ={\hat{f}}_{a^{*}}^{-1}\left( \nu ^{*}\right) \cap \Sigma \text { .} \end{aligned}$$

Therefore, ${\hat{\Sigma }}_{a^{*}}\left( \sigma ^{*}\right)$ depends only on the induced message distribution $\nu ^{*}$.

Note also that condition (7) makes self-confirming equilibrium for decision problems with feedback a *genuine* equilibrium concept. Indeed, we already mentioned in the Introduction that it characterizes the steady states of learning dynamics in stochastic control problems. Relatedly, it is a fixed-point concept: suppose for simplicity that there is a unique best reply $B\left( \mu \right)$ for each belief $\mu$; then, a self-confirming belief is a *fixed point* of the correspondence

$$\begin{aligned} \mu \mapsto \Delta ({\hat{\Sigma }}_{B\left( \mu \right) }\left( \sigma ^{*}\right) )\text {.} \end{aligned}$$

Finally, it is worth noting that a self-confirming belief may exclude the true model.^27^ We can indeed formulate the data confirmation condition (7) as follows:

8 $$\begin{aligned} \underset{\text {belief}}{\underbrace{\mu ^{*}}}\left( \underset{\text { data}}{\underbrace{{\hat{f}}_{a^{*}}^{-1}\left( \nu ^{*}\right) }}\cap \underset{\text {theory}}{\underbrace{\Sigma }}\right) =1\text {.} \end{aligned}$$

The equilibrium belief must thus exclude everything which is not consistent with either observations or structural information/theory, that is,

9 $$\begin{aligned} \underset{\text {belief}}{\underbrace{\mu ^{*}}}\left( \underset{\text { contra data}}{\underbrace{{\hat{f}}_{a^{*}}^{-1}\left( \nu ^{*}\right) ^{c}}}\cup \underset{\text {contra theory}}{\underbrace{\Sigma ^{c}}}\right) =0\text {,} \end{aligned}$$

but it may exclude other models as well, including the true one.

Under own-action independence of feedback about the state, the data confirmation condition (7) becomes $\mu ^{*}\in \Delta ( {\hat{\Sigma }}\left( \sigma ^{*}\right) )$. We thus return to a traditional optimization notion with a purely exogenous data confirmation condition. In particular, under perfect feedback —and so full identification— the optimality condition (6) becomes

10 $$\begin{aligned} \forall a\in A,\quad R\left( a^{*},\sigma ^{*}\right) \ge R\left( a,\sigma ^{*}\right) , \end{aligned}$$

since condition (7) requires $\mu ^{*}=\delta \left( \sigma ^{*}\right)$. In this case, common in the rational expectations literature, the decision maker has a correct belief about the true model and confronts only risk.

We say that an action $a^{*}\in A$ is *objectively optimal* given $\sigma ^{*}$ if it satisfies the optimality condition (10). Objectively optimal actions are the ones that the decision maker would select if she knew the true model, that is, under full identification. As such, they provide an important benchmark to assess alternative courses of action, as the next welfare analysis will show.

That said, observe that a “rational-expectations” pair $\left( a^{*},\delta \left( \sigma ^{*}\right) \right)$, where action $a^{*}$ is objectively optimal and belief $\delta \left( \sigma ^{*}\right)$ is concentrated on the true model, is a self-confirming equilibrium. Indeed, $\sigma ^{*}\in {\hat{\Sigma }}_{a^{*}}\left( \sigma ^{*}\right)$ and so $\delta \left( \sigma ^{*}\right) ({\hat{\Sigma }}_{a^{*}}\left( \sigma ^{*}\right) )=1$. Traditional rational-expectations analysis can thus be seen as the special case of ours that arises when the decision maker confronts only risk.

We close the section with a useful equivalence result. The optimality condition (6) can be written in predictive form as $R\left( a^{*},\sigma _{\mu ^{*}}\right) \ge R\left( a,\sigma _{\mu ^{*}}\right)$ for each $a\in A$. Relatedly, the data confirmation condition ( 7) implies that the predictive probability $\sigma _{\mu ^{*}}$ belongs to ${\hat{\Sigma }}_{a^{*}}\left( \sigma ^{*}\right)$ if it belongs to $\Sigma$.^28^ In this case, $(a^{*},\delta \left( \sigma _{\mu ^{*}}\right) )$ is a self-confirming equilibrium too. Hence we have the following *dogmatic equivalence principle*.

#### Proposition 3

*Let* $\left( a^{*},\mu ^{*}\right)$ *be a self-confirming equilibrium given* $\sigma ^{*}$. *If* $\sigma _{\mu ^{*}}\in \Sigma$*, then* $(a^{*},\delta \left( \sigma _{\mu ^{*}}\right) )$ *is a self-confirming equilibrium as well, with* $V\left( a^{*},\mu ^{*}\right) =V(a^{*},\delta \left( \sigma _{\mu ^{*}}\right) )$.

### Value and welfare

We now turn to a “welfare analysis,” that is, we compare equilibrium values with objective expected payoffs, including the maximum expected payoff that could be attained by the decision maker if she knew the true model $\sigma ^{*}$. We start with an important preliminary result: since we assume that consequences are observable, it follows that, for each action, observationally equivalent models yield the same objective expected payoff.^29^

#### Lemma 2

*Let* $\left( a,\sigma \right) \in A\times \Delta \left( S\right)$. *If model* $\sigma ^{\prime }\in \Delta \left( S\right)$ *is observationally equivalent to model* $\sigma$ *under* *a, then* $R\left( a,\sigma ^{\prime }\right) =R\left( a,\sigma \right)$.

This has a noteworthy consequence for self-confirming equilibrium values.

#### Proposition 4

*If* $\left( a^{*},\mu ^{*}\right)$ *is a self-confirming equilibrium given* $\sigma ^{*}$*, then*

$$\begin{aligned} V\left( a^{*},\mu ^{*}\right) =R\left( a^{*},\sigma ^{*}\right) . \end{aligned}$$

Thus, the value of any self-confirming equilibrium $\left( a^{*},\mu ^{*}\right)$ coincides with the true expected payoff of $a^{*}$, irrespective of the supporting belief $\mu ^{*}$. As a result, because of the data confirmation condition, the optimality condition (6) amounts to assuming that the “true value” of the self-confirming equilibrium action is higher than the subjective value, under the equilibrium belief, of all alternative actions. This interplay of objective and subjective features shows the substantial bite of the data confirmation condition.

Lemma 2 has interesting comparative welfare implications. For our welfare analysis, it is convenient to focus on actions that are part of some self-confirming equilibrium, thus neglecting the supporting confirmed beliefs.

#### Definition 2

Action $a^{*}$ is a *self-confirming (equilibrium) action* given $\sigma ^{*}$ if there exists a belief $\mu ^{*}\in \Delta \left( \Sigma \right)$ such that $\left( a^{*},\mu ^{*}\right)$ is a self-confirming equilibrium.

Since in this case $V\left( a^{*},\mu ^{*}\right) =R\left( a^{*},\sigma ^{*}\right) \le \sup _{a\in A}R\left( a,\sigma ^{*}\right)$ , the decision maker incurs a welfare loss

$$\begin{aligned} \ell \left( a^{*},\sigma ^{*}\right) =\sup _{a\in A}R\left( a,\sigma ^{*}\right) -R\left( a^{*},\sigma ^{*}\right) \ge 0 \end{aligned}$$

when she selects the self-confirming action $a^{*}$. In particular, $\ell \left( a^{*},\sigma ^{*}\right) =0$ if and only if $a^{*}$ is objectively optimal; the loss is caused by the decision maker’s ignorance, which makes it possible to assign positive subjective probability to (neighborhoods of) models different from the true one. Our next result shows that self-confirming equilibria with sharper basic subjective assessments yield higher welfare (lower loss). Formally, $\mu ^{*}$ is *absolutely continuous* with respect to (i.e., “sharper than”) $\nu ^{*}$ if, for every Borel set $B\subseteq \Sigma$, $\nu ^{*}\left( B\right) =0$ implies $\mu ^{*}\left( B\right) =0$ (equivalently, $\mu ^{*}\left( B\right) >0$ implies $\nu ^{*}\left( B\right) >0$). This means that $\mu ^{*}$ rules out more models than $\nu ^{*}$; in particular, if $\Sigma$ is finite, it means that supp$\mu ^{*}\subseteq ~$supp$\nu ^{*}$.

#### Proposition 5

*Let* $\left( a^{*},\mu ^{*}\right)$ *and* $\left( b^{*},\nu ^{*}\right)$ *be self-confirming equilibria such that* $\mu ^{*}$ *is absolutely continuous with respect to* $\nu ^{*}$*, then* $\ell \left( a^{*},\sigma ^{*}\right) \le \ell \left( b^{*},\sigma ^{*}\right)$.

Consider the self-confirming equilibria $\left( a^{*},\mu ^{*}\right)$ and $\left( b^{*},\nu ^{*}\right)$ such that (i) $a^{*}$ yields better identification than $b^{*}$ (i.e., ${\hat{\Sigma }} _{a^{*}}(\sigma ^{*})\subseteq {\hat{\Sigma }}_{b^{*}}(\sigma ^{*})$), and (ii) $\mu ^{*}$ and $\nu ^{*}$ do not rule out any model consistent with the statistical evidence given $a^{*}$ and $b^{*}$ respectively. Then, we obtain a special case of Proposition 5 and we can conclude that $\ell \left( a^{*},\sigma ^{*}\right) \le \ell \left( b^{*},\sigma ^{*}\right)$. The following result shows that we can dispense with condition (ii): Independently of their justifying confirmed beliefs, self-confirming actions with better identification properties exhibit lower losses.

#### Proposition 6

*Let* $a^{*}$ *and* $b^{*}$ *be self-confirming actions given* $\sigma ^{*}$. *If* ${\hat{\Sigma }}_{a^{*}}(\sigma ^{*})\subseteq {\hat{\Sigma }}_{b^{*}}(\sigma ^{*})$*, then* $\ell \left( a^{*},\sigma ^{*}\right) \le \ell \left( b^{*},\sigma ^{*}\right)$.

Propositions 5 and 6 are the only results in our analysis that depend on the one-person assumption in an essential way. In a multi-person game they hold only for the comparison of equilibria where the strategies of all players but one are the same and the focus is on the welfare of the only agent playing a different strategy.

The next related result shows that an action with the best identification properties —thus, optimal from a purely statistical viewpoint— is self-confirming only when objectively optimal. Truth is ancillary to the decision maker’s pursuit of her goals (and so of her happiness).

#### Proposition 7

*An action* $a\in A$ *such that* ${\hat{\Sigma }}_{a}(\sigma ^{*})\subseteq {\hat{\Sigma }}_{a^{\prime }}(\sigma ^{*})$ *for each* $a^{\prime }\in A$ *is self-confirming given* $\sigma ^{*}$ *if and only if it is objectively optimal.*

Under own-action independence of feedback about the state, ${\hat{\Sigma }} _{a}(\sigma ^{*})$ is independent of *a*. Therefore, Proposition 7 yields the following noteworthy implication.

#### Corollary 1

*Under own-action independence of feedback about the state, every self-confirming action is objectively optimal.*

#### Example 4

*(Monopoly: self-confirming equilibrium) *Under the assumptions of Example 1, certainty equivalence holds and the subjective best reply function of the monopolist is

$$\begin{aligned} B\left( \mu \right) =B\left( {\mathbb {E}}_{\mu }\left( \theta \right) \right) =\max \left\{ {\underline{a}},\frac{{\mathbb {E}}_{\mu }\left( \theta \right) -c }{2}\right\} \text {,} \end{aligned}$$

where $\theta$ parameterizes models according to the average intercept of the inverse demand function. Since any positive output is revealing (see Example 3), if the firm is pre-committed to a positive minimum output (${\underline{a}}>0$) own-action independence of feedback holds and the only self-confirming output is the objective best reply $\max \left\{ {\underline{a}},\left( \theta ^{*}-c\right) /2\right\}$. Next, suppose that ${\underline{a}}=0$, and furthermore ${\underline{\theta }}<c$ and $\theta ^{*}>c$. Then own-action independence of feedback does not hold and there are two self-confirming actions: (i) the fully revealing action $a^{*}=\left( \theta ^{*}-c\right) /2>0$ is the objective best reply, thus illustrating Proposition 7, and (ii) $b^{*}=0$ is justified by any “pessimistic” belief $\mu$ such that ${\mathbb {E}}_{\mu }\left( \theta \right) <c$, which is trivially consistent with long-run evidence because $b^{*}=0$ is fully *un-*revealing. The comparison of self-confirming actions $a^{*}$ and $b^{*}$ illustrates Proposition 6: indeed, $a^{*}$ is more revealing than $b^{*}$ and $\ell \left( a^{*},\theta ^{*}\right) =0<\left( \frac{\theta ^{*}-c}{2}\right) ^{2}=\ell \left( b^{*},\theta ^{*}\right)$.

The example prompts the following question. We mentioned in the Introduction that self-confirming equilibria are limit steady states of active learning processes, which we do not model explicitly here. Suppose that the monopolist believes it is optimal in the short run to produce 0, but deems it possible that the objective best reply is positive, i.e., that $\theta >c$ . Should she not experiment with a positive output? This depends on several elements: her subjective belief, her degree of patience (discount factor), and the amount of noise. If the subjective probability $\mu \left( \theta >c\right)$ is relatively small and price is noisy, it is dynamically optimal not to experiment even if the decision maker is moderately patient. In particular, noise is important: only repeated experimentation with positive output can provide reliable evidence, and this has a high subjective opportunity cost.^30^ In sum, the decision maker is not just a statistician: she is not interested in discovering the true model per se, unless the action (played in the long run) that allows the discovery is subjectively optimal.

In this first part we expressed and analyzed the self-confirming equilibrium concept in an abstract framework amenable to policy applications. This requires to allow for an infinite action space (e.g., to use calculus) and for an infinite state space, and to posit an objective probability model characterizing the data generating process. Technically, the latter calls for the use of standard Borel spaces. Many of the themes analyzed within the framework of the first part are illustrated in the second part by an application to monetary policy.

## Phillips curve exploitation model

We now illustrate our machinery in the context of a 1970’s U.S. policy debate about whether a trade-off between inflation and unemployment can be systematically exploited by a benevolent policy maker. We extend a formulation of Sargent (1999, 2008), who presents a self-confirming equilibrium in which a policy maker believes in a model asserting an exploitable trade-off between unemployment and inflation while the truth is that the trade-off is not exploitable.^31^

### Steady state model economies

We study a class $\Theta$ of model economies $\theta$ at a (stochastic) steady state. We assume that unemployment *u* and inflation $\pi$, beyond depending on the unknown $\theta$, are affected by random shocks *w* and $\varepsilon$ with zero mean, and by a monetary policy variable *a*. Specifically, unemployment and inflation outcomes $\left( u,\pi \right)$ are connected to the state of the economy $s=\left( w,\varepsilon ,\theta \right)$ and the government action *a* according to

11 $$\begin{aligned} u&=\theta _{0}+\theta _{1\varvec{\pi }}\pi +\theta _{1{\mathbf {a}}}a+\theta _{2}w, \end{aligned}$$

12 $$\begin{aligned} \pi&=a+\theta _{3}\varepsilon . \end{aligned}$$

The vector parameter $\theta =\left( \theta _{0},\theta _{1\varvec{\pi } },\theta _{1{\mathbf {a}}},\theta _{2},\theta _{3}\right) \in {\mathbb {R}}^{5}$, that is, the last component of the state vector, specifies the structural coefficients of an aggregate supply equation (11) and an inflation determination equation (12). Coefficients $\theta _{1\varvec{\pi }}$ and $\theta _{1{\mathbf {a}}}$ are slope responses of unemployment to actual and planned inflation,^32^ while the coefficients $\theta _{2}$ and $\theta _{3}$ quantify shock volatilities (see Sargent 2008, p. 18). Finally, the intercept $\theta _{0}$ is the baseline rate of unemployment that would (systematically) prevail at a zero planned inflation policy $a=0$.

Throughout the section we maintain the following assumption about structural coefficients.

#### Assumption 1

$$\theta _{0}>0$$, $$\theta _{1\varvec{\pi }}<0$$, $$\theta _{2}>0$$ and $$\theta _{3}>0$$.

In words, we posit a strictly positive baseline rate of unemployment, as well as strictly positive shock coefficients (nontrivial, possibly asymmetric, shocks thus affect both the inflation and the unemployment equations, their unknown values form the first component $\left( w,\varepsilon \right)$ of the state vector). Finally, we assume that —other things being equal— more inflation reduces unemployment.

The reduced form of each model economy is

13 $$\begin{aligned} u&=\theta _{0}+\left( \theta _{1\varvec{\pi }}+\theta _{1{\mathbf {a}}}\right) a+\theta _{1\varvec{\pi }}\theta _{3}\varepsilon +\theta _{2}w, \end{aligned}$$

14 $$\begin{aligned} \pi&=a+\theta _{3}\varepsilon . \end{aligned}$$

The coefficients of the reduced form are $\xi =\left( \theta _{0},\theta _{1 \varvec{\pi }}+\theta _{1{\mathbf {a}}},\theta _{1\varvec{\pi }}\theta _{3},\theta _{2},\theta _{3}\right) \in {\mathbb {R}}^{5}$. Since $\theta _{3}\not =0$ (Assumption 1), it is easy to check that different structural parameter vectors $\theta \in \Theta$ correspond to different reduced form parameter vectors $\xi$, that is, $\theta \not =\theta ^{\prime }$ implies $\xi \not =\xi ^{\prime }$.

We assume that only realized unemployment and inflation are observable by the monetary authority. Thus, the reduced form above will give us the feedback function $\left( u,\pi \right) =f\left( a,s\right)$of the previous sections. Specifically, rewriting (13) and (14) as

$$\begin{aligned} {\varvec{u}}\left( a,w,\varepsilon ,\theta \right)&=\theta _{0}+\left( \theta _{1\varvec{\pi }}+\theta _{1{\mathbf {a}}}\right) a+\theta _{1\mathbf { \pi }}\theta _{3}\varepsilon +\theta _{2}w\text {,} \\ \varvec{\pi }\left( a,w,\varepsilon ,\theta \right)&=a+\theta _{3}\varepsilon \end{aligned}$$

makes the dependence of observables $\left( u,\pi \right)$ on action *a* and (unobservable) realized states $\left( w,\varepsilon ,\theta \right)$ explicit, which allows us to study the present policy problem within our general framework. Formally, the message space $M={\mathbb {R}}^{2}$ now consists of unemployment/inflation pairs, and the feedback function is $f=\left( {\varvec{u}},\varvec{\pi }\right) :A\times \left( {\mathbb {R}} ^{2}\times \Theta \right) \rightarrow {\mathbb {R}}^{2}$.

The policy multiplier $\xi _{2}=\theta _{1\varvec{\pi }}+\theta _{1{\mathbf {a}} }=\theta _{1{\mathbf {a}}}-\left| \theta _{1\varvec{\pi }}\right|$ quantifies the impact of planned inflation on unemployment. It is the sum of the direct and indirect impact of planned inflation on unemployment quantified, respectively, by $\theta _{1{\mathbf {a}}}$ and $\theta _{1\mathbf { \pi }}$. There is a systematic trade-off between unemployment and inflation when the multiplier is strictly negative, that is, $\xi _{2}<0$. If so, the model economy is *Keynesian*; otherwise, it is *new-classical*. In the rest of the section we make the following hypothesis on the multiplier.

#### Assumption 2

$$\xi _{2}\le 0$$.

Thus, we assume that an increase in planned inflation never increases unemployment. A possible interpretation of the model is that $\theta _{1\mathbf { a}}/\left| \theta _{1\varvec{\pi }}\right|$ is the constant fraction of experienced/sophisticated agents in the economy who factor planned inflation into their expectations, and $\xi _{2}/\theta _{1\mathbf { \pi }}$ is the fraction of inexperienced/naive agents.

To sum up, the set of parameters is

$$\begin{aligned} \Theta =\left\{ \theta \in {\mathbb {R}}^{5}:\theta _{0}>0\text {, }\theta _{1 {\mathbf {a}}}\le -\theta _{1\varvec{\pi }}\text {, }\theta _{1\varvec{\pi }}<0 \text {, }\theta _{2}>0\text {, }\theta _{3}>0\right\} . \end{aligned}$$

To clarify our language, we note that we keep using “model” in the same sense as in the previous sections, that is, a probability measure over states, or a specific parameter value that determines such measure. Thus, a set of parameterized equations like ( 11)-(12) corresponds to a class of models. We will therefore refer subclasses of models satisfying some restrictions as “kinds”. With this, our analysis will pay special attention to the following two competing kinds of model economies.

#### Lucas-Sargent models

The first kind of model economy, based on Lucas (1972) and Sargent (1973), is

$$\begin{aligned} u&=\theta _{0}+\beta \left( \pi -a\right) +\theta _{2}w=\theta _{0}+\beta \theta _{3}\varepsilon +\theta _{2}w, \\ \pi&=a+\theta _{3}\varepsilon , \end{aligned}$$

where $\beta \equiv \theta _{1\varvec{\pi }}=-\theta _{1{\mathbf {a}}}$, and so $\theta =\left( \theta _{0},\beta ,-\beta ,\theta _{2},\theta _{3}\right)$ and $\xi =\left( \theta _{0},0,\beta \theta _{3},\theta _{2},\theta _{3}\right)$. In such new-classical models the policy multiplier $\xi _{2}$ is zero, and so the systematic part of inflation *a* has no effect on unemployment; only the unsystematic part $\theta _{3}\varepsilon$ does.

#### Samuelson-Solow models

A second kind of model economy, based on Samuelson and Solow (1960), is

$$\begin{aligned} u&=\theta _{0}+\theta _{1\varvec{\pi }}\pi +\theta _{2}w=\theta _{0}+\theta _{1\varvec{\pi }}a+\theta _{1\varvec{\pi }}\theta _{3}\varepsilon +\theta _{2}w, \\ \pi&=a+\theta _{3}\varepsilon , \end{aligned}$$

where $\theta _{1{\mathbf {a}}}=0$ and so $\theta =\left( \theta _{0},\theta _{1 \varvec{\pi }},0,\theta _{2},\theta _{3}\right)$ and $\xi =\left( \theta _{0},\theta _{1\varvec{\pi }},\theta _{1\varvec{\pi }}\theta _{3},\theta _{2},\theta _{3}\right)$. In such Keynesian models, the policy multiplier $\xi _{2}=\theta _{1\varvec{\pi }}$ is strictly negative: monetary policies affect, at steady state, unemployment rates.

### The policy problem: setup and identification

#### Setup

The monetary authority chooses policy *a*. As anticipated, the state space is the Cartesian product $S=W\times E\times \Theta$, which expresses that the monetary authority is uncertain about both shocks and permanent features of the economy, or models. The consequence space *C* consists of unemployment and inflation pairs $c=\left( u,\pi \right)$, so we set $C=U\times \Pi \subseteq {\mathbb {R}}^{2}$. The consequence function $\rho :A\times \left( W\times E\times \Theta \right) \rightarrow C$ is

$$\begin{aligned} \rho \left( a,w,\varepsilon ,\theta \right) =\left( {\varvec{u}}\left( a,w,\varepsilon ,\theta \right) ,\varvec{\pi }\left( a,w,\varepsilon ,\theta \right) \right) , \end{aligned}$$

which is the unemployment/inflation pair $\left( u,\pi \right)$ determined by policy *a* and state $\left( w,\varepsilon ,\theta \right)$, with matrix representation

15 $$\begin{aligned} \rho \left( a,w,\varepsilon ,\theta \right) =\left[ \begin{array}{c} \theta _{0} \\ 0 \end{array} \right] +a\left[ \begin{array}{c} \theta _{1\varvec{\pi }}+\theta _{1{\mathbf {a}}} \\ 1 \end{array} \right] +\left[ \begin{array}{cc} \theta _{2} &{} \theta _{1\varvec{\pi }}\theta _{3} \\ 0 &{} \theta _{3} \end{array} \right] \left[ \begin{array}{c} w \\ \varepsilon \end{array} \right] . \end{aligned}$$

#### Factorization

As anticipated, we assume that the messages received by the monetary authority are the policy outcomes. Hence, a message $m=\left( u,\pi \right)$ consists of an unemployment and inflation pair, and the feedback function

$$\begin{aligned} f=\rho =\left( {\varvec{u}},\varvec{\pi }\right) \end{aligned}$$

corresponds to the reduced form of the model economy. When the monetary authority chooses policy *a* and in the long run observes a distribution over $\left( u,\pi \right)$ pairs, it can partially infer the underlying stochastic model $\sigma$. For example, if $\sigma$ has finite support, the induced probability of outcome $\left( u,\pi \right)$ is^33^

16 $$\begin{aligned} {\hat{f}}_{a}\left( \sigma \right) \left( u,\pi \right) =\sigma \left( \left\{ \left( w,\varepsilon ,\theta \right) :\left( {\varvec{u}}\left( a,w,\varepsilon ,\theta \right) ,\varvec{\pi }\left( a,w,\varepsilon ,\theta \right) \right) =\left( u,\pi \right) \right\} \right) . \end{aligned}$$

The partially identified set ${\hat{\Sigma }}_{a}\left( \sigma \right)$ of stochastic models indistinguishable from $\sigma$ is the set of $\sigma ^{\prime }$ that induce the same joint distribution on unemployment/inflation outcomes given *a*.

At this point, it is convenient to add structure to this setup to provide a sharp characterization of the partially identified set corresponding to each policy *a* and model $\sigma$. Within a state $s=\left( w,\varepsilon ,\theta \right)$, the pair $\left( w,\varepsilon \right)$ represents random shocks and $\theta$ parameterizes a model economy. This suggests factorizing the probability models $\sigma \in \Sigma \subseteq \Delta \left( W\times E\times \Theta \right)$ as

17 $$\begin{aligned} \sigma =q\times \delta \left( \theta \right) , \end{aligned}$$

where the true marginal distribution of shocks $q\in \Delta \left( W\times E\right)$ is assumed to be *known* and $\delta \left( \theta \right) \in \Delta \left( \Theta \right)$ is a Dirac probability measure concentrated on a given economic model $\theta \in \Theta$, a permanent feature of the environment. We thus parameterize probability models with $\theta$ and write $\sigma _{\theta }$.

The simplifying assumption that, at a steady state, the distribution *q* of shocks is known is common in the rational expectations literature since Lucas and Prescott (1971) and Lucas (1972). The resulting factorization (17) has two modeling consequences: (i) it establishes a one-to-one correspondence between model economies and probability models (in particular, a true economic model $\theta ^{*}$ corresponds to a true probability model $\sigma _{\theta ^{*}}$); (ii) since *q* is known, it allows us to identify $\Sigma$ with $\Theta$ via the relation

$$\begin{aligned} \Sigma =\left\{ q\times \delta \left( \theta \right) \in \Delta \left( S\right) :\theta \in \Theta \right\} , \end{aligned}$$

and so to define the prior $\mu$ on $\Theta$.^34^

A first dividend of the factorization is that the objective function (2) takes the simpler form

18 $$\begin{aligned} V\left( a,\mu \right) =\int _{\Theta }\left( \int _{W\times E}r\left( a,w,\varepsilon ,\theta \right) dq\left( w,\varepsilon \right) \right) d\mu \left( \theta \right) , \end{aligned}$$

where $r\left( a,w,\varepsilon ,\theta \right) =v\left( \rho \left( a,w,\varepsilon ,\theta \right) \right)$ is the utility of outcome/message $\left( u,\pi \right) =\rho \left( a,w,\varepsilon ,\theta \right)$.

In the rest of the section we maintain the following assumption on the known shock distributions.^35^

##### Assumption 3

$${\mathbb {E}}_{q}\left( \varvec{\varepsilon } \right) ={\mathbb {E}}_{q}\left( {\mathbf {w}}\right) ={\mathbb {E}}_{q}\left( \varvec{\varepsilon w}\right) =0$$ and $${\mathbb {E}}_{q}\left( \varvec{\varepsilon }^{2}\right) ={\mathbb {E}}_{q}\left( {\mathbf {w}}^{2}\right) =1$$.

In words, shocks are uncorrelated and normalized.

#### Identification

In this “factorized” setup, we can shift our focus from observationally equivalent probability models $\sigma$ to observationally equivalent model economies $\theta$. The partially identified set becomes:

$$\begin{aligned} \forall \theta \in \Theta ,\ \ {\hat{\Sigma }}_{a}\left( \theta \right) =\left\{ \theta ^{\prime }\in \Theta :{\hat{f}}_{a}\left( \sigma _{\theta ^{\prime }}\right) ={\hat{f}}_{a}\left( \sigma _{\theta }\right) \right\} . \end{aligned}$$

With this, a sharp identification result holds.

##### Proposition 8

*The partial identification correspondence* $\hat{ \Sigma }_{a}:\Theta \rightarrow 2^{\Theta }$ *is*

19 $$\begin{aligned} {\hat{\Sigma }}_{a}\left( \theta \right) =\left\{ \theta ^{\prime }\in \Theta :\theta _{0}^{\prime }+\theta _{1{\mathbf {a}}}^{\prime }a=\theta _{0}+\theta _{1{\mathbf {a}}}a,\, \theta _{1\varvec{\pi }}^{\prime }=\theta _{1\varvec{\pi } },\, \theta _{2}^{\prime }=\theta _{2},\, \theta _{3}^{\prime }=\theta _{3}\right\} . \end{aligned}$$

Given the true model $\theta$, the shock coefficients $\theta _{2}$ and $\theta _{3}$ are thus identified, along with the slope $\theta _{1\varvec{\pi }}$ of the Phillips curve, independently of the chosen policy *a*. As we discuss below, the intercept of the curve is also identified, but it depends on the maintained policy *a* through the unidentified parameter $\theta _{1 {\mathbf {a}}}$. This important identification result is made possible by some moment conditions, formally spelled out in the proof. We can, however, heuristically describe them via the bivariate random variable $\left( {\varvec{u}}_{a},\varvec{\pi }_{a}\right) :W\times E\times \Theta \rightarrow U\times \Pi$ that, for a given policy *a*, represents the unemployment and inflation rates determined by the state $\left( w,\varepsilon ,\theta \right)$.^36^ The monetary authority infers the following moments from the long-run distribution of outcomes:

- $${\mathbb {E}}_{\theta }\left( {\varvec{u}}_{a}\right) =\theta _{0}+\left( \theta _{1\varvec{\pi }}+\theta _{1{\mathbf {a}}}\right) a$$,
- $${\mathbb {E}}_{\theta }\left( \varvec{\pi }_{a}\right) =a$$,
- $$\mathrm {Var}_{\theta }\left( {\varvec{u}}_{a}\right) =\theta _{1 \varvec{\pi }}^{2}\theta _{3}^{2}+\theta _{2}^{2}$$,
- $$\mathrm {Var}_{\theta }\left( \varvec{\pi }_{a}\right) =\theta _{3}^{2}$$,
- $$\mathrm {Cov}_{\theta }\left( {\varvec{u}}_{a},\varvec{\pi } _{a}\right) =\theta _{1\varvec{\pi }}\theta _{3}^{2}$$.

Therefore,

20 $$\begin{aligned} \theta _{1\varvec{\pi }}=\frac{\mathrm {Cov}_{\theta }\left( {\varvec{u}} _{a},\varvec{\pi }_{a}\right) }{\mathrm {Var}_{\theta }\left( \varvec{ \pi }_{a}\right) } \end{aligned}$$

is the beta coefficient of the Phillips regression of unemployment on inflation,^37^

$$\begin{aligned} \theta _{2}^{2}=\left( 1-\mathrm {Corr}_{\theta }^{2}\left( {\varvec{u}} _{a},\varvec{\pi }_{a}\right) \right) \mathrm {Var}_{\theta }\left( {\varvec{u}}_{a}\right) \end{aligned}$$

is the residual variance of ${\varvec{u}}_{a}$ (unexplained by the regression), and $\theta _{3}$ is the standard deviation of inflation.

Finally, though the two structural coefficients $\theta _{0}$ and $\theta _{1 {\mathbf {a}}}$ remain unidentified even in the long-run, they satisfy

21 $$\begin{aligned} \theta _{0}+\theta _{1{\mathbf {a}}}a={\mathbb {E}}_{\theta }\left( {\varvec{u}} _{a}\right) -\frac{\mathrm {Cov}_{\theta }\left( {\varvec{u}}_{a}, \varvec{\pi }_{a}\right) }{\mathrm {Var}_{\theta }\left( \varvec{\pi } _{a}\right) }{\mathbb {E}}_{\theta }\left( \varvec{\pi }_{a}\right) , \end{aligned}$$

where the right side is the alpha coefficient of the Phillips regression. In the long-run, the alpha coefficient is observed by the monetary authority, but what is observed depends on the policy *a* that the authority chooses.

#### Estimated model economy

As an approximation of a situation in which the dataset is large and the sample variance is small, we take the idealized perspective of a monetary authority (or its econometrician) who can rely on an infinite dataset and therefore can perfectly estimate the identifiable parameters by observing some moments of the true distribution as specified in Proposition 8.

The moments that identify the three coefficients $\theta _{1\varvec{\pi }}$, $\theta _{2}$, and $\theta _{3}$ do not depend on the chosen policy *a*, but only on the true model $\theta$. To emphasize this key feature, we denote by ${\hat{\beta }}$ the beta regression coefficient that identifies $\theta _{1 \varvec{\pi }}$,^38^ by ${\hat{\sigma }}_{{\varvec{u}} \mid \varvec{\pi }}$ the residual standard deviation that identifies $\theta _{2}$, and by ${\hat{\sigma }}_{\varvec{\pi }}$ the standard deviation of inflation that identifies $\theta _{3}$. In contrast, the alpha regression coefficient that identifies the sum $\theta _{0}+\theta _{1{\mathbf {a}}}a$ depends on policy *a*; we denote it by ${\hat{\alpha }}\left( a\right)$.

With this, we can write

$$\begin{aligned} {\hat{\Sigma }}_{a}\left( \theta \right) =\left\{ \theta ^{\prime }\in \Theta :\theta _{0}^{\prime }+\theta _{1{\mathbf {a}}}^{\prime }a={\hat{\alpha }}\left( a\right) ,\, \theta _{1\varvec{\pi }}^{\prime }={\hat{\beta }},\, \theta _{2}^{\prime }={\hat{\sigma }}_{{\varvec{u}}\mid \varvec{\pi }},\, \theta _{3}^{\prime }={\hat{\sigma }}_{\varvec{\pi }}\right\} . \end{aligned}$$

As a result, the long-run estimated version of the model economy (11, 12) that the monetary authority considers is

22 $$\begin{aligned} u&={\hat{\alpha }}\left( a\right) +{\hat{\beta }}\pi +{\hat{\sigma }}_{{\varvec{u}} \mid \varvec{\pi }}w, \end{aligned}$$

23 $$\begin{aligned} \pi&=a+{\hat{\sigma }}_{\varvec{\pi }}\varepsilon , \end{aligned}$$

24 $$\begin{aligned} {\hat{\alpha }}\left( a\right)&=\theta _{0}+\theta _{1{\mathbf {a}}}a. \end{aligned}$$

In particular, (22) is the estimated aggregate supply equation and (23) is the estimated inflation equation. The intercept of the former equation depends on policy *a* via eq. (24), which only partly identifies the two coefficients $\theta _{0}$ and $\theta _{1 {\mathbf {a}}}$. In turn, this makes the policy multiplier $\xi _{2}={\hat{\beta }} +\theta _{1{\mathbf {a}}}$ unidentified. We will momentarily address this key partial identification issue.

#### Partial identification line

The monetary authority cannot identify —even in the long-run— the two structural coefficients $\theta _{0}$ and $\theta _{1{\mathbf {a}}}$. The former is the average unemployment at zero planned inflation, $\theta _{0}= {\mathbb {E}}_{\theta }\left( {\varvec{u}}_{0}\right)$; the latter is the “direct” impact of policy on unemployment.

The parameter space of the estimated model economy (22, 24) reduces to $\Theta ={\tilde{\Theta }}\times \{({\hat{\beta }},\, \hat{ \sigma }_{{\varvec{u}}\mid \varvec{\pi }},\, {\hat{\sigma }}_{\varvec{\pi } })\}$, where ${\tilde{\Theta }}={\mathbb {R}}_{++}\times (-\infty ,-{\hat{\beta }}]$ is the collection of all possible values $(\theta _{0},\theta _{1{\mathbf {a}} })$ of the two remaining unidentified coefficients and $\{({\hat{\beta }},\, {\hat{\sigma }}_{{\varvec{u}}\mid \varvec{\pi }},\, {\hat{\sigma }}_{\varvec{\pi }})\}$ is the singleton containing the identified vector $\left( \theta _{1 \varvec{\pi }},\theta _{2},\theta _{3}\right)$. To ease notation, in what follows we will consider directly ${\tilde{\Theta }}$ as the parameter space. As a result, the parameter space is now a subset of the plane. By (19), the partial identification correspondence $\hat{ \Sigma }_{a}:{\tilde{\Theta }}\rightarrow 2^{{\tilde{\Theta }}}$ becomes

25 $$\begin{aligned} {\hat{\Sigma }}_{a}\left( \theta \right) =\left\{ \left( \theta _{0}^{\prime },\theta _{1{\mathbf {a}}}^{\prime }\right) \in {\tilde{\Theta }}:\theta _{0}^{\prime }=-\theta _{1{\mathbf {a}}}^{\prime }a+\theta _{0}+\theta _{1 {\mathbf {a}}}a\right\} \text {.} \end{aligned}$$

In words, ${\hat{\Sigma }}_{a}\left( \theta \right)$ is a straight line in the plane, with slope $-a$ and intercept $\theta _{0}+\theta _{1{\mathbf {a}}}a$ (determined by the policy *a* and by the true economic model $\theta$). We thus have a partial identification line that defines a linear relationship between the two unidentified coefficients, given the true model. In other words, partial identification is unidimensional.

Given true model $\theta =\left( \theta _{0},\theta _{1{\mathbf {a}}}\right)$, the collection $\{ {\hat{\Sigma }}_{a}\left( \theta \right) :a\in A\}$ of partial identification lines is the family of all straight lines in the plane that pass through the true model $\left( \theta _{0},\theta _{1\mathbf { a}}\right)$ and have slope $-1/a$. In each such line there is a unique Lucas-Sargent model, characterized by $\theta _{1{\mathbf {a}}}^{\prime }=-\hat{ \beta }$, as well as a unique Samuelson-Solow model, characterized by $\theta _{1{\mathbf {a}}}^{\prime }=0$. In other words, partial identification lines feature a unique specimen of each kind of models.

**Fig. 1:**
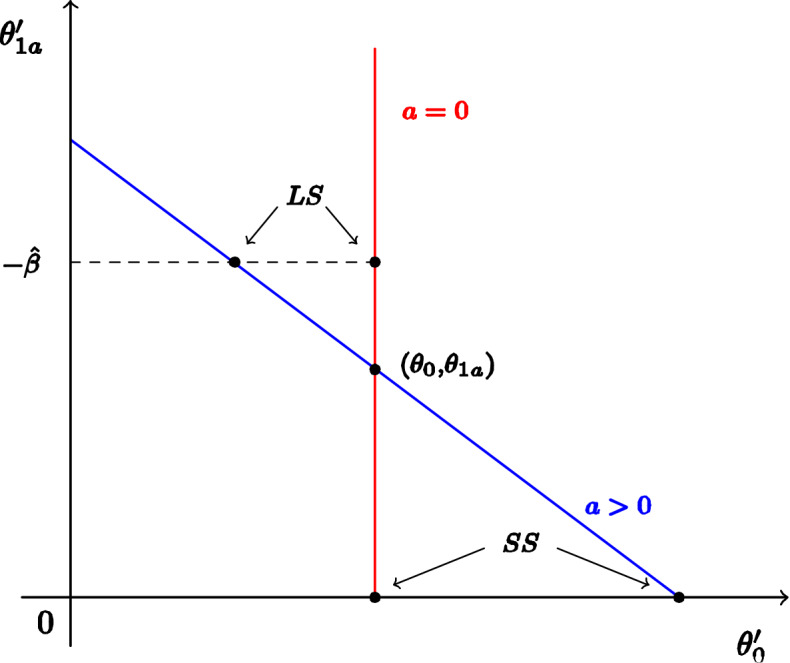
Partial identification line

Figure 1 illustrates the previous analysis. In particular, LS stands for Lucas-Sargent model and SS for Samuelson-Solow model, while the red (resp., blue) line is the partial identification line that correspond to policy $a=0$ (resp., $a>0$).

### The policy problem: value, equilibria and welfare

#### Value and equilibrium

As much of the literature, we assume a quadratic von Neumann-Morgenstern utility function $v:C\rightarrow {\mathbb {R}}$ given by $v\left( u,\pi \right) =-u^{2}-\pi ^{2}$, so that the reward function $r:A\times S\rightarrow {\mathbb {R}}$ becomes:

$$\begin{aligned} r\left( a,w,\varepsilon ,\theta \right) =-{\varvec{u}}^{2}\left( a,w,\varepsilon ,\theta \right) -\varvec{\pi }^{2}\left( a,w,\varepsilon ,\theta \right) . \end{aligned}$$

The linear model economy and quadratic utility together form a classic linear quadratic policy framework.

##### Lemma 3

*For every* $\left( \theta ,a\right) \in {\tilde{\Theta }} \times A$*, we have* $R\left( a,\theta \right) =v\left( {\mathbb {E}}_{\theta }\left( {\varvec{u}}_{a}\right) \text {, }{\mathbb {E}}_{\theta }\left( \varvec{\pi }_{a}\right) \right) +const$.

The linear quadratic framework thus allows us to express the expected reward as the utility of expectations. As a result, the objective function (18) becomes

26 $$\begin{aligned} V\left( a,\mu \right) =\int _{{\tilde{\Theta }}}v\left( {\mathbb {E}}_{\theta }\left( {\varvec{u}}_{a}\right) ),{\mathbb {E}}_{\theta }\left( \varvec{ \pi }_{a}\right) \right) d\mu \left( \theta \right) +const. \end{aligned}$$

As for self-confirming equilibria, we begin with a piece of notation: throughout the rest of this section we fix a true model economy $\theta ^{*}$ (*rather than *$\theta$) in ${\tilde{\Theta }}$, while $\theta$ (*rather than* $\theta ^{\prime }$) denotes a generic element of ${\tilde{\Theta }}$. With this notation, the partial identification line is

$$\begin{aligned} {\hat{\Sigma }}_{a}\left( \theta ^{*}\right) =\left\{ \left( \theta _{0},\theta _{1{\mathbf {a}}}\right) \in {\tilde{\Theta }}:\theta _{0}=\theta _{0}^{*}+\left( \theta _{1{\mathbf {a}}}^{*}-\theta _{1{\mathbf {a}} }\right) a\right\} . \end{aligned}$$

Hence, a policy and belief pair $\left( a^{*},\mu ^{*}\right) \in A\times \Delta ({\tilde{\Theta }})$ is self-confirming if and only if

$$\begin{aligned} a^{*}\in \arg \max _{a\in A}V\left( a,\mu ^{*}\right) \end{aligned}$$

and

$$\begin{aligned} \mu ^{*}\left( {\hat{\Sigma }}_{a^{*}}\left( \theta ^{*}\right) \right) =1. \end{aligned}$$

Next we characterize self-confirming equilibria of the estimated model economy (22, 23, 24). In both equilibrium conditions, the true multiplier $\xi _{2}^{*}={\hat{\beta }}^{*}+\theta _{1\mathbf {a }}^{*}$ and its conjectured value ${\mathbb {E}}_{\mu ^{*}}(\xi _{2})= {\hat{\beta }}^{*}+{\mathbb {E}}_{\mu ^{*}}\left( \theta _{1{\mathbf {a}} }\right)$ play a key role.^39^

##### Proposition 9

*A policy and belief pair* $\left( a^{*},\mu ^{*}\right) \in A\times \Delta ({\tilde{\Theta }})$ *is a self-confirming equilibrium given* $\theta ^{*}$ *if and only if*

27 $$\begin{aligned} a^{*}=-\frac{\theta _{0}^{*}\left( {\hat{\beta }}^{*}+{\mathbb {E}} _{\mu ^{*}}\left( \theta _{1{\mathbf {a}}}\right) \right) }{1+\left( \hat{ \beta }^{*}+\theta _{1{\mathbf {a}}}^{*}\right) \left( {\hat{\beta }}^{*}+{\mathbb {E}}_{\mu ^{*}}\left( \theta _{1{\mathbf {a}}}\right) \right) } \end{aligned}$$

*and*

28 $$\begin{aligned} \mu ^{*}\left( \left\{ \left( \theta _{0},\theta _{1{\mathbf {a}}}\right) \in {\tilde{\Theta }}:\theta _{0}=\theta _{0}^{*}-\frac{\theta _{0}^{*}\left( {\hat{\beta }}^{*}+{\mathbb {E}}_{\mu ^{*}}\left( \theta _{1 {\mathbf {a}}}\right) \right) }{1+\left( {\hat{\beta }}^{*}+\theta _{1\mathbf {a }}^{*}\right) \left( {\hat{\beta }}^{*}+{\mathbb {E}}_{\mu ^{*}}\left( \theta _{1{\mathbf {a}}}\right) \right) }\left( \theta _{1{\mathbf {a}}}^{*}-\theta _{1{\mathbf {a}}}\right) \right\} \right) =1. \end{aligned}$$

The result can be heuristically derived in the special case of *dogmatic beliefs*, when $\mu ^{*}$ is concentrated on a single parameter vector ${\bar{\theta }}=\left( {\bar{\theta }}_{0},{\bar{\theta }}_{1{\mathbf {a}} }\right) \in {\tilde{\Theta }}$, that is, $\mu ^{*}=\delta \left( \bar{ \theta }\right)$. By (26), up to a constant the monetary authority’s value function is

$$\begin{aligned} V\left( a,\mu ^{*}\right) =-{\mathbb {E}}_{{\bar{\theta }}}^{2}\left( {\varvec{u}}_{a}\right) -{\mathbb {E}}_{{\bar{\theta }}}^{2}\left( \varvec{ \pi }_{a}\right) . \end{aligned}$$

The conjectured multiplier is ${\bar{\xi }}_{2}={\hat{\beta }}^{*}+{\bar{\theta }} _{1{\mathbf {a}}}$. For instance, a new-classical authority that believes that there is no systematically exploitable trade-off between inflation and unemployment assumes ${\bar{\theta }}_{1{\mathbf {a}}}=-{\hat{\beta }}^{*}$ (and so the conjectured multiplier is zero). In contrast, a Keynesian authority that believes in a trade-off may assume, for instance, ${\bar{\theta }}_{1 {\mathbf {a}}}=0$ (the conjectured multiplier is then ${\hat{\beta }}^{*}$, and so strictly negative).

Based on the estimated model economy (22, 23, 24), a dogmatic authority conjectures that, according to the chosen policy *a*, the expected values of inflation and unemployment are constrained by the equation

$$\begin{aligned} {\mathbb {E}}_{{\bar{\theta }}}\left( {\varvec{u}}_{a}\right) ={\bar{\theta }} _{0}+\left( {\bar{\theta }}_{1{\mathbf {a}}}+{\hat{\beta }}^{*}\right) {\mathbb {E}} _{{\bar{\theta }}}\left( \varvec{\pi }_{a}\right) . \end{aligned}$$

This conjectured constraint is the version of the estimated aggregate supply equation (22) that the authority expects to face systematically given its dogmatic belief. So the authority’s decision problem is

$$\begin{aligned}&\min _{a\in A}\, {\mathbb {E}}_{{\bar{\theta }}}^{2}\left( {\varvec{u}} _{a}\right) +{\mathbb {E}}_{{\bar{\theta }}}^{2}\left( \varvec{\pi } _{a}\right) ,\\&{{\mathrm{sub}}}\, {\mathbb {E}}_{{\bar{\theta }}}\left( {\varvec{u}}_{a}\right) = {\bar{\theta }}_{0}+\left( {\bar{\theta }}_{1{\mathbf {a}}}+{\hat{\beta }}^{*}\right) {\mathbb {E}}_{{\bar{\theta }}}\left( \varvec{\pi }_{a}\right) . \end{aligned}$$

With this, the Lagrangian is

$$\begin{aligned} {\mathbb {E}}_{{\bar{\theta }}}^{2}\left( {\varvec{u}}_{a}\right) +{\mathbb {E}}_{ {\bar{\theta }}}^{2}\left( \varvec{\pi }_{a}\right) +\lambda \left( \mathbb { E}_{{\bar{\theta }}}\left( {\varvec{u}}_{a}\right) -\left( {\bar{\theta }} _{0}+\left( {\bar{\theta }}_{1{\mathbf {a}}}+{\hat{\beta }}^{*}\right) {\mathbb {E}} _{{\bar{\theta }}}\left( \varvec{\pi }_{a}\right) \right) \right) \end{aligned}$$

and the first-order conditions are

$$\begin{aligned} 2{\mathbb {E}}_{{\bar{\theta }}}\left( {\varvec{u}}_{a}\right) =-\lambda \quad \quad 2{\mathbb {E}}_{{\bar{\theta }}}\left( \varvec{\pi }_{a}\right) =\lambda \left( {\bar{\theta }}_{1{\mathbf {a}}}+{\hat{\beta }}^{*}\right) \quad \quad {\mathbb {E}}_{{\bar{\theta }}}\left( {\varvec{u}}_{a}\right) ={\bar{\theta }} _{0}+\left( {\bar{\theta }}_{1{\mathbf {a}}}+{\hat{\beta }}^{*}\right) {\mathbb {E}} _{{\bar{\theta }}}\left( \varvec{\pi }_{a}\right) . \end{aligned}$$

By solving them we get

$$\begin{aligned} {\mathbb {E}}_{{\bar{\theta }}}\left( \varvec{\pi }_{a}\right) =B\left( \bar{ \theta }\right) \equiv -\frac{{\bar{\theta }}_{0}\left( {\hat{\beta }}^{*}+\bar{ \theta }_{1{\mathbf {a}}}\right) }{1+\left( {\hat{\beta }}^{*}+{\bar{\theta }}_{1 {\mathbf {a}}}\right) ^{2}}. \end{aligned}$$

Since ${\mathbb {E}}_{{\bar{\theta }}}\left( \varvec{\pi }_{a}\right) =a$, the monetary authority’s best reply is thus the policy $a=B\left( {\bar{\theta }} \right)$. As a result, a policy and belief pair $\left( a^{*},\delta \left( {\bar{\theta }}\right) \right)$ is a self-confirming equilibrium if and only if

29 $$\begin{aligned} \begin{array}{ll} a^{*}=B\left( {\bar{\theta }}\right)&\quad (\text {subjective best reply}) \end{array} \end{aligned}$$

and

30 $$\begin{aligned} \begin{array}{ll} {\bar{\theta }}_{0}=\theta _{0}^{*}+\left( \theta _{1{\mathbf {a}}}^{*}- {\bar{\theta }}_{1{\mathbf {a}}}\right) a^{*}&\quad (\text {confirmed beliefs}). \end{array} \end{aligned}$$

Simple algebra shows that this is the case if and only if

31 $$\begin{aligned} a^{*}=-\frac{\theta _{0}^{*}\left( {\hat{\beta }}^{*}+{\bar{\theta }} _{1{\mathbf {a}}}\right) }{1+\left( {\hat{\beta }}^{*}+\theta _{1{\mathbf {a}} }^{*}\right) \left( {\hat{\beta }}^{*}+{\bar{\theta }}_{1{\mathbf {a}} }\right) } \end{aligned}$$

and

32 $$\begin{aligned} {\bar{\theta }}_{0}=\theta _{0}^{*}-\frac{\theta _{0}^{*}\left( \hat{ \beta }^{*}+{\bar{\theta }}_{1{\mathbf {a}}}\right) }{1+\left( {\hat{\beta }} ^{*}+\theta _{1{\mathbf {a}}}^{*}\right) \left( {\hat{\beta }}^{*}+ {\bar{\theta }}_{1{\mathbf {a}}}\right) }\left( \theta _{1{\mathbf {a}}}^{*}-\bar{ \theta }_{1{\mathbf {a}}}\right) , \end{aligned}$$

which are the equilibrium relations (27) and (28) in the case of dogmatic beliefs.^40^

**Fig. 2:**
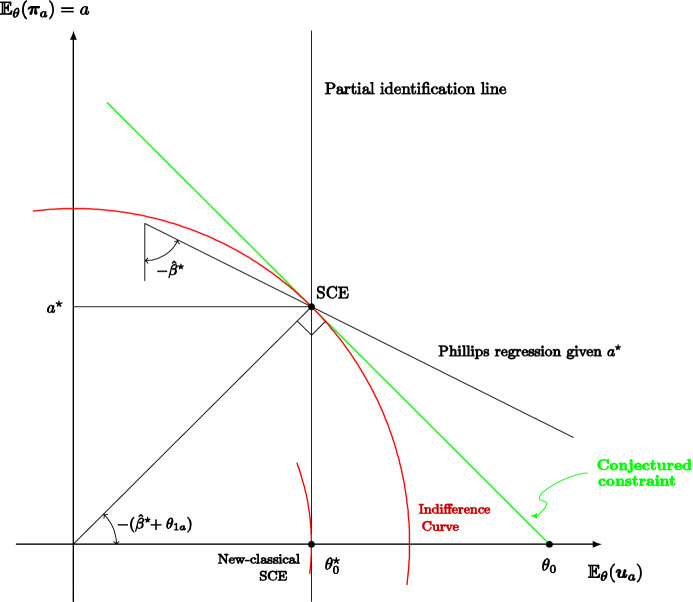
Self-confirming equilibrium in a new-classical world

Figure 2 illustrates the previous heuristic argument when the true model is of Lucas-Sargent kind, so that $\theta _{0}^{*}$ is the natural rate of unemployment and $\theta _{1{\mathbf {a}}}^{*}=-\hat{ \beta }^{*}$ (and so the true policy multiplier $\xi _{2}^{*}$ is zero). Under this true model, policy *a* induces average unemployment ${\mathbb {E}}_{\theta ^{*}}\left( {\varvec{u}}_{a}\right) =\theta _{0}^{*}$ and average inflation ${\mathbb {E}}_{\theta ^{*}}\left( \varvec{\pi }_{a}\right) =a$. But a monetary authority with dogmatic belief $\delta \left( {\bar{\theta }}\right)$ expects to observe the pair of long-run averages $\left( {\mathbb {E}}_{{\bar{\theta }}}\left( {\varvec{u}} _{a}\right) ,a\right)$. This dogmatic belief is confirmed, and so condition (30) is satisfied, if ${\mathbb {E}}_{{\bar{\theta }}}\left( {\varvec{u}}_{a}\right) =\theta _{0}^{*}$, that is, if the pair of average unemployment and average inflation lies on the vertical partial identification line with abscissa $\theta _{0}^{*}$. The subjective best reply condition (29) is represented by the tangency between the (red) indifference curve and the (green) conjectured constraint, according to which an increase $\Delta a$ in average inflation yields a $- {\bar{\xi }}_{2}\Delta a$ decrease in average unemployment, where ${\bar{\xi }} _{2}={\hat{\beta }}^{*}+{\bar{\theta }}_{1{\mathbf {a}}}$ is the conjectured multiplier.

**Fig. 3:**
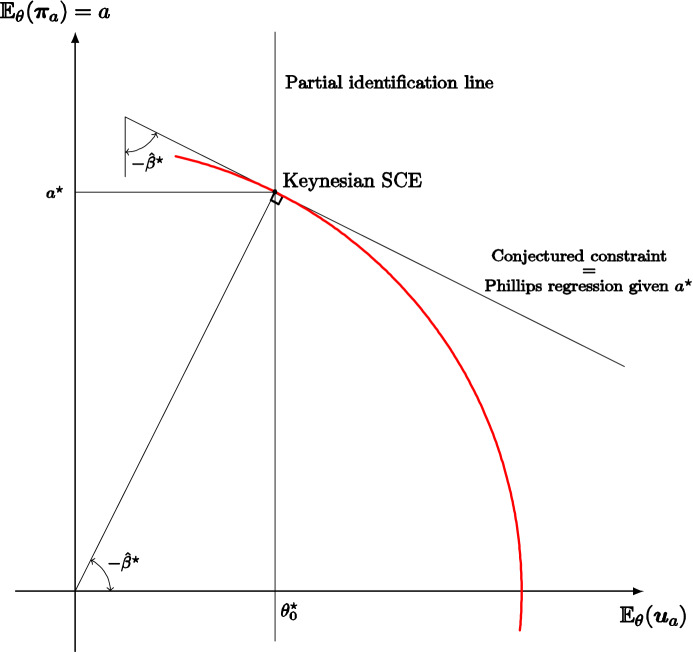
Equilibrium with “orthodox” Keynesian beliefs

When the dogmatic belief is such that ${\bar{\theta }}_{1{\mathbf {a}}}=0$ so that ${\bar{\xi }}_{2}={\hat{\beta }}^{*}$ becomes the conjectured multiplier, the monetary authority is “orthodox” Keynesian. See Fig. 3.

The conjectured constraint is ${\mathbb {E}}_{{\bar{\theta }}}\left( {\varvec{u}} _{a}\right) ={\bar{\theta }}_{0}+{\hat{\beta }}^{*}{\mathbb {E}}_{{\bar{\theta }} }\left( \varvec{\pi }_{a}\right)$. Its slope is the beta coefficient of the Phillips regression, which represents the trade-off between inflation and unemployment that the Keynesian authority believes to be systematically exploitable.

#### Policy activism and welfare

To complete our equilibrium analysis we need to compare the self-confirming equilibrium action with the objectively optimal one and to compute the resulting welfare loss.

To this end we need to consider the estimated policy multiplier $\xi _{2}= {\hat{\beta }}+\theta _{1{\mathbf {a}}}$. The authority underestimates the multiplier when ${\mathbb {E}}_{\mu ^{*}}(\xi _{2})>\xi _{2}^{*}$ and overestimates it when ${\mathbb {E}}_{\mu ^{*}}(\xi _{2})<\xi _{2}^{*}$.^41^ In structural terms, ${\mathbb {E}}_{\mu ^{*}}(\xi _{2})\gtrless \xi _{2}^{*}$ if and only if ${\mathbb {E}}_{\mu ^{*}}\left( \theta _{1 {\mathbf {a}}}\right) \gtrless \theta _{1{\mathbf {a}}}^{*}$. For instance, when $\theta _{1{\mathbf {a}}}^{*}$ and ${\mathbb {E}}_{\mu ^{*}}\left( \theta _{1{\mathbf {a}}}\right)$ are positive this means that the multiplier is under/overestimated if and only if the direct impact of planned inflation on unemployment is over/underestimated.

The objectively optimal policy is

33 $$\begin{aligned} a^{o}=-\frac{\theta _{0}^{*}\left( {\hat{\beta }}^{*}+\theta _{1\mathbf { a}}^{*}\right) }{1+\left( {\hat{\beta }}^{*}+\theta _{1{\mathbf {a}} }^{*}\right) ^{2}}. \end{aligned}$$

It is immediate to see that $a^{*}=a^{o}$ if and only if ${\mathbb {E}} _{\mu ^{*}}\left( \theta _{1{\mathbf {a}}}\right) =\theta _{1{\mathbf {a}} }^{*}$, (and so ${\mathbb {E}}_{\mu ^{*}}(\xi _{2})=\xi _{2}^{*}$). The equilibrium action is objectively optimal when the monetary authority has a correct expected value of the estimated policy multiplier $\xi _{2}$. More generally, next we show that policy hyperactivism characterizes authorities that overestimate the policy multiplier, while hypoactivism characterizes authorities that underestimate it.^42^

##### Proposition 10

*Given a true model* $\theta ^{*}$*, for every self-confirming equilibrium* $\left( a^{*},\mu ^{*}\right)$*,*

(i) ${\mathbb {E}}_{\mu ^{*}}\left( \theta _{1{\mathbf {a}}}\right) <\theta _{1{\mathbf {a}}}^{*}$ *if and only if policy* $a^{*}$ *is hyperactive, i.e.,* $a^{*}>a^{o}$*;*
(ii) ${\mathbb {E}}_{\mu ^{*}}\left( \theta _{1{\mathbf {a}}}\right) =\theta _{1{\mathbf {a}}}^{*}$ *if and only if policy* $a^{*}$ *is objectively optimal, i.e.,* $a^{*}=a^{o}$*;*
(iii) $\theta _{1{\mathbf {a}}}^{*}<{\mathbb {E}}_{\mu ^{*}}\left( \theta _{1{\mathbf {a}}}\right) <-{\hat{\beta }}^{*}$ *if and only if policy* $a^{*}$ *is hypoactive, i.e.,* $0<a^{*}<a^{o}$*;*
(iv) ${\mathbb {E}}_{\mu ^{*}}\left( \theta _{1{\mathbf {a}}}\right) =- {\hat{\beta }}^{*}$ *if and only if policy* $a^{*}$ *is zero-target-inflation, i.e.,* $a^{*}=0$.

For the monetary authority, both kinds of deviations from objective optimality, hyperactivism and hypoactivism, cause the same welfare loss. Indeed:

##### Proposition 11

*The welfare loss is* $\ell \left( a^{*},\theta ^{*}\right) =(1+({\hat{\beta }}^{*}+\theta _{1{\mathbf {a}}}^{*})^{2})\left( a^{*}-a^{o}\right) ^{2}$.

In the next section we will illustrate this result with a few examples.

### Policy dogmatism and its welfare consequences

#### Equilibria

Assume that the monetary authority has dogmatic equilibrium beliefs $\mu ^{*}=\delta \left( {\bar{\theta }}\right)$. A pair $\left( a^{*},\delta \left( {\bar{\theta }}\right) \right) \in A\times \Delta (\tilde{\Theta })$ is self-confirming if and only if it satisfies relations (31) and (32). Two special cases are noteworthy.

**New-classical authority** Suppose the monetary authority believes that the policy multiplier is zero, i.e., ${\bar{\theta }}_{1{\mathbf {a}}}=-{\bar{\theta }}_{1\varvec{\pi }}$. Since in equilibrium $\theta _{1\varvec{\pi }}$ is identified by the slope of the Phillips regression, we have ${\bar{\theta }}_{1{\mathbf {a}}}=-{\hat{\beta }}^{*}$. Here the conjectured constraint is vertical at the baseline unemployment rate $\theta _{0}^{*}$: the new-classical authority does not believe in any systematically exploitable trade-off between inflation and unemployment. A zero-target-inflation equilibrium policy results (Proposition 10-(iv)).

**Keynesian authority** Suppose the monetary authority believes that there is a fully exploitable trade-off between inflation and unemployment, i.e., ${\bar{\theta }}_{1\mathbf {a }}=0$. Then, in equilibrium, the conjectured policy multiplier ${\bar{\xi }} _{2}^{*}={\hat{\beta }}^{*}$ is strictly negative. A positive-target-inflation equilibrium policy results:

34 $$\begin{aligned} a^{*}=-\frac{\theta _{0}^{*}{\hat{\beta }}^{*}}{1+{\hat{\beta }}^{*}\left( {\hat{\beta }}^{*}+\theta _{1{\mathbf {a}}}^{*}\right) }\quad \text {and}\quad {\bar{\theta }}=\left( \theta _{0}^{*}\left( \frac{1+\hat{ \beta }^{*2}}{1+{\hat{\beta }}^{*}\left( {\hat{\beta }}^{*}+\theta _{1 {\mathbf {a}}}^{*}\right) }\right) ,0\right) . \end{aligned}$$

By Proposition 10, such a policy is hyperactive if $\theta _{1{\mathbf {a}}}^{*}>0$, hypoactive if $\theta _{1{\mathbf {a}}}^{*}<0$, and objectively optimal if $\theta _{1{\mathbf {a}}}^{*}=0$.

To sum up, the two equilibria feature new-classical nonintervention a la Friedman-Hayek and Keynesian activism, respectively. Regardless of the true model economy, such policy prescriptions emerge through suitable dogmatic beliefs.

#### A new-classical world

So far we did not fix a specific economic model. Now, by way of example, assume that a Lucas-Sargent model economy $\theta ^{*}=(\theta _{0}^{*},-{\hat{\beta }}^{*})\in {\tilde{\Theta }}$ is the true model, with no systematically exploitable trade-off between inflation and unemployment. Then, the pair $\left( a^{*},\delta \left( {\bar{\theta }} \right) \right)$ is a self-confirming equilibrium if and only if $a^{*}=-\theta _{0}^{*}({\hat{\beta }}^{*}+{\bar{\theta }}_{1{\mathbf {a}}})$ and ${\bar{\theta }}_{0}=\theta _{0}^{*}(1-({\hat{\beta }}^{*}+{\bar{\theta }}_{1 {\mathbf {a}}})^{2})$. Hence, the policy and belief pair

$$\begin{aligned} \left( -\theta _{0}^{*}\left( {\hat{\beta }}^{*}+{\bar{\theta }}_{1\mathbf { a}}\right) ,\delta \left( \theta _{0}^{*}\left( 1-\left( {\hat{\beta }} ^{*}+{\bar{\theta }}_{1{\mathbf {a}}}\right) ^{2}\right) ,{\bar{\theta }}_{1 {\mathbf {a}}}\right) \right) \end{aligned}$$

is the dogmatic self-confirming equilibrium in a Lucas-Sargent model economy. By Proposition 10, policy $a^{*}$ is hyperactive when ${\bar{\theta }}_{1{\mathbf {a}}}<\theta _{1{\mathbf {a}}}^{*}$ and objectively optimal when ${\bar{\theta }}_{1{\mathbf {a}}}=\theta _{1{\mathbf {a}} }^{*}$. The welfare loss is $\ell \left( a^{*},\theta ^{*}\right) =\theta _{0}^{*2}({\hat{\beta }}^{*}+{\bar{\theta }}_{1{\mathbf {a}} })^{2}$.

Next we consider two different equilibria in this new-classical world according to the monetary authority’s dogmatic beliefs.

New-classical authority Suppose the monetary authority correctly believes that there is no exploitable trade-off between inflation and unemployment, that is, $\mu ^{*}=\delta \left( {\bar{\theta }}_{0},-{\hat{\beta }}^{*}\right)$. The pair $\left( a^{*},\delta \left( {\bar{\theta }}_{0},-{\hat{\beta }}^{*}\right) \right)$ is a self-confirming equilibrium if and only if $a^{*}=0$ and ${\bar{\theta }}_{0}=\theta _{0}^{*}$. As a result, the policy and belief pair

35 $$\begin{aligned} \left( 0,\delta \left( \theta _{0}^{*},-{\hat{\beta }}^{*}\right) \right) \end{aligned}$$

is the new-classical self-confirming equilibrium. It features a zero-target-inflation policy, which is the objectively optimal policy (so, there is no welfare loss) as well as the fully revealing one that allows the authority to learn, in the long-run, the true coefficient $\theta _{0}^{*}$.

**Keynesian authority** Suppose the monetary authority wrongly believes that there is a fully exploitable trade-off between inflation and unemployment, with say $\mu ^{*}=\delta \left( {\bar{\theta }}_{0},0\right)$. The pair $(a^{*},\delta \left( {\bar{\theta }}_{0},0\right) )$ is a self-confirming equilibrium if and only if $a^{*}=-\theta _{0}^{*}{\hat{\beta }}^{*}$ and ${\bar{\theta }}_{0}=\theta _{0}^{*}(1-{\hat{\beta }}^{*2})$. The policy and belief pair

36 $$\begin{aligned} \left( -\theta _{0}^{*}{\hat{\beta }}^{*},\delta \left( \theta _{0}^{*}\left( 1-{\hat{\beta }}^{*2}\right) ,0\right) \right) \end{aligned}$$

is thus a Keynesian self-confirming equilibrium. It features an hyperactive positive-target-inflation policy. Since it is not the objectively optimal policy, the monetary authority suffers a welfare loss $\ell \left( a^{*},\theta ^{*}\right) =(\theta _{0}^{*}{\hat{\beta }}^{*})^{2}$.

#### A Keynesian world

What we noted above can be reversed as we consider the case of a Keynesian model economy where the policy multiplier $\xi _{2}$ is different from zero, i.e., the monetary authority may systematically reduce average unemployment. To consider a stark (although implausible) example, suppose that $\theta ^{*}=(\theta _{0}^{*},0)\in {\tilde{\Theta }}$ is the true model, that is, there is a full systematically exploitable trade-off between inflation and unemployment because monetary policy does not affect expectations ($\theta _{1{\mathbf {a}}}^{*}=0$). A Keynesian authority makes the objectively optimal positive-inflation choice in equilibrium. A new-classical authority chooses zero inflation, an inferior outcome.

#### Welfare consequences

What are the welfare implications of incorrect beliefs under dogmatism? By way of example, we consider a new-classical authority in a Keynesian economy, as well as a Keynesian authority in a new-classical economy. The loss of a new-classical zero inflation policy in a Keynesian economy, with $\theta _{1{\mathbf {a}}}^{*}=0$, is $(\theta _{0}^{*}{\hat{\beta }}^{*})^{2}$. It is the same loss of a Keynesian nonzero inflation policy (36) in a new-classical economy: a mistaken new-classical authority has the same lower welfare as a mistaken Keynesian one.

### Policy agnosticism and a curious interplay

#### Equilibria

Suppose that the monetary authority is not dogmatic, but has instead a two-model belief.^43^ Specifically, she is uncertain whether the true model is of the Lucas-Sargent or Samuelson-Solow kind and her self-confirming subjective belief $\mu ^{*}$ assigns positive probability mass to just one specimen of each kind, so that the (subjective) support consists of two points: a Lucas-Sargent model $(\theta _{0}^{ls}\left( \mu ^{*}\right) ,- {\hat{\beta }}^{*})$ and a Samuelson-Solow (Keynesian) model $\left( \theta _{0}^{ss}\left( \mu ^{*}\right) ,0\right)$. Denoting by $\mu _{k}^{*}\in \left[ 0,1\right]$ the subjective weight of the latter model, we can write belief $\mu ^{*}$ as

37 $$\begin{aligned} \mu ^{*}=\left( 1-\mu _{k}^{*}\right) \delta \left( \theta _{0}^{ls}\left( \mu ^{*}\right) ,-{\hat{\beta }}^{*}\right) +\mu _{k}^{*}\delta \left( \theta _{0}^{ss}\left( \mu ^{*}\right) ,0\right) . \end{aligned}$$

Since ${\mathbb {E}}_{\mu ^{*}}\left( \theta _{1{\mathbf {a}}}\right) =-\left( 1-\mu _{k}^{*}\right) {\hat{\beta }}^{*}$, the expected multiplier is ${\mathbb {E}}_{\mu ^{*}}(\xi _{2})=\mu _{k}^{*}{\hat{\beta }}^{*}$ and the pair $\left( a^{*},\mu ^{*}\right)$ is a self-confirming equilibrium if and only if

38 $$\begin{aligned} a^{*}=-\frac{\theta _{0}^{*}{\hat{\beta }}^{*}\mu _{k}^{*}}{1+ {\hat{\beta }}^{*}\mu _{k}^{*}\left( {\hat{\beta }}^{*}+\theta _{1 {\mathbf {a}}}^{*}\right) } \end{aligned}$$

and

39 $$\begin{aligned} \theta _{0}^{ls}\left( \mu ^{*}\right) =\frac{\theta _{0}^{*}}{1+ {\hat{\beta }}^{*}\left( {\hat{\beta }}^{*}+\theta _{1{\mathbf {a}}}^{*}\right) \mu _{k}^{*}},\ \ \theta _{0}^{ss}\left( \mu ^{*}\right) = \frac{\theta _{0}^{*}\left( 1+{\hat{\beta }}^{*2}\mu _{k}^{*}\right) }{1+{\hat{\beta }}^{*}\left( {\hat{\beta }}^{*}+\theta _{1\mathbf { a}}^{*}\right) \mu _{k}^{*}}. \end{aligned}$$

As a result, in this case, a pair of the form

$$\begin{aligned}&\left( -\frac{\theta _{0}^{*}{\hat{\beta }}^{*}\mu _{k}^{*}}{1+\hat{ \beta }^{*}\mu _{k}^{*}\left( {\hat{\beta }}^{*}+\theta _{1{\mathbf {a}} }^{*}\right) },\left( 1-\mu _{k}^{*}\right) \delta \left( \frac{ \theta _{0}^{*}}{1+{\hat{\beta }}^{*}\left( {\hat{\beta }}^{*}+\theta _{1{\mathbf {a}}}^{*}\right) \mu _{k}^{*}},-{\hat{\beta }}^{*}\right) \right. \\&\quad \left. +\mu _{k}^{*}\delta \left( \frac{\theta _{0}^{*}\left( 1+{\hat{\beta }} ^{*2}\mu _{k}^{*}\right) }{1+{\hat{\beta }}^{*}\left( {\hat{\beta }} ^{*}+\theta _{1{\mathbf {a}}}^{*}\right) \mu _{k}^{*}},0\right) \right) \end{aligned}$$

is a self-confirming equilibrium for every $\mu _{k}^{*}\in \left[ 0,1 \right]$. We thus have a continuum of equilibria parameterized by the subjective weight $\mu _{k}^{*}$ of the model of the Samuelson-Solow kind (and so by the expected multiplier $\mu _{k}^{*}{\hat{\beta }}^{*}$ ). In particular, the equilibrium policy $a^{*}$ is increasing in $\mu _{k}^{*}$: the higher the weight of the Keynesian model, the higher the planned inflation. If $\mu _{k}^{*}=0$ we get back to the dogmatic new-classical equilibrium, while if $\mu _{k}^{*}=1$ we get back to the dogmatic Keynesian equilibrium (Sect. 5.4.1).

In equilibrium, the coefficients (39) of the models of the Lucas-Sargent and Samuelson-Solow kind depend on the authority’s subjective weight $\mu _{k}^{*}$: different weights correspond to different Lucas-Sargent and Samuelson-Solow equilibrium specifications. Though the support of the equilibrium belief (37) always contains a specimen of both classes of model economies, that specimen changes as the weight $\mu _{k}^{*}$ changes. Finally, the welfare loss is

40 $$\begin{aligned} \ell \left( a^{*},\theta ^{*}\right) =\frac{\theta _{0}^{*2}\left( {\hat{\beta }}^{*}\mu _{k}^{*}+{\hat{\beta }}^{*}+\theta _{1 {\mathbf {a}}}^{*}\right) ^{2}}{\left( 1+{\hat{\beta }}^{*}\mu _{k}^{*}\left( {\hat{\beta }}^{*}+\theta _{1{\mathbf {a}}}^{*}\right) \right) ^{2}\left( 1+\left( {\hat{\beta }}^{*}+\theta _{1{\mathbf {a}}}^{*}\right) ^{2}\right) }. \end{aligned}$$

This curious interplay between the models deemed possible and the weight on each kind of model is our main finding for the two-model self-confirming belief; therefore, it will be further clarified in a prominent special case.

#### A new-classical world

Assume that a Lucas-Sargent model economy $\theta ^{*}=(\theta _{0}^{*},-{\hat{\beta }}^{*})$ is the true model. If so, by (38) and (39) the pair $\left( a^{*},\mu ^{*}\right)$ is a self-confirming equilibrium if and only if $a^{*}=-\theta _{0}^{*}{\hat{\beta }}^{*}\mu _{k}^{*}$, $\theta _{0}^{ls}\left( \mu ^{*}\right) =\theta _{0}^{*}$ and $\theta _{0}^{ss}\left( \mu ^{*}\right) =\theta _{0}^{*}(1+{\hat{\beta }}^{*2}\mu _{k}^{*})$. Hence, in this case, the pair

$$\begin{aligned} \left( -\theta _{0}^{*}{\hat{\beta }}^{*}\mu _{k}^{*},\left( 1-\mu _{k}^{*}\right) \delta \left( \theta _{0}^{*},-{\hat{\beta }}^{*}\right) +\mu _{k}^{*}\delta \left( \theta _{0}^{*}\left( 1+\beta ^{*2}\mu _{k}^{*}\right) ,0\right) \right) \end{aligned}$$

is a self-confirming equilibrium for every subjective weight $\mu _{k}^{*}\in \left[ 0,1\right]$. The welfare loss is $\ell \left( a^{*},\theta ^{*}\right) =(\theta _{0}^{*}{\hat{\beta }}^{*}\mu _{k}^{*})^{2}$.

As implied by the analysis of Sect. 5.5.1, we have a continuum of equilibria parameterized by the weight $\mu _{k}^{*}$ of the model of the Keynesian (Samuelson-Solow) kind: if $\mu _{k}^{*}>0$ the equilibrium policy is hyperactive, if $\mu _{k}^{*}=0$ we get the dogmatic new-classical equilibrium (35). Moreover, if $\mu _{k}^{*}=1$ we get back to the dogmatic Keynesian equilibrium (36). Now, however, the equilibrium coefficient $\theta _{0}^{ls}\left( \mu ^{*}\right)$ is pinned down by the true natural rate of unemployment $\theta _{0}^{*}$: the monetary authority understands that, if the true model were of the Lucas-Sargent kind, average unemployment and baseline unemployment at 0-planned inflation would coincide; furthermore, in the case under consideration the average rate of unemployment must be the natural rate. In contrast, the subjective equilibrium coefficient $\theta _{0}^{ss}\left( \mu ^{*}\right) =\theta _{0}^{*}(1+{\hat{\beta }}^{*2}\mu _{k}^{*})$ still depends on weight $\mu _{k}^{*}$: a higher subjective weight of the Samuelson-Solow specification corresponds to a higher planned inflation in equilibrium, hence, to a higher Phillips regression line, whose horizontal intercept is $\theta _{0}^{ss}\left( \mu ^{*}\right)$. Thus, the support of the equilibrium belief always contains a specimen of the Samuelson-Solow model; it, however, changes as $\mu _{k}^{*}$ changes. More generally, a two-model belief is determined by its (subjective) support and the relative likelihoods of the two models in the support. The self-confirming equilibrium conditions jointly discipline these two aspects of the belief.

**Fig. 4:**
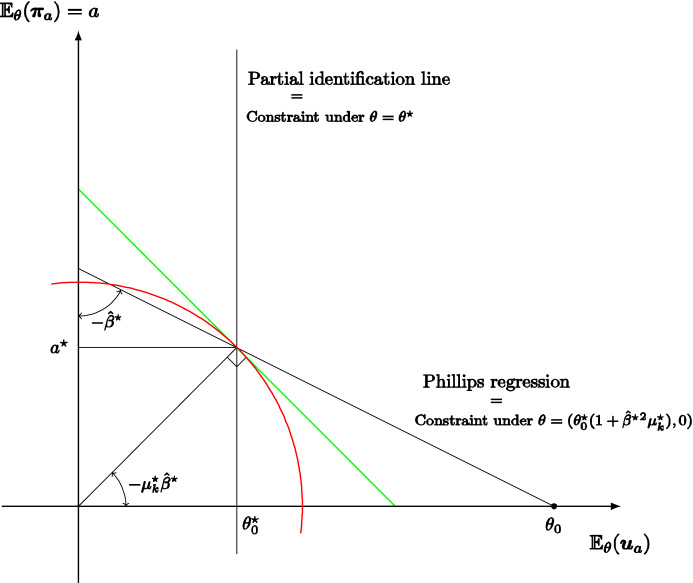
Two-model self-confirming equilibrium

Figure 4 illustrates. The monetary authority is uncertain about the true economic constraint, the vertical line at the natural rate of unemployment or the Phillips regression line. Since the true model is of the Lucas-Sargent kind, at a self-confirming equilibrium the average unemployment expected by the monetary authority must be the natural rate $\theta _{0}^{*}$; the subjective best reply condition is expressed by the tangency between the (red) indifference curve and a (green) line describing the expected constraint, the slope of which is intermediate between the vertical line at the natural rate $\theta _{0}^{*}$ and the Phillips regression line (which, in turn, depends on weight $\mu _{k}^{*}$ via the equilibrium relation $\theta _{0}=\theta _{0}^{*}(1+\hat{ \beta }^{*2}\mu _{k}^{*})$). Comparing the correct-belief equilibrium $\left( a,\mu \right) =\left( 0,\delta \left( \theta ^{*}\right) \right)$ with the represented self-confirming equilibrium determined by $\mu _{k}^{*}>0$, one can see that the latter features higher planned inflation $a^{*}>0$ and higher horizontal intercept $\theta _{0}^{ss}\left( \mu ^{*}\right) >\theta _{0}^{*}$.

**Fig. 5:**
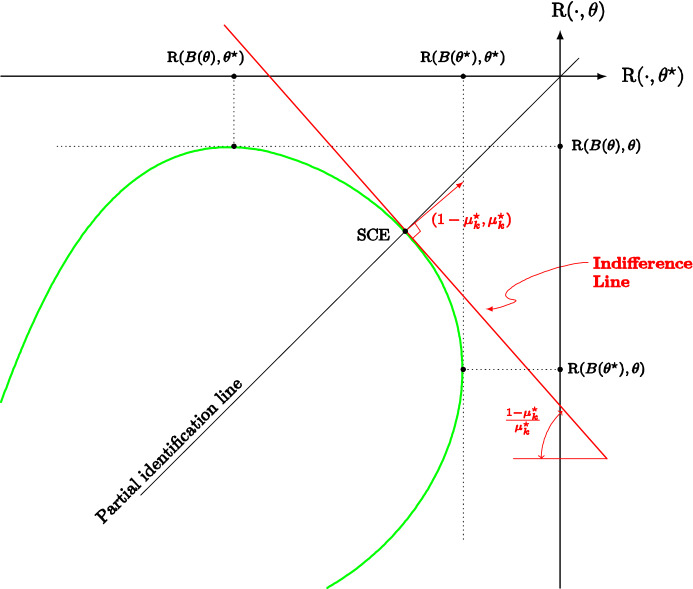
Self-confirming equilibrium in the expected rewards space

Figure 5 gives an alternative geometrical representation. Fix the true model $\theta ^{*}$ and an alternative model $\theta$. Every policy *a* induces a pair of objective expected rewards, the reward under model $\theta ^{*}$, $R(a,\theta ^{*})$, and the reward under model $\theta$, $R(a,\theta )$. By changing *a* one obtains the locus of possible pairs of rewards. If $R(a,\theta ^{*})\ne R(a,\theta )$, the monetary authority can infer which of the two models is true from the observed long-run average payoff. Therefore, the partial identification condition is $R(a,\theta ^{*})=R(a,\theta )$. At a self-confirming equilibrium $(a^{*},\mu ^{*})$ with ${\mathrm{supp}} \mu ^{*}=\{ \theta ^{*},\theta \}$, this belief-confirmation condition must hold; therefore, the equilibrium point $\left( R(a^{*},\theta ^{*}),R(a^{*},\theta )\right)$ is at the intersection of the main diagonal in the $\left( R(\cdot ,\theta ^{*}),R(\cdot ,\theta )\right)$-space, the “partial identification line,” with the locus of feasible pairs $\left\{ \left( R(a,\theta ^{*}),R(a,\theta )\right) :a\in A\right\}$, the constraint. At this intersection point, the constraint curve must be tangent to the constant-SEU line with slope $(1-\mu _{k}^{*})/\mu _{k}^{*}$.

Recall that $B\left( \cdot \right)$ denotes the best reply function. With this, $R\left( B\left( \theta ^{*}\right) ,\theta ^{*}\right) =V\left( B\left( \theta ^{*}\right) ,\delta \left( \theta ^{*}\right) \right) >V\left( B\left( \mu ^{*}\right) ,\mu ^{*}\right)$ . Indeed, $V\left( B\left( \theta ^{*}\right) ,\delta \left( \theta ^{*}\right) \right) >V\left( B\left( \mu ^{*}\right) ,\delta \left( \theta ^{*}\right) \right) =R\left( B\left( \mu ^{*}\right) ,\theta ^{*}\right)$ because $B\left( \mu ^{*}\right)$ is not a best reply to $\theta ^{*}$. On the other hand, $R\left( B\left( \mu ^{*}\right) ,\theta ^{*}\right) =V\left( B\left( \mu ^{*}\right) ,\mu ^{*}\right)$ because $R\left( B\left( \mu ^{*}\right) ,\cdot \right)$ is constant on the support of self-confirming belief $\mu ^{*}$ (see Lemma 2 and Proposition 4). The correct-belief equilibrium $\left( B\left( \theta ^{*}\right) ,\delta \left( \theta ^{*}\right) \right)$ features sharper beliefs than $\left( B\left( \mu ^{*}\right) ,\theta ^{*}\right)$. Therefore, this is an instance of Proposition 5: self-confirming equilibria with sharper beliefs yield higher values and lower losses.

## Concluding remarks

While applied theorists and economists more generally can benefit from seeing the self-confirming equilibrium concept in action, we think it is important to frame such applications within the context of an abstract analysis. Indeed, this allows to better understand key essential concepts like partial identification given the equilibrium choice, endogeneity of feedback about the state, and the role of observability of consequences. In this paper we put forward an abstract framework for the analysis of self-confirming policies amenable to economic applications, hence featuring both intrinsic randomness and (possibly) infinite spaces of actions and states. All the concepts and techniques can be extended to *n*-person games, but we focus on decision problems with uncertainty (i.e., one-person games with incomplete information) for several reasons. First, the analysis is simpler and it suffices for our examples and the monetary policy application. Second, it clarifies that self-confirming equilibrium is a *genuine* equilibrium concept also in a one-person setting, because equilibrium beliefs are disciplined by choice-dependent evidence. This should be contrasted with Harsanyi’s (1967) Bayesian equilibrium whereby subjective beliefs about unknown parameters are not disciplined by evidence; thus, in one-person settings Bayesian equilibrium just requires that the decision maker best reply to her subjective belief. Finally, we are not aware of simple and interesting *n*-person generalizations of our new comparative welfare results, Propositions 5 and 7. Our monetary policy application illustrates the abstract framework and extends previous work in several ways. In particular, it takes a more neutral perspective on the true model economy and it considers general beliefs rather than dogmatic ones. Besides the *n*-person case, several other extensions of the selfconfirming equilibrium idea are conceivable. Here we consider a few that we find worth exploring.

**Ambiguity aversion** It is possible to allow for non-neutral attitudes toward perceived ambiguity,^44^ e.g., by considering the smooth ambiguity model of Klibanoff et al. (2005), or the maxmin model of Gilboa and Schmeidler (1989). This is done in a companion paper (Battigalli et al., 2021). Here we give a hint of why such extension is immaterial in the examples and application of this paper. Go back to Fig. 5.b. Choices are represented as vectors of objective expected rewards. The best-reply condition requires that the set of feasible vectors is separated by the upper-contour set of vectors preferred to the chosen one, which under ambiguity aversion is convex. The key observation is that, in our examples and application, every undominated feasible vector is on the “efficient” boundary of the convex hull of the feasible vectors, i.e., it is not dominated by convex combinations of feasible vectors. By an intuitive application of the separating hyperplane theorem, this means that if a feasible vector is a best reply under ambiguity aversion, then it is also a best reply under ambiguity neutrality (subjective expected utility maximization), with the upper half-space delimited by the separating hyperplane as upper-contour set.

**Prospect Theory** It would be also natural to extend the selfconfirming equilibrium idea and its applications to prospect theory models *à la* Kahneman and Tversky (1979), see Wakker (2010) for an extensive treatment. The exercise is natural, but also challenging. On one hand, the equilibrium payoff is a natural (endogenous) reference point for the prospect theory analysis of selfconfirming equilibria. On the other hand, including the long-run empirical information represented by the partially identified set $\hat{ \Sigma }_{a}\left( \sigma \right)$ in a prospect theory model is less immediate than doing it in a smooth ambiguity or in a maxmin model. A possibility is to require the distortion functions featured by prospect theory (for gains and losses) to affect a specific model in ${\hat{\Sigma }} _{a}\left( \sigma \right)$. An alternative route is to consider “smooth ambiguity like” versions of prospect theory *à la* Vinogradov (2013), and require the equilibrium prior to be supported on ${\hat{\Sigma }}_{a}\left( \sigma \right)$ (this yields the previous approach if the decision maker’s prior is a Dirac measure at some point in ${\hat{\Sigma }}_{a}\left( \sigma \right)$). In any case, the problem definitely deserves more attention, and presents an avenue for future research. The works of Peter Wakker on prospect theory provide a starting point for this intriguing companion quest.

**Motivated beliefs** Kunda (1990) wrote an influential paper on how motivation influences reasoning. Since then, “motivated beliefs” became an important topic in psychology and also in economics, as exemplified in the Introduction by Epley and Gilovich to an interesting symposium on this topic in the 2016 summer issue of the * Journal of Economic Perspectives*. In their contribution, Benabou and Tirole (2016) cite reports of how agents neglect negative information, distort it, or choose not to obtain important information at little or no cost. Such behavior is explained in economic models where agents’ utility directly depends on their posterior beliefs and agents take this into account in forming their (action-dependent) beliefs. The self-confirming equilibrium (SCE) idea instead posits agents who take information more seriously and exploit all the information they obtain, given their choices. Despite such clear differences, SCE can be combined with belief-dependent motivations to explain an important stylized fact studied by the motivated-beliefs literature, i.e., the reluctance to acquire materially useful and cheap information (see Mannahan, 2021). Consider a decision maker (DM) with a prior belief $\mu$ over probabilistic models parameterized by an unknown personal trait $\theta \in \Theta \subset {\mathbb {R}}$, such as her intelligence, general ability, or health. Let $\mu ^{\prime }$ denote her realized posterior belief, conditional on the received message (material outcome) given her action. To fix ideas, let the DM’s “psychological utility” (Battigalli and Dufwenberg, forthcoming) be the sum of a standard utility function and an ego-utility component that depends on a posterior estimate of the unknown trait: ${\bar{v}}\left( m,a,\mu ^{\prime }\right) =v\left( m,a\right) +e\left( {\mathbb {E}}_{\mu ^{\prime }}\left( \varvec{\theta }\right) \right)$, where $e\left( \cdot \right)$ is an increasing function. The decision maker can either choose a status quo-action $a^{*}$ that yields a known (or learned) distribution of material outcomes, or an alternative action $a^{t}$ (e.g., taking a test) that yields a $\theta$-dependent distribution of material outcomes, and that would teach her about her trait. It may well be the case that, absent the ego-utility component, the best choice (possibly the dominant one) would be to take the test. But if function $e\left( \cdot \right)$ is concave, the expected variability of the posterior estimate ${\mathbb {E}}_{\mu ^{\prime }}\left( \varvec{\theta }\right)$ may make taking the test too “ego-risky” for the DM. In this case, the status-quo action $a^{*}$ would be an SCE action. This is somewhat similar to the preference for the status-quo in an SCE under smooth-ambiguity aversion (Battigalli et al. 2015), with concavity of the “second-order utility” replaced by concavity of ego-utility.

## Appendix A

### Appendix A.1: Additional mathematical preliminaries

For the theory of standard Borel spaces we refer to Mackey (1957) and Kechris (2012). First of all, note that if *X* is a standard Borel space, then ${\mathcal {X}}$ contains all the singletons. To prove it, it suffices to take a metric *d* on *X* that generates the Borel sigma algebra: ${\mathcal {X}} ={\mathcal {B}}({\mathcal {T}}_{d})$. For each $x\in X$ and all $n\in {\mathbb {N}}$, each open ball $\mathrm {B}_{1/n}\left( x\right) =\left\{ x^{\prime }\in X:d\left( x,x^{\prime }\right) <1/n\right\}$ belongs to the topology: $\mathrm {B}_{1/n}\left( x\right) \in {\mathcal {T}}_{d}\subseteq {\mathcal {X}}$. Then

$$\begin{aligned} \left\{ x\right\} = \mathop {\bigcap }\limits _{n\in {\mathbb {N}}}\mathrm {B} _{1/n}\left( x\right) \in {\mathcal {X}}. \end{aligned}$$

In particular, if *X* is countable, then ${\mathcal {X}}$ must be its power set.

Since several sigma algebras may be involved in the proofs, given a standard Borel space $\left( X,{\mathcal {X}}\right)$, we sometimes write ${\mathcal {B}} _{X}$ instead of ${\mathcal {X}}$ to denote its sigma algebra.

#### Lemma 4

*Let* *X* *and* *Y* *be measurable spaces and let* $\varphi :X\rightarrow Y$ *be measurable. Then* ${\hat{\varphi }}:\Delta (X)\rightarrow \Delta (Y)$ *is measurable with respect to the natural sigma algebra.*

#### Proof

The natural sigma algebra ${\mathcal {B}}_{\Delta \left( Y\right) }$ of $\Delta (Y)$ is generated by the sets of the form

$$\begin{aligned} \left\{ \nu \in \Delta \left( Y\right) :\nu \left( C\right) \le c\right\} \end{aligned}$$

for all $C\in {\mathcal {B}}_{Y}$ and $c\in {\mathbb {R}}$. Now, for all such sets

$$\begin{aligned} {\hat{\varphi }}^{-1}\left( \left\{ \nu \in \Delta \left( Y\right) :\nu \left( C\right) \le c\right\} \right)= & {} \left\{ \xi \in \Delta \left( X\right) : {\hat{\varphi }}(\xi )\in \left\{ \nu \in \Delta \left( Y\right) :\nu \left( C\right) \le c\right\} \right\} \\= & {} \left\{ \xi \in \Delta \left( X\right) :\left( \xi \circ \varphi ^{-1}\right) \left( C\right) \le c\right\} \\= & {} \left\{ \xi \in \Delta \left( X\right) :\xi \left( \varphi ^{-1}\left( C\right) \right) \le c\right\} , \end{aligned}$$

which belongs to ${\mathcal {B}}_{\Delta \left( X\right) }$ because the measurability of $\varphi$ guarantees that $\varphi ^{-1}\left( C\right) \in {\mathcal {B}}_{X}$, and ${\mathcal {B}}_{\Delta \left( X\right) }$ is generated by the sets of the form $\left\{ \xi \in \Delta \left( X\right) :\xi \left( B\right) \le b\right\}$ for all $B\in {\mathcal {B}}_{X}$ and $b\in {\mathbb {R}}$. Therefore, ${\hat{\varphi }}$ is measurable. $\square$

Note that this lemma does not require the measurable spaces $\left( X, {\mathcal {X}}\right)$ or $\left( Y,{\mathcal {Y}}\right)$ to be standard Borel, but rather hinges on the choice of the natural sigma algebras on $\Delta \left( X\right)$ and $\Delta \left( Y\right)$.

#### Lemma 5

*Let* *X* *and* *Y* *be standard Borel spaces and let* $\varphi :X\rightarrow Y$ *be measurable. If* $\varphi$ *is one-to-one, then* ${\hat{\varphi }}$ *is one-to-one. In this case:*

- $\varphi \left( X\right) \in {\mathcal {Y}}$*, hence* $\left( \varphi \left( X\right) ,{\mathcal {Y}}\cap \varphi \left( X\right) \right)$ *is a standard Borel space;*
- $\varphi :X\rightarrow \varphi \left( X\right)$ *is a measurable isomorphism;*
- ${\mathcal {X}}=\varphi ^{-1}\left( {\mathcal {Y}}\right)$*, that is,* $\varphi$ *generates* ${\mathcal {X}}$*;*
- ${\hat{\varphi }}:\Delta (X)\rightarrow \Delta (\varphi \left( X\right) )$ *is a measurable isomorphism (under the identification of* ${\hat{\varphi }}(\xi )$ *on* ${\mathcal {Y}}$ *with its restriction to* ${\mathcal {Y}}\cap \varphi \left( X\right)$*)*.

*In particular, the following three statements are equivalent:*

(i) $\varphi :X\rightarrow Y$ *is one-to-one;*
(ii) ${\hat{\varphi }}:\Delta (X)\rightarrow \Delta (Y)$ *is one-to-one;*
(iii) $$\varphi$$ *generates* $${\mathcal {X}}$$.

#### Proof

The proof is based on Mackey’s Monomorphism Theorem (henceforth MMT, see, Mackey, 1957, Theorem 3.2, and Kechris, 1994, Corollary 15.2).

**MMT** *If *$\varphi :X\rightarrow Y$ *is an injective and measurable map between two standard Borel spaces **X* *and **Y**, then *$\varphi \left( X\right) \in {\mathcal {B}}_{Y}$ *and *$\varphi$ *is a measurable isomorphism between *$\left( X, {\mathcal {B}}_{X}\right)$* and *$\left( \varphi \left( X\right) , {\mathcal {B}}_{Y}\cap \varphi \left( X\right) \right)$*. In particular,* $\varphi \left( B\right) \in {\mathcal {B}}_{Y}$ *for all *$B\in \mathcal { B}_{X}$.

By MMT, for each $B\in {\mathcal {B}}_{X}$, since $\varphi \left( B\right) \in {\mathcal {B}}_{Y}$, then $B=\varphi ^{-1}\left( \varphi \left( B\right) \right) \in \varphi ^{-1}\left( {\mathcal {B}}_{Y}\right)$, whence ${\mathcal {B}} _{X}\subseteq \varphi ^{-1}\left( {\mathcal {B}}_{Y}\right)$; and the converse inclusion follows from the measurability of $\varphi :X\rightarrow Y$. Thus $\varphi$ generates ${\mathcal {B}}_{X}$. This proves the first three points of the statement.

If $\xi ,\xi ^{\prime }\in \Delta \left( X\right)$, then ${\hat{\varphi }} \left( \xi \right) ={\hat{\varphi }}\left( \xi ^{\prime }\right)$ if and only if $\xi \left( \varphi ^{-1}\left( C\right) \right) =\xi ^{\prime }\left( \varphi ^{-1}\left( C\right) \right)$ for all $C\in {\mathcal {B}}_{Y}$, that is, if and only if $\xi$ and $\xi ^{\prime }$ coincide on the sigma algebra $\varphi ^{-1}\left( {\mathcal {B}}_{Y}\right)$ generated by $\varphi$. But $\varphi ^{-1}\left( {\mathcal {B}}_{Y}\right) ={\mathcal {B}}_{X}$, then $\xi \left( B\right) =\xi ^{\prime }\left( B\right)$ for all $B\in {\mathcal {B}} _{X}$, thus $\xi =\xi ^{\prime }$. Therefore, ${\hat{\varphi }}$ is one-to-one.

If ${\hat{\varphi }}:\Delta (X)\rightarrow \Delta (Y)$ is one-to-one, since it is measurable and the spaces are standard Borel, by MMT, it follows that ${\hat{\varphi }}\left( \Delta \left( X\right) \right)$ is a Borel subset of $\Delta \left( Y\right)$ and ${\hat{\varphi }}$ is a measurable isomorphism between $\left( \Delta \left( X\right) ,{\mathcal {B}}_{\Delta \left( X\right) }\right)$ and $\left( {\hat{\varphi }}\left( \Delta \left( X\right) \right) , {\mathcal {B}}_{\Delta \left( Y\right) }\cap {\hat{\varphi }}\left( \Delta \left( X\right) \right) \right)$.

With this, every element $\nu =\xi \circ \varphi ^{-1}$ of ${\hat{\varphi }} \left( \Delta \left( X\right) \right)$ is a probability measure on ${\mathcal {B}}_{Y}$, $\varphi \left( X\right) \in {\mathcal {B}}_{Y}$, and $\nu \left( \varphi \left( X\right) \right) =\xi \left( \varphi ^{-1}\left( \varphi \left( X\right) \right) \right) =\xi \left( X\right) =1$. Thus, the restriction $\iota \left( \nu \right)$ of an element $\nu \in {\hat{\varphi }} \left( \Delta \left( X\right) \right)$ to the sigma algebra ${\mathcal {B}} _{Y}\cap \varphi \left( X\right)$ of $\varphi \left( X\right)$ is an element of $\Delta \left( \varphi \left( X\right) \right)$. That is, the map

$$\begin{aligned} \begin{array}{cccc} \iota : &{} {\hat{\varphi }}\left( \Delta \left( X\right) \right) &{} \rightarrow &{} \Delta (\varphi \left( X\right) ) \\ &{} \nu &{} \mapsto &{} \iota \left( \nu \right) \end{array} \end{aligned}$$

that associates to each $\nu \in {\hat{\varphi }}\left( \Delta \left( X\right) \right)$ with its restriction $\iota \left( \nu \right)$ to ${\mathcal {B}} _{Y}\cap \varphi \left( X\right)$ is well defined.

We want to show that, $\iota$ is a measurable isomorphism between $\left( {\hat{\varphi }}\left( \Delta \left( X\right) \right) ,{\mathcal {B}}_{\Delta \left( Y\right) }\cap {\hat{\varphi }}\left( \Delta \left( X\right) \right) \right)$ and $\left( \Delta (\varphi \left( X\right) ),{\mathcal {B}}_{\Delta (\varphi \left( X\right) )}\right)$.^45^

By MMT again, it is sufficient to prove that it is bijective and measurable (since both spaces are standard Borel).

*Measurability:* First note that ${\mathcal {B}}_{\Delta (\varphi \left( X\right) )}$ is generated by the sets of the form $\left\{ \lambda \in \Delta \left( \varphi \left( X\right) \right) :\lambda \left( D\right) \le d\right\}$ for all $D\in {\mathcal {B}}_{\varphi \left( X\right) }={\mathcal {B}} _{Y}\cap \varphi \left( X\right)$ ($\subseteq {\mathcal {B}}_{Y}$) and $d\in {\mathbb {R}}$. Now, for all such sets

$$\begin{aligned}&\iota ^{-1}\left( \left\{ \lambda \in \Delta \left( \varphi \left( X\right) \right) :\lambda \left( D\right) \le d\right\} \right) \\&\quad =\left\{ \nu \in {\hat{\varphi }}\left( \Delta \left( X\right) \right) :\iota \left( \nu \right) \in \left\{ \lambda \in \Delta \left( \varphi \left( X\right) \right) :\lambda \left( D\right) \le d\right\} \right\} \\&\quad = \left\{ \nu \in {\hat{\varphi }}\left( \Delta \left( X\right) \right) :\iota \left( \nu \right) \left( D\right) \le d\right\} \\&\left. \text {(since }D\in {\mathcal {B}}_{\varphi \left( X\right) }={\mathcal {B}} _{Y}\cap \varphi \left( X\right) \text {)}\right. \\&\quad =\left\{ \nu \in \hat{ \varphi }\left( \Delta \left( X\right) \right) :\nu \left( D\right) \le d\right\} \\&\quad =\left\{ \nu \in \Delta \left( Y\right) :\nu \left( D\right) \le d\right\} \cap {\hat{\varphi }}\left( \Delta \left( X\right) \right) \in {\mathcal {B}} _{\Delta \left( Y\right) }\cap {\hat{\varphi }}\left( \Delta \left( X\right) \right) \end{aligned}$$

that is, $\iota$ is measurable.

*Injectivity:* Let $\nu ,\nu ^{\prime }\in {\hat{\varphi }}\left( \Delta \left( X\right) \right)$ be such that $\iota \left( \nu \right) =\iota \left( \nu ^{\prime }\right)$. For all $C\in {\mathcal {B}}_{Y}$,

$$\begin{aligned} \nu \left( C\cap \varphi \left( X\right) \right)\le & {} \nu \left( C\right) =\nu \left( C\cap \varphi \left( X\right) \right) +\nu \left( C\cap \varphi \left( X\right) ^{c}\right) \\\le & {} \nu \left( C\cap \varphi \left( X\right) \right) +\nu \left( \varphi \left( X\right) ^{c}\right) =\nu \left( C\cap \varphi \left( X\right) \right) , \end{aligned}$$

that is, $\nu \left( C\right) =\nu \left( C\cap \varphi \left( X\right) \right)$ and $C\cap \varphi \left( X\right) \in {\mathcal {B}}_{Y}\cap \varphi \left( X\right) ={\mathcal {B}}_{\varphi \left( X\right) }$, then $\nu \left( C\right) =\iota \left( \nu \right) \left( C\cap \varphi \left( X\right) \right)$. Since the same considerations apply to $\nu ^{\prime }$, it follows that

$$\begin{aligned} \nu \left( C\right) =\iota \left( \nu \right) \left( C\cap \varphi \left( X\right) \right) =\iota \left( \nu ^{\prime }\right) \left( C\cap \varphi \left( X\right) \right) =\nu ^{\prime }\left( C\right) , \end{aligned}$$

and so $$\nu =\nu ^{\prime }$$.

*Surjectivity:* Next we show that, for every $\lambda \in \Delta (\varphi \left( X\right) )$, the set function $\xi _{\lambda }\left( B\right) =\lambda \left( \varphi \left( B\right) \right)$ for all $B\in {\mathcal {B}}_{X}$ belongs to $\Delta \left( X\right)$ and $\iota \left( \hat{ \varphi }\left( \xi _{\lambda }\right) \right) =\lambda$. This is sufficient for surjectivity of $\iota$ because then $\lambda =\iota \left( \hat{\varphi }\left( \xi _{\lambda }\right) \right) \in \iota \left( {\hat{\varphi }}\left( \Delta \left( X\right) \right) \right)$. First observe that $\xi _{\lambda }:{\mathcal {B}}_{X}\rightarrow \left[ 0,1\right]$ is well defined because, by MMT, for all $B\in {\mathcal {B}}_{X}$, we have $\varphi \left( B\right) \in {\mathcal {B}}_{Y}\cap \varphi \left( X\right)$, and $\lambda :\left( \mathcal { B}_{Y}\cap \varphi \left( X\right) \right) \rightarrow \left[ 0,1\right]$. Moreover, denoting by $\psi :\varphi \left( X\right) \rightarrow X$ the inverse isomorphism of $\varphi$, for every $B\in {\mathcal {B}}_{X}$, $\varphi \left( B\right) =\psi ^{-1}\left( B\right)$.^46^

Thus $\xi _{\lambda }\left( B\right) =\lambda \left( \varphi \left( B\right) \right) =\lambda \left( \psi ^{-1}\left( B\right) \right)$ is a probability measure on *X*. Finally, for every $D\in {\mathcal {B}}_{Y}\cap \varphi \left( X\right)$,

$$\begin{aligned} \iota \left( {\hat{\varphi }}\left( \xi _{\lambda }\right) \right) \left( D\right) ={\hat{\varphi }}\left( \xi _{\lambda }\right) \left( D\right) =\xi _{\lambda }\left( \varphi ^{-1}\left( D\right) \right) =\lambda \left( \varphi \left( \varphi ^{-1}\left( D\right) \right) \right) =\lambda \left( D\right) \end{aligned}$$

as wanted.

So far, we have shown that, if $\varphi$ is injective, then the map

$$\begin{aligned} \begin{array}{cccc} {\tilde{\varphi }}=\iota \circ {\hat{\varphi }}: &{} \Delta \left( X\right) &{} \rightarrow &{} \Delta \left( \varphi \left( X\right) \right) \\ &{} \xi &{} \mapsto &{} \left( \xi \circ \varphi ^{-1}\right) _{|{\mathcal {B}} _{Y}\cap \varphi \left( X\right) } \end{array} \end{aligned}$$

is an isomorphism of standard Borel spaces; and that for all $\lambda \in \Delta (\varphi \left( X\right) )$, the set function $\xi _{\lambda }=\lambda \circ \varphi$, defined by $\left( \lambda \circ \varphi \right) \left( B\right) =\lambda \left( \varphi \left( B\right) \right)$ for all $B\in {\mathcal {B}}_{X}$, belongs to $\Delta \left( X\right)$ and $\lambda =\iota \left( {\hat{\varphi }}\left( \xi _{\lambda }\right) \right) =\tilde{ \varphi }\left( \lambda \circ \varphi \right)$; that is,

$$\begin{aligned} {\tilde{\varphi }}^{-1}\left( \lambda \right) =\lambda \circ \varphi \end{aligned}$$

for all $$\lambda \in \Delta \left( \varphi \left( X\right) \right)$$.

This proves the fourth point of the statement.

Finally, we already proved that if $\varphi$ is injective, then $\varphi$ generates ${\mathcal {B}}_{X}$, and that if $\varphi$ generates ${\mathcal {B}} _{X}$, then ${\hat{\varphi }}$ is injective. The proof is concluded by showing that, if ${\hat{\varphi }}$ is injective, so is $\varphi$. We will actually prove the contrapositive statement.

Recall that $\delta :X\rightarrow \Delta \left( X\right)$ is the Dirac embedding of *X* into $\Delta \left( X\right)$. For each $x\in X$, we have ${\hat{\varphi }}(\delta \left( x\right) )=\delta \left( \varphi \left( x\right) \right)$. In fact, for all $C\in {\mathcal {B}}_{Y}$,

41 $$\begin{aligned} {\hat{\varphi }}(\delta \left( x\right) )(C)= & {} \delta \left( x\right) \left( \varphi ^{-1}\left( C\right) \right) \nonumber \\= & {} \left\{ \begin{array}{ll} 1 &{} \quad \text {if }x\in \varphi ^{-1}\left( C\right) \text {, i.e. }\varphi \left( x\right) \in C \\ 0 &{} \quad \text {if }x\notin \varphi ^{-1}\left( C\right) \text {, i.e. } \varphi \left( x\right) \notin C \end{array} \right. =\delta \left( \varphi \left( x\right) \right) \left( C\right) \text {.} \end{aligned}$$

Therefore, if $\varphi$ is not one-to-one, ${\hat{\varphi }}$ is not one-to-one. In fact, if there are $x\ne z$ in *X* such that $\varphi \left( x\right) =\varphi \left( z\right)$, then

$$\begin{aligned} {\hat{\varphi }}(\delta \left( x\right) )=\delta \left( \varphi \left( x\right) \right) =\delta \left( \varphi \left( z\right) \right) ={\hat{\varphi }}(\delta \left( z\right) )\text {,} \end{aligned}$$

but $\delta \left( x\right)$ and $\delta \left( z\right)$ are different probability measures on ${\mathcal {X}}$ because the latter contains all singletons. $\square$

At the opposite side of the spectrum we have the case of constant functions.

#### Lemma 6

*Let* *X* *and* *Y* *be standard Borel spaces and let* $\varphi :X\rightarrow Y$ *be measurable. Then* $\varphi$ *is constant if and only if* ${\hat{\varphi }}$ *is constant.*

#### Proof

If $\varphi \equiv {\bar{y}}$ is constant, then given any $\sigma \in \Delta \left( X\right)$ and any $C\in {\mathcal {Y}}$,

$$\begin{aligned} {\hat{\varphi }}\left( \sigma \right) \left( C\right)= & {} \sigma \left( \varphi ^{-1}\left( C\right) \right) =\sigma \left( \left\{ x\in X:\varphi \left( x\right) \in C\right\} \right) \\= & {} \sigma \left( \left\{ x\in X:{\bar{y}}\in C\right\} \right) =\delta \left( {\bar{y}}\right) \left( C\right) \end{aligned}$$

irrespective of $$\sigma$$.

The converse is proved by contraposition. If $\varphi$ is not constant, then there exist $x\ne y$ such that $\varphi \left( x\right) \ne \varphi \left( y\right)$, but then

$$\begin{aligned} {\hat{\varphi }}(\delta \left( x\right) )=\delta \left( \varphi \left( x\right) \right) \ne \delta \left( \varphi \left( y\right) \right) ={\hat{\varphi }} (\delta \left( y\right) ) \end{aligned}$$

where the two external equalities follow from (41) and the internal inequality by the fact that $\varphi \left( x\right) \ne \varphi \left( y\right)$ and singletons are measurable in *C*. $\square$

Let $\left( X,{\mathcal {X}}\right)$, $\left( Y,{\mathcal {Y}}\right)$, and $\left( Z,{\mathcal {Z}}\right)$ be measurable spaces, $f:X\rightarrow Y$ and $g:Y\rightarrow Z$, and $h:X\rightarrow Z$. It is convenient to denote by ${\mathcal {F}}=f^{-1}\left( {\mathcal {Y}}\right) \subseteq {\mathcal {X}}$, ${\mathcal {G}}=g^{-1}\left( {\mathcal {Z}}\right) \subseteq {\mathcal {Y}}$, and ${\mathcal {H}}=h^{-1}\left( {\mathcal {Z}}\right) \subseteq {\mathcal {X}}$ the sigma algebras generated by *f*, *g*,  and *h*, respectively. Moreover, we will say that *h* is *f**-measurable* if it is ${\mathcal {F}}$-${\mathcal {Z}}$ -measurable.

#### Lemma 7

*Let* $\left( X,{\mathcal {X}}\right)$ *be a measurable space, and let* $\left( Y,{\mathcal {Y}}\right)$ *and* $\left( Z, {\mathcal {Z}}\right)$ *be standard Borel spaces. Then the following conditions are equivalent for two measurable functions* $f:X\rightarrow Y$ *and* $h:X\rightarrow Z$:

(i) *h* *is* *f*-*measurable —that is,* $h^{-1}\left( {\mathcal {Z}} \right) \subseteq f^{-1}\left( {\mathcal {Y}}\right)$*;*
(ii) *there exists a measurable function* $g:Y\rightarrow Z$ *such that* $h=g\circ f$.

#### Proof

See, e.g., Chow and Teicher 1997, Theorem 1.4.4. $\square$

The next result completes Lemma 1.

#### Corollary 2

*Let* $\left( \Theta ,{\mathcal {B}}_{\Theta }\right)$ *and* $\left( S,{\mathcal {B}}_{S}\right)$ *be Borel spaces and fix a map* $p:\Theta \rightarrow \Delta \left( S\right)$. *Then* *p* *is measurable if and only if*

$$\begin{aligned} \forall B\in {\mathcal {B}}_{S},\forall b\in {\mathbb {R}},\ \ \left\{ \theta \in \Theta :p\left( \theta \right) \left( B\right) \le b\right\} \in \mathcal {B }_{\Theta }, \end{aligned}$$

*that is,* $\theta \mapsto p\left( \theta \right) \left( B\right)$ *is measurable for all* $B\in {\mathcal {B}}_{S}$. *If, moreover,* *p* *is one-to-one, then:*

- $$p\left( \Theta \right) \in {\mathcal {B}}_{\Delta \left( S\right) }$$*;*
- $p:\Theta \rightarrow p\left( \Theta \right)$ *is a measurable isomorphism;*
- ${\tilde{p}}:\Delta \left( \Theta \right) \rightarrow \Delta \left( p\left( \Theta \right) \right)$ *defined by* ${\tilde{p}}\left( \mu \right) \left( B\right) =\mu \left( p^{-1}\left( B\right) \right)$ ($B\in \mathcal {B }_{p\left( \Theta \right) }$) *is a measurable isomorphism and, for every* $\lambda \in \Delta \left( p\left( \Theta \right) \right)$*, the inverse image of* $\lambda$ *through* ${\tilde{p}}$ is $\lambda \circ p$.

#### Proof

Since ${\mathcal {B}}_{\Delta \left( S\right) }$ is the sigma algebra generated by the functions $\phi _{B}:\Delta \left( S\right) \rightarrow {\mathbb {R}}$ defined by $\phi _{B}\left( \xi \right) =\xi \left( B\right)$ for all $B\in {\mathcal {B}}_{S}$, a map $p:\Theta \rightarrow \Delta \left( S\right)$ is measurable if and only if $\phi _{B}\circ p$ is measurable for all $B\in {\mathcal {B}}_{S}$ (see, e.g., Berberian 1997, Proposition 1.3.8). But, given any $B\in {\mathcal {B}}_{S}$, $p\left( B\mid \theta \right) =p\left( \theta \right) \left( B\right) =\left( \phi _{B}\circ p\right) \left( \theta \right)$ for all $\theta \in \Theta$ , thus $p\left( B\mid \cdot \right) =\phi _{B}\circ p$, proving the first part of the statement.^47^ The rest follows from the statement of Lemma 5 setting $X=\Theta$, $Y=\Delta \left( S\right)$, and $\varphi =p$, with the exception of the explicit expression ${\tilde{p}} ^{-1}\left( \lambda \right) =\lambda \circ p$, for which the last paragraph of the proof of Lemma 5 has to be inspected. $\square$

#### Corollary 3

*Let* $\left( \Theta ,{\mathcal {B}}_{\Theta }\right)$ *and* $\left( T,{\mathcal {B}}_{T}\right)$ *be Borel spaces and fix* $q\in \Delta \left( T\right)$. *Then*

$$\begin{aligned} \begin{array}{cccc} p: &{} \Theta &{} \rightarrow &{} \Delta \left( T\times \Theta \right) \\ &{} \theta &{} \mapsto &{} q\times \delta \left( \theta \right) =p\left( \theta \right) \end{array} \end{aligned}$$

*is measurable and one-to-one.*

#### Proof

Injectivity is obvious, so we have only to show that

$$\begin{aligned} \forall B\in {\mathcal {B}}_{T\times \Theta },\forall b\in {\mathbb {R}},\ \ \left\{ \theta \in \Theta :p\left( \theta \right) \left( B\right) \le b\right\} \in {\mathcal {B}}_{\Theta }, \end{aligned}$$

that is, $\theta \mapsto q\times \delta \left( \theta \right) \left( B\right)$ is measurable for all $B\in {\mathcal {B}}_{T}\times {\mathcal {B}} _{\Theta }$. Now for each $\theta \in \Theta$,

$$\begin{aligned} q\times \delta \left( \theta \right) \left( B\right) =\int _{\Theta }q\left( B^{\eta }\right) d\delta \left( \theta \right) \left( \eta \right) =q\left( B^{\theta }\right) , \end{aligned}$$

where $B^{\theta }=\left\{ t\in T:\left( t,\theta \right) \in B\right\}$, and a crucial step in the proof of the Fubini-Tonelli Theorem (see, e.g., Billingsley 2012, p. 246) consists precisely in showing that the map $\theta \mapsto q\left( B^{\theta }\right)$ is measurable for all $B\in {\mathcal {B}}_{T}\times {\mathcal {B}}_{\Theta }$. $\square$

#### Corollary 4

*Let* $\left( S,{\mathcal {B}}_{S}\right)$ *be a Borel space. Then:* $\delta \left( S\right) \in {\mathcal {B}}_{\Delta \left( S\right) }$*,* $\delta :S\rightarrow \delta \left( S\right)$ *is a measurable isomorphism, and* $\lambda \mapsto \lambda \circ \delta$ *is a measurable isomorphism between* $\Delta \left( \delta \left( S\right) \right)$ *and* $\Delta \left( S\right)$.

#### Proof

In order to apply the previous Corollary 2 with $\Theta =S$ and $p=\delta$, we have only to verify that $\left\{ s\in S:\delta \left( s\right) \left( B\right) \le b\right\} \in {\mathcal {B}}_{S}$ for all $B\in {\mathcal {B}}_{S}$ and $b\in {\mathbb {R}}$; but this follows from the fact that $\left\{ s\in S:\delta \left( s\right) \left( B\right) \le b\right\} =\left\{ s\in S:1_{B}\left( s\right) \le b\right\}$ and indicators of measurable sets are measurable functions. $\square$

### Appendix A.2: Feedback and identification

First recall that, for each $a\in A$, $f_{a}:S\rightarrow M$ is measurable and so is ${\hat{f}}_{a}:\Delta \left( S\right) \rightarrow \Delta \left( M\right)$. Since $\Sigma \in {\mathcal {B}}_{\Delta \left( S\right) }$, and points are measurable in standard Borel spaces, then for every $\nu \in \Delta \left( M\right)$ the set

$$\begin{aligned} \left\{ \sigma ^{\prime }\in \Sigma :{\hat{f}}_{a}(\sigma ^{\prime })=\nu \right\} =\left\{ \sigma ^{\prime }\in \Delta \left( S\right) :{\hat{f}} _{a}(\sigma ^{\prime })=\nu \right\} \cap \Sigma \in {\mathcal {B}}_{\Delta \left( S\right) }\cap \Sigma ={\mathcal {B}}_{\Sigma }, \end{aligned}$$

and so ${\hat{\Sigma }}_{a}\left( \sigma \right) =\left\{ \sigma ^{\prime }\in \Sigma :{\hat{f}}_{a}(\sigma ^{\prime })={\hat{f}}_{a}\left( \sigma \right) \right\}$ is a measurable subset of both $\Sigma$ and $\Delta \left( S\right)$ for all $\sigma \in \Sigma$.

For the next result, recall that $\rho :A\times S\rightarrow C$ is the consequence function and that the feedback functions considered here satisfy observability of consequences, which is a maintained assumption.

#### Lemma 8

*Let* *f* *and* $f^{\prime }$ *be feedback functions for a decision problem* *D*. *Then:*

(i) $\rho$ *is coarser than* *f**;*
(ii) *if* $f_{a}$ *is one-to-one for every* $a\in A$*, then* $f^{\prime }$ *is coarser than* *f**;*
(iii) *if* $f^{\prime }$ *is coarser than* *f**, then* ${\hat{\Sigma }} _{a}\left( \sigma \right) \subseteq {\hat{\Sigma }}_{a}^{\prime }\left( \sigma \right)$ *for all* $\left( a,\sigma \right) \in A\times \Sigma$.

#### Proof

(i) Since consequences are observable, for each action $a\in A$ there exists a measurable function $g_{a}:M\rightarrow C$ such that $\rho _{a}\left( s\right) =g_{a}\left( f_{a}\left( s\right) \right)$ for all $s\in S$. (ii) By assumption, for each $a\in A$, $f_{a}:S\rightarrow M$ is Borel measurable and one-to-one. By Lemma 5, $f_{a}\left( S\right)$ is a Borel subset of *M* and $f_{a}:S\rightarrow f_{a}\left( S\right)$ is a Borel isomorphism. Then, the inverse function $f_{a}^{-1}:f_{a}\left( S\right) \rightarrow S$ is Borel measurable.^48^ Arbitrarily choose ${\bar{s}}\in S$ and set

$$\begin{aligned} k_{a}\left( m\right) \equiv \left\{ \begin{array}{ll} f_{a}^{-1}\left( m\right) &{} \quad m\in f_{a}\left( S\right) \text {,} \\ {\bar{s}} &{} \quad m\notin f_{a}\left( S\right) \text {.} \end{array} \right. \end{aligned}$$

It is easy to see that $k_{a}$ defines a Borel measurable map from *M* to *S* such that, for every $s\in S$,

$$\begin{aligned} f_{a}^{\prime }\left( s\right) =f_{a}^{\prime }\left( f_{a}^{-1}\left( f_{a}\left( s\right) \right) \right) =f_{a}^{\prime }\left( k_{a}\left( f_{a}\left( s\right) \right) \right) =\left( f_{a}^{\prime }\circ k_{a}\right) \left( f_{a}\left( s\right) \right) \text {.} \end{aligned}$$

Setting $h_{a}=f_{a}^{\prime }\circ k_{a}:M\rightarrow M^{\prime }$ yields the desired result. (iii) Let $\left( a,\sigma \right) \in A\times \Sigma$. For every $\sigma ^{\prime }\in {\hat{\Sigma }}_{a}\left( \sigma \right)$, $\sigma ^{\prime }\left( f_{a}^{-1}\left( B_{M}\right) \right) =\sigma \left( f_{a}^{-1}\left( B_{M}\right) \right)$ for all $B_{M}\in {\mathcal {B}}_{M}$. But $h_{a}^{-1}\left( B_{M^{\prime }}\right) \in {\mathcal {B}}_{M}$ for all $B_{M^{\prime }}\in {\mathcal {B}}_{M^{\prime }}$, then

$$\begin{aligned} \sigma ^{\prime }\left( \left( f_{a}^{\prime }\right) ^{-1}\left( B_{M^{\prime }}\right) \right)&=\sigma ^{\prime }\left( \left( h_{a}\circ f_{a}\right) ^{-1}\left( B_{M^{\prime }}\right) \right) =\sigma ^{\prime }\left( f_{a}^{-1}\left( h_{a}^{-1}\left( B_{M^{\prime }}\right) \right) \right) \\&=\sigma \left( f_{a}^{-1}\left( h_{a}^{-1}\left( B_{M^{\prime }}\right) \right) \right) =\sigma \left( \left( h_{a}\circ f_{a}\right) ^{-1}\left( B_{M^{\prime }}\right) \right) =\sigma \left( \left( f_{a}^{\prime }\right) ^{-1}\left( B_{M^{\prime }}\right) \right) \end{aligned}$$

and $$\sigma ^{\prime }\in {\hat{\Sigma }}_{a}^{\prime }\left( \sigma \right)$$. $$\square$$

### Appendix A.3: Additional definitions

The *self-confirming *(*equilibrium*) *correspondence*

$$\begin{aligned} \Gamma :\Sigma \rightarrow 2^{A\times \Delta \left( \Sigma \right) } \end{aligned}$$

associates to each possible true model $\sigma ^{*}$ the collection $\Gamma \left( \sigma ^{*}\right)$ of its self-confirming equilibria $\left( a^{*},\mu ^{*}\right)$. It is also convenient to consider the (*equilibrium*) *action* *correspondence*

$$\begin{aligned} \gamma :\Sigma \rightarrow 2^{A} \end{aligned}$$

that associates each possible true model $\sigma ^{*}$ with the collection $\gamma \left( \sigma ^{*}\right)$ of its * self-confirming *(*equilibrium*) *actions*, that is, actions $a^{*}$ such that $\left( a^{*},\mu ^{*}\right) \in \Gamma \left( \sigma ^{*}\right)$ for some belief $\mu ^{*}$.

### Appendix A.4: Model uncertainty

Let $\mu ^{*}\ll \nu ^{*}$ denote the $\mu ^{*}$ is absolutely continuous with respect to (informally, “sharper than”) $\nu ^{*}$. We show that self-confirming equilibria with sharper basic subjective assessments have higher values, hence —by Proposition 4— lower losses.

#### Proposition 12

*If* $\left( a^{*},\mu ^{*}\right) ,\left( b^{*},\nu ^{*}\right) \in \Gamma \left( \sigma ^{*}\right)$ *and* $\mu ^{*}\ll \nu ^{*}$*, then* $R\left( a^{*},\sigma ^{*}\right) =V\left( a^{*},\mu ^{*}\right) \ge V\left( b^{*},\nu ^{*}\right) =R\left( b^{*},\sigma ^{*}\right)$.

#### Proof

Since $\mu ^{*}\left( {\hat{\Sigma }}_{a^{*}}\left( \sigma ^{*}\right) \right) =1$ and $\nu ^{*}\left( \hat{ \Sigma }_{b^{*}}\left( \sigma ^{*}\right) \right) =1$, then $\mu ^{*}\ll \nu ^{*}$ implies $\mu ^{*}\left( {\hat{\Sigma }}_{b^{*}}\left( \sigma ^{*}\right) \right) =1$ and so $\mu ^{*}\left( \hat{ \Sigma }_{b^{*}}\left( \sigma ^{*}\right) \cap {\hat{\Sigma }}_{a^{*}}\left( \sigma ^{*}\right) \right) =1$. The optimality condition (6) for $a^{*}$ and Proposition 4 deliver

$$\begin{aligned} R\left( a^{*},\sigma ^{*}\right)= & {} V\left( a^{*},\mu ^{*}\right) \ge \int _{{\hat{\Sigma }}_{a^{*}}\left( \sigma ^{*}\right) }R\left( b^{*},\sigma \right) d\mu ^{*}\left( \sigma \right) \\= & {} \int _{ {\hat{\Sigma }}_{a^{*}}\left( \sigma ^{*}\right) \cap {\hat{\Sigma }} _{b^{*}}\left( \sigma ^{*}\right) }R\left( b^{*},\sigma \right) d\mu ^{*}\left( \sigma \right) . \end{aligned}$$

Since, by Lemma 2, $R\left( b^{*},{\sigma }\right) =R\left( b^{*},{\sigma }^{*}\right)$ for all $\sigma \in \hat{\Sigma }_{b^{*}}\left( \sigma ^{*}\right)$, it follows that $V\left( a^{*},\mu ^{*}\right) \ge R\left( b^{*},\sigma ^{*}\right) =V\left( b^{*},\nu ^{*}\right)$, where the last equality follows from Proposition 4. $\square$

Priors $\mu ^{*}$ and $\nu ^{*}$ that are mutually absolutely continuous are called *equivalent*, denoted $\mu ^{*}\sim \nu ^{*}$; this means that they agree on what models are possible (or impossible). By the previous result, if $\mu ^{*}\sim \nu ^{*}$ then $V\left( a^{*},\mu ^{*}\right) =V\left( b^{*},\nu ^{*}\right)$ for all pairs of self-confirming equilibria $\left( a^{*},\mu ^{*}\right) ,\left( b^{*},\nu ^{*}\right) \in \Gamma \left( \sigma ^{*}\right)$. The value of self-confirming equilibria is thus pinned down by what the decision maker deems possible, whereas the specific shape of the prior is value-irrelevant. But more is actually true: actions can be exchanged across such self-confirming equilibria.

#### Proposition 13

*If* $\left( a^{*},\mu ^{*}\right) ,\left( b^{*},\nu ^{*}\right) \in \Gamma \left( \sigma ^{*}\right)$ *and* $\mu ^{*}\sim \nu ^{*}$*, then* $\left( a^{*},\nu ^{*}\right) ,\left( b^{*},\mu ^{*}\right) \in \Gamma \left( \sigma ^{*}\right)$.

#### Proof

As observed, $R\left( a^{*},\sigma ^{*}\right) =V\left( a^{*},\mu ^{*}\right) =V\left( b^{*},\nu ^{*}\right) =R\left( b^{*},\sigma ^{*}\right)$, but then

- $R\left( b^{*},\sigma ^{*}\right) =V\left( a^{*},\mu ^{*}\right) \ge V\left( a,\mu ^{*}\right)$ for all $a\in A$ and $\mu ^{*}\left( {\hat{\Sigma }}_{b^{*}}\left( \sigma ^{*}\right) \right) =1$ since $\nu ^{*}\left( {\hat{\Sigma }}_{b^{*}}\left( \sigma ^{*}\right) \right) =1$;
- $R\left( a^{*},\sigma ^{*}\right) =V\left( b^{*},\nu ^{*}\right) \ge V\left( b,\nu ^{*}\right)$ for all $b\in A$ and $\nu ^{*}\left( {\hat{\Sigma }}_{a^{*}}\left( \sigma ^{*}\right) \right) =1$ since $\mu ^{*}\left( {\hat{\Sigma }}_{a^{*}}\left( \sigma ^{*}\right) \right) =1$.

$$\square$$

The results on the value that we just established for subjective model uncertainty extend to objective model uncertainty. In particular, self-confirming (equilibrium) actions with better identification properties have higher values, regardless of which confirmed beliefs support them.

#### Proposition 14

*If* $\left( a^{*},\mu ^{*}\right) ,\left( b^{*},\nu ^{*}\right) \in \Gamma \left( \sigma ^{*}\right)$ *and* $\hat{ \Sigma }_{a^{*}}(\sigma ^{*})\subseteq {\hat{\Sigma }}_{b^{*}}(\sigma ^{*})$*, then* $V\left( a^{*},\mu ^{*}\right) \ge V\left( b^{*},\nu ^{*}\right)$.

#### Proof

The optimality condition (6) for $a^{*}$ and Proposition 4 deliver

$$\begin{aligned} R\left( a^{*},\sigma ^{*}\right) =V\left( a^{*},\mu ^{*}\right) \ge \int _{{\hat{\Sigma }}_{a^{*}}\left( \sigma ^{*}\right) }R\left( b^{*},\sigma \right) d\mu ^{*}\left( \sigma \right) =\int _{ {\hat{\Sigma }}_{b^{*}}\left( \sigma ^{*}\right) }R\left( b^{*},\sigma \right) d\mu ^{*}\left( \sigma \right) \end{aligned}$$

but, by Lemma 2, $R\left( b^{*},{\sigma }\right) =R\left( b^{*},{\sigma }^{*}\right)$ for all $\sigma \in \hat{\Sigma }_{b^{*}}\left( \sigma ^{*}\right)$, it follows that

$$\begin{aligned} V\left( a^{*},\mu ^{*}\right) \ge R\left( b^{*},\sigma ^{*}\right) =V\left( b^{*},\nu ^{*}\right) \end{aligned}$$

where the last equality follows from Proposition 4. $\square$

Also observe that, if ${\hat{\Sigma }}_{a^{*}}\left( \sigma ^{*}\right) ={\hat{\Sigma }}_{b^{*}}\left( \sigma ^{*}\right)$, then $R\left( a^{*},\sigma ^{*}\right) =V\left( a^{*},\mu ^{*}\right) =V\left( b^{*},\nu ^{*}\right) =R\left( b^{*},\sigma ^{*}\right)$. With this, if $\left( a^{*},\mu ^{*}\right) ,\left( b^{*},\nu ^{*}\right) \in \Gamma \left( \sigma ^{*}\right)$ then

- $R\left( b^{*},\sigma ^{*}\right) =V\left( a^{*},\mu ^{*}\right) \ge V\left( a,\mu ^{*}\right)$ for all $a\in A$ and $\mu ^{*}\left( {\hat{\Sigma }}_{b^{*}}\left( \sigma ^{*}\right) \right) =1$ since $\mu ^{*}\left( {\hat{\Sigma }}_{a^{*}}\left( \sigma ^{*}\right) \right) =1$;
- $R\left( a^{*},\sigma ^{*}\right) =V\left( b^{*},\nu ^{*}\right) \ge V\left( b,\nu ^{*}\right)$ for all $b\in A$ and $\nu ^{*}\left( {\hat{\Sigma }}_{a^{*}}\left( \sigma ^{*}\right) \right) =1$ since $\nu ^{*}\left( {\hat{\Sigma }}_{b^{*}}\left( \sigma ^{*}\right) \right) =1$.

Finally, the following results relate self-confirming equilibrium actions to objectively optimal actions:

#### Corollary 5

*A fully revealing action is self-confirming if and only if it is objectively optimal.*

Under own-action independence of feedback about the state, we have a stronger result. Eq. (5) and Lemma 2 imply:

#### Corollary 6

*Under own-action independence of feedback about the state, an action is self-confirming if and only if it is objectively optimal.*

Thus, from a decision perspective, own-action independence of feedback is equivalent to perfect feedback.

### Appendix A.5: Other proofs

#### Proof of Lemma 1

See Lemma 5. $$\square$$

#### Proof of Proposition 1

See Lemma 7.$$\square$$

#### Proof of Proposition 2

See Lemma 8. $$\square$$

#### Proof of Lemma 2

Fix $a\in A$. Observability of consequences implies that $\rho _{a}\left( s\right) =g_{a}\left( f_{a}\left( s\right) \right)$ for each $s\in S$, where $g_{a}:M\rightarrow C$ is ${\mathcal {B}}_{M}-{\mathcal {B}}_{C}$-measurable; as $f_{a}:S\rightarrow M$ is ${\mathcal {F}}_{a}-{\mathcal {B}}_{M}$-measurable, then $\rho _{a}:S\rightarrow C$ is ${\mathcal {F}}_{a}-{\mathcal {B}}_{C}$-measurable. Moreover, $v:C\rightarrow {\mathbb {R}}$ is ${\mathcal {B}}_{C}-{\mathcal {B}}_{ {\mathbb {R}}}$-measurable and bounded above, and so $r_{a}=v\circ \rho _{a}:S\rightarrow {\mathbb {R}}$ is ${\mathcal {F}}_{a}-{\mathcal {B}}_{{\mathbb {R}}}$ -measurable and bounded above. Thus,

42 $$\begin{aligned} \forall \sigma \in \Delta \left( S\right) ,\ \ R_{a}\left( {\sigma }\right) =\int _{S}r_{a}d\sigma =\int _{S}r_{a}d\sigma _{|{\mathcal {F}}_{a}}. \end{aligned}$$

In particular, if $\sigma \in \Sigma$ and $\sigma ^{\prime }\in {\hat{\Sigma }} _{a}(\sigma )$, then $R_{a}\left( {\sigma }\right) =\int _{S}r_{a}d\sigma _{| {\mathcal {F}}_{a}}=\int _{S}r_{a}d\sigma _{|{\mathcal {F}}_{a}}^{\prime }=R_{a}(\sigma ^{\prime })$. $\square$

#### Proof of Proposition 4

If $\left( a^{*},\mu ^{*}\right) \in A\times \Delta \left( \Sigma \right)$ and $\mu ^{*}\left( {\hat{\Sigma }}_{a^{*}}(\sigma ^{*})\right) =1$, then

$$\begin{aligned} V\left( a^{*},\mu ^{*}\right) =\int _{\Sigma }R\left( a^{*},\sigma \right) d\mu ^{*}\left( \sigma \right) =\int _{{\hat{\Sigma }} _{a^{*}}(\sigma ^{*})}R\left( a^{*},\sigma \right) d\mu ^{*}\left( \sigma \right) =R\left( a^{*},\sigma ^{*}\right) , \end{aligned}$$

because, by Lemma 2, $R\left( a^{*},\sigma \right) =R\left( a^{*},\sigma ^{*}\right)$ for all $\sigma \in {\hat{\Sigma }} _{a^{*}}(\sigma ^{*})$. $\square$

#### Proof of Proposition 5

It follows immediately from Proposition 4 and Proposition 12. $\square$

#### Proof of Proposition 6

It follows immediately from Proposition 4 and Proposition 14. $\square$

#### Proof of Proposition 7

Suppose that ${\hat{\Sigma }}_{a}(\sigma ^{*})\subseteq {\hat{\Sigma }}_{b}(\sigma ^{*})$ for each $b\in A$. We already observed that if *a* is objectively optimal, then $\left( a,\delta \left( \sigma ^{*}\right) \right) \in \Gamma \left( \sigma ^{*}\right)$ and $a\in \gamma \left( \sigma ^{*}\right)$. As for the converse, let $\mu ^{*}\in \Delta \left( \Sigma \right)$ be such that $\left( a,\mu ^{*}\right) \in \Gamma \left( \sigma ^{*}\right)$. Since ${\hat{\Sigma }}_{a}(\sigma ^{*})\subseteq {\hat{\Sigma }}_{b}(\sigma ^{*})$ for each $b\in A$ and, by Lemma 2, for each *b* it is true that $R\left( b,\sigma \right) =R\left( b,\sigma ^{*}\right)$ when $\sigma \in {\hat{\Sigma }}_{b}(\sigma ^{*})$, then $R\left( a,\sigma ^{*}\right) \ge \int _{{\hat{\Sigma }} _{a}(\sigma ^{*})}R\left( b,\sigma \right) d\mu ^{*}\left( \sigma \right) =R\left( b,\sigma ^{*}\right)$, as wanted. $\square$

#### Proof of Corollary 5

Given a true model $\sigma ^{*}\in \Sigma$, the result follows from Proposition 7 since if *a* is fully revealing, then ${\hat{\Sigma }} _{a}(\sigma ^{*})=\left\{ \sigma ^{*}\right\} \subseteq \hat{\Sigma }_{a^{\prime }}(\sigma ^{*})$ for every $a^{\prime }\in A$. $\square$

#### Proof of Corollary 6

Given a true model $\sigma ^{*}\in \Sigma$, the result follows from Proposition 7 since own-action independence of feedback implies $\hat{\Sigma }_{a}(\sigma ^{*})={\hat{\Sigma }}_{a^{\prime }}(\sigma ^{*})$ for every $a,a^{\prime }\in A$. Hence, $\gamma \left( \sigma ^{*}\right) =\arg \max _{a\in A}R\left( a,\sigma ^{*}\right)$. $\square$

#### Proof of Proposition 8

Recall that *a* is fixed. We first prove the inclusion $\subseteq$. If $\theta ^{\prime }\in {\hat{\Sigma }}_{a}\left( \theta \right)$, then ${\hat{\rho }} _{a}\left( q\times \delta \left( \theta ^{\prime }\right) \right) ={\hat{\rho }} _{a}\left( q\times \delta \left( \theta \right) \right)$. In particular,

43 $$\begin{aligned} \int _{S}h\left( \rho _{a}\right) d\left( q\times \delta \left( \theta ^{\prime }\right) \right) =\int _{S}h\left( \rho _{a}\right) d\left( q\times \delta \left( \theta \right) \right) , \end{aligned}$$

for all $h:{\mathbb {R}}\times {\mathbb {R}}\rightarrow {\mathbb {R}}$ for which the integral is defined. Next observe that:

1. For $h\left( u,\pi \right) =\pi$ and $\theta ^{\prime \prime }\in \Theta$ , we have that $\int _{S}\varvec{\pi }d\left( q\times \delta _{\theta ^{\prime \prime }}\right) =a$.
2. For $h\left( u,\pi \right) =\pi ^{2}$ and $\theta ^{\prime \prime }\in \Theta$, we have that $\int _{S}\varvec{\pi }^{2}d\left( q\times \delta \left( \theta ^{\prime \prime }\right) \right) =a^{2}+\left( \theta _{3}^{\prime \prime }\right) ^{2}$.
3. For $h\left( u,\pi \right) =u$ and $\theta ^{\prime \prime }\in \Theta$, we have that $\int _{S}{\varvec{u}}d\left( q\times \delta \left( \theta ^{\prime \prime }\right) \right) =\theta _{0}^{\prime \prime }+\left( \theta _{1\varvec{\pi }}^{\prime \prime }+\theta _{1{\mathbf {a}}}^{\prime \prime }\right) a$.
4. For $h\left( u,\pi \right) =u^{2}$ and $\theta ^{\prime \prime }\in \Theta$, we have that $\int _{S}{\varvec{u}}^{2}d\left( q\times \delta \left( \theta ^{\prime \prime }\right) \right) =\left( \theta _{0}^{\prime \prime }+\left( \theta _{1\varvec{\pi }}^{\prime \prime }+\theta _{1\mathbf {a }}^{\prime \prime }\right) a\right) ^{2}+\left( \theta _{1\varvec{\pi } }^{\prime \prime }\right) ^{2}\left( \theta _{3}^{\prime \prime }\right) ^{2}+\left( \theta _{2}^{\prime \prime }\right) ^{2}$.
5. For $h\left( u,\pi \right) =u\pi$ and $\theta ^{\prime \prime }\in \Theta$, we have that $\int _{S}\varvec{u\pi }d\left( q\times \delta \left( \theta ^{\prime \prime }\right) \right) =a\left( \theta _{0}^{\prime \prime }+\left( \theta _{1\varvec{\pi }}^{\prime \prime }+\theta _{1\mathbf {a }}^{\prime \prime }\right) a\right) +\theta _{1\varvec{\pi }}^{\prime \prime }\left( \theta _{3}^{\prime \prime }\right) ^{2}$.

Given (43), note that point 2 gives $\theta _{3}^{\prime }=\theta _{3}$, then points 3 and 5 give $\theta _{1\varvec{\pi }}^{\prime }=\theta _{1\varvec{\pi }}$. With this, point 3 again yields $\theta _{0}^{\prime }+\theta _{1{\mathbf {a}}}^{\prime }a=\theta _{0}+\theta _{1 {\mathbf {a}}}a$. Then point 4 gives

$$\begin{aligned}&\left( \theta _{0}^{\prime }+\left( \theta _{1\varvec{\pi }}^{\prime }+\theta _{1{\mathbf {a}}}^{\prime }\right) a\right) ^{2}+\left( \theta _{1 \varvec{\pi }}^{\prime }\right) ^{2}\left( \theta _{3}^{\prime }\right) ^{2}+\left( \theta _{2}^{\prime }\right) ^{2}\\&\quad =\left( \theta _{0}+\left( \theta _{1\varvec{\pi }}+\theta _{1{\mathbf {a}}}\right) a\right) ^{2}+\left( \theta _{1\varvec{\pi }}\right) ^{2}\left( \theta _{3}\right) ^{2}+\left( \theta _{2}\right) ^{2}; \end{aligned}$$

point 3 says that the first summands on both sides coincide, and we already established $\left( \theta _{1\varvec{\pi }}^{\prime }\right) ^{2}\left( \theta _{3}^{\prime }\right) ^{2}=\left( \theta _{1\varvec{\pi }}\right) ^{2}\left( \theta _{3}\right) ^{2}$; therefore, $\theta _{2}^{\prime }=\theta _{2}$. This concludes the proof of the first set inclusion and formalizes the moments heuristics described in the main text.

In order to obtain the opposite inclusion, note that some simple algebra delivers, for each $\theta ^{\prime \prime }\in \Theta$ and each $\left( u,\pi \right) \in {\mathbb {R}}^{2}$,

$$\begin{aligned} {\hat{\rho }}_{a}\left( q\times \delta \left( \theta ^{\prime \prime }\right) \right) \left( \left( -\infty ,u\right] \times \left( -\infty ,\pi \right] \right) =q\left( Q_{\theta ^{\prime \prime }}\right) , \end{aligned}$$

where $$Q_{\theta ^{\prime \prime }}=\left\{ \left( w,\varepsilon \right) \in W\times E:\left. \begin{array}{l} w\theta _{2}^{\prime \prime }+\varepsilon \theta _{1\varvec{\pi }}^{\prime \prime }\theta _{3}^{\prime \prime }\le u-\left( \theta _{0}^{\prime \prime }+a\theta _{1{\mathbf {a}}}^{\prime \prime }\right) -a\theta _{1\varvec{\pi } }^{\prime \prime } \\ \theta _{3}^{\prime \prime }\varepsilon \le \pi -a \end{array} \right. \right\}$$.

Now, consider $\theta ^{\prime }\in \Theta$ such that $\theta _{0}^{\prime }+\theta _{1{\mathbf {a}}}^{\prime }a=\theta _{0}+\theta _{1{\mathbf {a}}}a$, $\theta _{1\varvec{\pi }}^{\prime }=\theta _{1\varvec{\pi }}$, $\theta _{2}^{\prime }=\theta _{2}$, $\theta _{3}^{\prime }=\theta _{3}$, then $Q_{\theta ^{\prime }}=Q_{\theta }$, hence ${\hat{\rho }}_{a}\left( q\times \delta \left( \theta ^{\prime }\right) \right) \left( \left( -\infty ,u \right] \times \left( -\infty ,\pi \right] \right) ={\hat{\rho }}_{a}\left( q\times \delta \left( \theta \right) \right) \left( \left( -\infty ,u\right] \times \left( -\infty ,\pi \right] \right)$ implying that ${\hat{\rho }} _{a}\left( q\times \delta \left( \theta ^{\prime }\right) \right) ={\hat{\rho }} _{a}\left( q\times \delta \left( \theta \right) \right)$ and $\theta ^{\prime }\in {\hat{\Sigma }}_{a}\left( \theta \right)$. $\square$

#### Proof of Lemma 3

Some simple algebra shows that

$$\begin{aligned} R\left( a,\theta \right)&=-\int _{W\times E}{\varvec{u}}^{2}\left( a,w,\varepsilon ,\theta \right) dq\left( w,\varepsilon \right) -\int _{W\times E}\varvec{\pi }^{2}\left( a,w,\varepsilon ,\theta \right) dq\left( w,\varepsilon \right) \\&=-\left( \theta _{0}+\left( \theta _{1\varvec{\pi }}+\theta _{1{\mathbf {a}} }\right) a\right) ^{2}-a^{2}-\theta _{2}^{2}-\theta _{3}^{2}\theta _{1 \varvec{\pi }}^{2}-\theta _{3}^{2} \\&=-{\mathbb {E}}_{\theta }^{2}\left( {\varvec{u}}_{a}\right) -{\mathbb {E}} _{\theta }^{2}\left( \varvec{\pi }_{a}\right) -\theta _{2}^{2}-\theta _{3}^{2}\theta _{1\varvec{\pi }}^{2}-\theta _{3}^{2} \\&=v\left( {\mathbb {E}}_{\theta }\left( {\varvec{u}}_{a}\right) ,{\mathbb {E}} _{\theta }\left( \varvec{\pi }_{a}\right) \right) +\kappa , \end{aligned}$$

where, being ${\tilde{\Theta }}=\left\{ \left( \theta _{0},\theta _{1{\mathbf {a}} }\right) \right\} ={\mathbb {R}}^{2}$, we set $\kappa =-\theta _{2}^{2}-\theta _{3}^{2}\theta _{1\varvec{\pi }}^{2}-\theta _{3}^{2}$ since this polynomial can be regarded as a constant term. $\square$

#### Proof of Proposition 9

It holds

$$\begin{aligned} R\left( a,\theta \right) =-\left( \left( \theta _{1\varvec{\pi }}+\theta _{1 {\mathbf {a}}}\right) ^{2}+1\right) a^{2}-2\theta _{0}\left( \theta _{1\varvec{\pi }}+\theta _{1{\mathbf {a}}}\right) a+\kappa . \end{aligned}$$

Thus, $V\left( a,\mu ^{*}\right)$ is —up to a constant— equal to

$$\begin{aligned}&-\int _{{\hat{\Sigma }}_{a^{*}}\left( \theta ^{*}\right) }\left( \left( \left( {\hat{\beta }}^{*}+\theta _{1{\mathbf {a}}}\right) ^{2}+1\right) a^{2}+2\theta _{0}\left( {\hat{\beta }}^{*}+\theta _{1{\mathbf {a}}}\right) a\right) d\mu ^{*}\left( \theta \right) \\&\quad =-\int _{{\mathbb {R}}}\left( \left( \left( {\hat{\beta }}^{*}+\theta _{1 {\mathbf {a}}}\right) ^{2}+1\right) a^{2}+2\left( \theta _{0}^{*}+\left( \theta _{1{\mathbf {a}}}^{*}-\theta _{1{\mathbf {a}}}\right) a^{*}\right) \left( {\hat{\beta }}^{*}+\theta _{1{\mathbf {a}}}\right) a\right) d\mu ^{*}\left( \theta _{1{\mathbf {a}}}\right) \\&\quad =-\int _{{\mathbb {R}}}\left( \left( {\hat{\beta }}^{*2}+\theta _{1{\mathbf {a}} }^{2}+2{\hat{\beta }}^{*}\theta _{1{\mathbf {a}}}+1\right) a^{2}+2\theta _{0}^{*}\left( {\hat{\beta }}^{*}+\theta _{1{\mathbf {a}}}\right) a+2a^{*}\left( \theta _{1{\mathbf {a}}}^{*}-\theta _{1{\mathbf {a}} }\right) \left( {\hat{\beta }}^{*}+\theta _{1{\mathbf {a}}}\right) a\right) d\mu ^{*}\left( \theta _{1{\mathbf {a}}}\right) \\&\quad =-\int _{{\mathbb {R}}}\left( \left( {\hat{\beta }}^{*2}+\theta _{1{\mathbf {a}} }^{2}+2{\hat{\beta }}^{*}\theta _{1{\mathbf {a}}}+1\right) a^{2}+2\theta _{0}^{*}\left( {\hat{\beta }}^{*}+\theta _{1{\mathbf {a}}}\right) a+2a^{*}\left( \theta _{1{\mathbf {a}}}^{*}{\hat{\beta }}^{*}+\theta _{1{\mathbf {a}}}^{*}\theta _{1{\mathbf {a}}}-\theta _{1{\mathbf {a}}}{\hat{\beta }} ^{*}-\theta _{1{\mathbf {a}}}^{2}\right) a\right) d\mu ^{*}\left( \theta _{1{\mathbf {a}}}\right) \\&\quad =-\left( {\hat{\beta }}^{*2}+{\mathbb {E}}_{\mu ^{*}}\left( \theta _{1 {\mathbf {a}}}^{2}\right) +2{\hat{\beta }}^{*}{\mathbb {E}}_{\mu ^{*}}\left( \theta _{1{\mathbf {a}}}\right) +1\right) a^{2}-2\theta _{0}^{*}\left( \hat{ \beta }^{*}+{\mathbb {E}}_{\mu ^{*}}\left( \theta _{1{\mathbf {a}}}\right) \right) a \\&\qquad -2a^{*}\left( \theta _{1{\mathbf {a}}}^{*}{\hat{\beta }}^{*}+\theta _{1{\mathbf {a}}}^{*}{\mathbb {E}}_{\mu ^{*}}\left( \theta _{1{\mathbf {a}} }\right) -{\mathbb {E}}_{\mu ^{*}}\left( \theta _{1{\mathbf {a}}}\right) \hat{ \beta }^{*}-{\mathbb {E}}_{\mu ^{*}}\left( \theta _{1{\mathbf {a}} }^{2}\right) \right) a. \end{aligned}$$

The first order condition $\partial V\left( a,\mu ^{*}\right) /\partial a=0$ implies

$$\begin{aligned}&a\left( {\hat{\beta }}^{*2}+{\mathbb {E}}_{\mu ^{*}}\left( \theta _{1 {\mathbf {a}}}^{2}\right) +2{\hat{\beta }}^{*}{\mathbb {E}}_{\mu ^{*}}\left( \theta _{1{\mathbf {a}}}\right) +1\right) \\&\quad +a^{*}\left( \theta _{1{\mathbf {a}} }^{*}{\hat{\beta }}^{*}+\theta _{1{\mathbf {a}}}^{*}{\mathbb {E}}_{\mu ^{*}}\left( \theta _{1{\mathbf {a}}}\right) -{\mathbb {E}}_{\mu ^{*}}\left( \theta _{1{\mathbf {a}}}\right) {\hat{\beta }}^{*}-{\mathbb {E}}_{\mu ^{*}}\left( \theta _{1{\mathbf {a}}}^{2}\right) \right) \\&\quad =-\theta _{0}^{*}\left( {\hat{\beta }}^{*}+{\mathbb {E}}_{\mu ^{*}}\left( \theta _{1{\mathbf {a}}}\right) \right) . \end{aligned}$$

Putting $a=a^{*}$ we get

$$\begin{aligned} a^{*}\left( {\hat{\beta }}^{*2}+{\hat{\beta }}^{*}{\mathbb {E}}_{\mu ^{*}}\left( \theta _{1{\mathbf {a}}}\right) +1+\theta _{1{\mathbf {a}}}^{*} {\hat{\beta }}^{*}+\theta _{1{\mathbf {a}}}^{*}{\mathbb {E}}_{\mu ^{*}}\left( \theta _{1{\mathbf {a}}}\right) \right) =-\theta _{0}^{*}\left( {\hat{\beta }}^{*}+{\mathbb {E}}_{\mu ^{*}}\left( \theta _{1{\mathbf {a}} }\right) \right) \end{aligned}$$

and so

$$\begin{aligned} a^{*}&=\frac{-\theta _{0}^{*}\left( {\hat{\beta }}^{*}+{\mathbb {E}} _{\mu ^{*}}\left( \theta _{1{\mathbf {a}}}\right) \right) }{{\hat{\beta }} ^{*2}+{\hat{\beta }}^{*}{\mathbb {E}}_{\mu ^{*}}\left( \theta _{1 {\mathbf {a}}}\right) +1+\theta _{1{\mathbf {a}}}^{*}{\hat{\beta }}^{*}+\theta _{1{\mathbf {a}}}^{*}{\mathbb {E}}_{\mu ^{*}}\left( \theta _{1 {\mathbf {a}}}\right) } \\&=-\frac{\theta _{0}^{*}\left( {\hat{\beta }}^{*}+{\mathbb {E}}_{\mu ^{*}}\left( \theta _{1{\mathbf {a}}}\right) \right) }{1+\left( {\hat{\beta }} ^{*}+\theta _{1{\mathbf {a}}}^{*}\right) \left( {\hat{\beta }}^{*}+ {\mathbb {E}}_{\mu ^{*}}\left( \theta _{1{\mathbf {a}}}\right) \right) }. \end{aligned}$$

As a result, ${\hat{\Sigma }}_{a^{*}}\left( \theta ^{*}\right)$ is equal to

$$\begin{aligned} \left\{ \left( \theta _{0},\theta _{1{\mathbf {a}}}\right) \in {\mathbb {R}} ^{2}:\theta _{0}=\theta _{0}^{*}-\frac{\theta _{0}^{*}\left( \hat{ \beta }^{*}+{\mathbb {E}}_{\mu ^{*}}\left( \theta _{1{\mathbf {a}}}\right) \right) }{1+\left( {\hat{\beta }}^{*}+\theta _{1{\mathbf {a}}}^{*}\right) \left( {\hat{\beta }}^{*}+{\mathbb {E}}_{\mu ^{*}}\left( \theta _{1\mathbf { a}}\right) \right) }\left( \theta _{1{\mathbf {a}}}^{*}-\theta _{1{\mathbf {a}} }\right) \right\} , \end{aligned}$$

as desired. $$\square$$

#### Proof of Proposition 10

It holds

$$\begin{aligned} a^{*}-a^{o}&=\frac{\theta _{0}^{*}\left( {\hat{\beta }}^{*}+\theta _{1{\mathbf {a}}}^{*}\right) }{1+\left( {\hat{\beta }}^{*}+\theta _{1 {\mathbf {a}}}^{*}\right) ^{2}}-\frac{\theta _{0}^{*}\left( {\hat{\beta }} ^{*}+{\mathbb {E}}_{\mu ^{*}}\left( \theta _{1{\mathbf {a}}}\right) \right) }{1+\left( {\hat{\beta }}^{*}+\theta _{1{\mathbf {a}}}^{*}\right) \left( {\hat{\beta }}^{*}+{\mathbb {E}}_{\mu ^{*}}\left( \theta _{1\mathbf { a}}\right) \right) } \\&=\frac{\theta _{0}^{*}\left( \left( {\hat{\beta }}^{*}+\theta _{1 {\mathbf {a}}}^{*}\right) \left( 1+\left( {\hat{\beta }}^{*}+\theta _{1 {\mathbf {a}}}^{*}\right) \left( {\hat{\beta }}^{*}+{\mathbb {E}}_{\mu ^{*}}\left( \theta _{1{\mathbf {a}}}\right) \right) \right) -\left( {\hat{\beta }} ^{*}+{\mathbb {E}}_{\mu ^{*}}\left( \theta _{1{\mathbf {a}}}\right) \right) \left( 1+\left( {\hat{\beta }}^{*}+\theta _{1{\mathbf {a}}}^{*}\right) ^{2}\right) \right) }{\left( 1+\left( {\hat{\beta }}^{*}+\theta _{1 {\mathbf {a}}}^{*}\right) ^{2}\right) \left( 1+\left( {\hat{\beta }}^{*}+\theta _{1{\mathbf {a}}}^{*}\right) \left( {\hat{\beta }}^{*}+{\mathbb {E}} _{\mu ^{*}}\left( \theta _{1{\mathbf {a}}}\right) \right) \right) } \\&=\frac{\theta _{0}^{*}\left( \theta _{1{\mathbf {a}}}^{*}+\left( \hat{ \beta }^{*}+\theta _{1{\mathbf {a}}}^{*}\right) ^{2}\left( {\hat{\beta }} ^{*}+{\mathbb {E}}_{\mu ^{*}}\left( \theta _{1{\mathbf {a}}}\right) \right) -{\mathbb {E}}_{\mu ^{*}}\left( \theta _{1{\mathbf {a}}}\right) -\left( {\hat{\beta }}^{*}+{\mathbb {E}}_{\mu ^{*}}\left( \theta _{1 {\mathbf {a}}}\right) \right) \left( {\hat{\beta }}^{*}+\theta _{1{\mathbf {a}} }^{*}\right) ^{2}\right) }{\left( 1+\left( {\hat{\beta }}^{*}+\theta _{1 {\mathbf {a}}}^{*}\right) ^{2}\right) \left( 1+\left( {\hat{\beta }}^{*}+\theta _{1{\mathbf {a}}}^{*}\right) \left( {\hat{\beta }}^{*}+{\mathbb {E}} _{\mu ^{*}}\left( \theta _{1{\mathbf {a}}}\right) \right) \right) } \\&=\frac{\theta _{0}^{*}\left( \theta _{1{\mathbf {a}}}^{*}-{\mathbb {E}} _{\mu ^{*}}\left( \theta _{1{\mathbf {a}}}\right) \right) }{\left( 1+\left( {\hat{\beta }}^{*}+\theta _{1{\mathbf {a}}}^{*}\right) ^{2}\right) \left( 1+\left( {\hat{\beta }}^{*}+\theta _{1{\mathbf {a}}}^{*}\right) \left( {\hat{\beta }}^{*}+{\mathbb {E}}_{\mu ^{*}}\left( \theta _{1{\mathbf {a}} }\right) \right) \right) }. \end{aligned}$$

Hence, if $a^{*}\ne 0,$ it holds

$$\begin{aligned} a^{*}-a^{o}=-\frac{a^{*}}{1+\left( {\hat{\beta }}^{*}+\theta _{1 {\mathbf {a}}}^{*}\right) ^{2}}\frac{\theta _{1{\mathbf {a}}}^{*}-\mathbb {E }_{\mu ^{*}}\left( \theta _{1{\mathbf {a}}}\right) }{{\hat{\beta }}^{*}+ {\mathbb {E}}_{\mu ^{*}}\left( \theta _{1{\mathbf {a}}}\right) } \end{aligned}$$

and so

44 $$\begin{aligned} a^{*}\gtrless a^{o}\Longleftrightarrow a^{*}\frac{\theta _{1\mathbf {a }}^{*}-{\mathbb {E}}_{\mu ^{*}}\left( \theta _{1{\mathbf {a}}}\right) }{ {\hat{\beta }}^{*}+{\mathbb {E}}_{\mu ^{*}}\left( \theta _{1{\mathbf {a}} }\right) }\lessgtr 0. \end{aligned}$$

Having established this relation, we can now prove points (i) and (iii) (points (ii) and (iv) being obvious).

(i) Suppose $a^{*}>a^{o}$. By (27) ${\mathbb {E}}_{\mu ^{*}}\left( \theta _{1{\mathbf {a}}}\right) \ne -{\hat{\beta }}^{*}$ and so ${\mathbb {E}}_{\mu ^{*}}\left( \theta _{1{\mathbf {a}}}\right) <-\hat{\beta }^{*}$. By (44), $\left( \theta _{1{\mathbf {a}}}^{*}- {\mathbb {E}}_{\mu ^{*}}\left( \theta _{1{\mathbf {a}}}\right) \right) /(\hat{ \beta }^{*}+{\mathbb {E}}_{\mu ^{*}}\left( \theta _{1{\mathbf {a}}}\right) )<0$, which in turn implies ${\mathbb {E}}_{\mu ^{*}}\left( \theta _{1 {\mathbf {a}}}\right) <\theta _{1{\mathbf {a}}}^{*}$. Conversely, suppose ${\mathbb {E}}_{\mu ^{*}}\left( \theta _{1{\mathbf {a}}}\right) <\theta _{1 {\mathbf {a}}}^{*}$. Since ${\mathbb {E}}_{\mu ^{*}}\left( \theta _{1 {\mathbf {a}}}\right) \le -{\hat{\beta }}^{*}$, by (27) it follows $a^{*}>0$. Moreover, being $\left( \theta _{1{\mathbf {a}}}^{*}- {\mathbb {E}}_{\mu ^{*}}\left( \theta _{1{\mathbf {a}}}\right) \right) /(\hat{ \beta }^{*}+{\mathbb {E}}_{\mu ^{*}}\left( \theta _{1{\mathbf {a}}}\right) )<0$, by (44) it holds $a^{*}>a^{o}$. (iii) Suppose $0<a^{*}<a^{o}$. By (44), $\left( \theta _{1{\mathbf {a}} }^{*}-{\mathbb {E}}_{\mu ^{*}}\left( \theta _{1{\mathbf {a}}}\right) \right) /({\hat{\beta }}^{*}+{\mathbb {E}}_{\mu ^{*}}\left( \theta _{1 {\mathbf {a}}}\right) )>0$, that is, ${\mathbb {E}}_{\mu ^{*}}\left( \theta _{1 {\mathbf {a}}}\right) \in (\theta _{1{\mathbf {a}}}^{*},-{\hat{\beta }}^{*})$ . Conversely, suppose ${\mathbb {E}}_{\mu ^{*}}\left( \theta _{1{\mathbf {a}} }\right) \in (\theta _{1{\mathbf {a}}}^{*},-{\hat{\beta }}^{*})$. By (27), $a^{*}>0$. Moreover, being $\left( \theta _{1\mathbf { a}}^{*}-{\mathbb {E}}_{\mu ^{*}}\left( \theta _{1{\mathbf {a}}}\right) \right) /({\hat{\beta }}^{*}+{\mathbb {E}}_{\mu ^{*}}\left( \theta _{1 {\mathbf {a}}}\right) )>0$, by (44) it holds $a^{*}<a^{o}$. $\square$

The loss function can be defined in terms of beliefs by setting $\ell \left( \mu ,\sigma \right) =\ell \left( B\left( \mu \right) ,\sigma \right)$. For instance, next we show that for the Phillips curve example it holds

45 $$\begin{aligned} \ell \left( \mu ^{*},\theta ^{*}\right) =\frac{\theta _{0}^{*2}\left( \theta _{1{\mathbf {a}}}^{*}-{\mathbb {E}}_{\mu ^{*}}\left( \theta _{1{\mathbf {a}}}\right) \right) ^{2}}{\left( 1+\left( {\hat{\beta }}^{*}+\theta _{1{\mathbf {a}}}^{*}\right) ^{2}\right) \left( 1+\left( \hat{\beta }^{*}+\theta _{1{\mathbf {a}}}^{*}\right) \left( {\hat{\beta }}^{*}+ {\mathbb {E}}_{\mu ^{*}}\left( \theta _{1{\mathbf {a}}}\right) \right) \right) ^{2}}. \end{aligned}$$

There is a zero welfare loss if and only if ${\mathbb {E}}_{\mu ^{*}}\left( \theta _{1{\mathbf {a}}}\right) =\theta _{1{\mathbf {a}}}^{*}$, that is, if and only if the monetary authority’s expected value of the coefficient $\theta _{1{\mathbf {a}}}$ is correct. Otherwise, the loss is nonzero, as (45) shows.

#### Proof of Proposition 11 and eq. (45)

First note that

$$\begin{aligned} R\left( a^{o},\theta ^{*}\right)= & {} -\theta _{0}^{*2}-\left( \hat{\beta }^{*}+\theta _{1{\mathbf {a}}}^{*}\right) ^{2}a^{o2}-\left( {\hat{\beta }} ^{*}\theta _{3}^{*}\right) ^{2}-\theta _{2}^{*2}\\&-2\theta _{0}^{*}\left( {\hat{\beta }}^{*}+\theta _{1{\mathbf {a}}}^{*}\right) a^{o}-a^{o2}-\theta _{3}^{*2}, \end{aligned}$$

and

$$\begin{aligned} R\left( a^{*},\theta ^{*}\right)= & {} -\theta _{0}^{*2}-\left( \hat{ \beta }^{*}+\theta _{1{\mathbf {a}}}^{*}\right) ^{2}\left( a^{*}\right) ^{2}-\left( {\hat{\beta }}^{*}\theta _{3}^{*}\right) ^{2}-\theta _{2}^{*2}\\&-2\theta _{0}^{*}\left( {\hat{\beta }}^{*}+\theta _{1{\mathbf {a}}}^{*}\right) a^{*}-\left( a^{*}\right) ^{2}-\theta _{3}^{*2}. \end{aligned}$$

Hence,

$$\begin{aligned} \ell \left( a^{*},\theta ^{*}\right)&=\max _{a\in A}R\left( a,\theta ^{*}\right) -R\left( a^{*},\theta ^{*}\right) =R\left( a^{o},\theta ^{*}\right) -R\left( a^{*},\theta ^{*}\right) \\&=-\left( {\hat{\beta }}^{*}+\theta _{1{\mathbf {a}}}^{*}\right) ^{2}\left( a^{o2}-a^{*2}\right) -2\theta _{0}^{*}\left( {\hat{\beta }} ^{*}+\theta _{1{\mathbf {a}}}^{*}\right) \left( a^{o}-a^{*}\right) -\left( a^{o2}-a^{*2}\right) . \end{aligned}$$

Suppose $a^{o}=0$, that is, $\theta _{0}^{*}\left( {\hat{\beta }}^{*}+\theta _{1{\mathbf {a}}}^{*}\right) =0$. Then

$$\begin{aligned} \ell \left( a^{*},\theta ^{*}\right)= & {} \left( 1+\left( {\hat{\beta }} ^{*}+\theta _{1{\mathbf {a}}}^{*}\right) ^{2}\right) a^{*2}\\= & {} -\left( 1+\left( {\hat{\beta }}^{*}+\theta _{1{\mathbf {a}}}^{*}\right) ^{2}\right) \frac{\theta _{0}^{*2}\left( {\hat{\beta }}^{*}+{\mathbb {E}} _{\mu ^{*}}\left( \theta _{1{\mathbf {a}}}\right) \right) ^{2}}{\left( 1+\left( {\hat{\beta }}^{*}+\theta _{1{\mathbf {a}}}^{*}\right) \left( {\hat{\beta }}^{*}+{\mathbb {E}}_{\mu ^{*}}\left( \theta _{1{\mathbf {a}} }\right) \right) \right) ^{2}}. \end{aligned}$$

If $\theta _{0}^{*}\ne 0$, then ${\hat{\beta }}^{*}+\theta _{1\mathbf {a }}^{*}=0,$ thus,

46 $$\begin{aligned} \ell \left( a^{*},\theta ^{*}\right) =\theta _{0}^{*2}\left( {\hat{\beta }}^{*}+{\mathbb {E}}_{\mu ^{*}}\left( \theta _{1{\mathbf {a}} }\right) \right) ^{2}=\theta _{0}^{*2}\left( {\mathbb {E}}_{\mu ^{*}}\left( \theta _{1{\mathbf {a}}}\right) -\theta _{1{\mathbf {a}}}^{*}\right) ^{2}. \end{aligned}$$

If ${\hat{\beta }}^{*}+\theta _{1{\mathbf {a}}}^{*}\ne 0$, then $\theta _{0}^{*}=0,$ thus,

47 $$\begin{aligned} \ell \left( a^{*},\theta ^{*}\right) =0. \end{aligned}$$

Next suppose $a^{o}\ne 0$. It holds $-2a^{o}\left( 1+\left( {\hat{\beta }} ^{*}+\theta _{1{\mathbf {a}}}^{*}\right) ^{2}\right) =2\theta _{0}^{*}\left( {\hat{\beta }}^{*}+\theta _{1{\mathbf {a}}}^{*}\right)$ , thus, $1+\left( {\hat{\beta }}^{*}+\theta _{1{\mathbf {a}}}^{*}\right) ^{2}=-\theta _{0}^{*}\left( {\hat{\beta }}^{*}+\theta _{1{\mathbf {a}} }^{*}\right) /a^{o}$. Hence,

$$\begin{aligned} \ell \left( a^{*},\theta ^{*}\right)&=-\left( {\hat{\beta }}^{*}+\theta _{1{\mathbf {a}}}^{*}\right) ^{2}\left( a^{o2}-a^{*2}\right) -2\theta _{0}^{*}\left( {\hat{\beta }}^{*}+\theta _{1{\mathbf {a}}}^{*}\right) \left( a^{o}-a^{*}\right) -\left( a^{o2}-a^{*2}\right) \\&=-\left( a^{o}-a^{*}\right) \left[ \left( {\hat{\beta }}^{*}+\theta _{1{\mathbf {a}}}^{*}\right) ^{2}\left( a^{o}+a^{*}\right) +2\theta _{0}^{*}\left( {\hat{\beta }}^{*}+\theta _{1{\mathbf {a}}}^{*}\right) +a^{o}+a^{*}\right] \\&=-\left( a^{o}-a^{*}\right) \left[ \left( {\hat{\beta }}^{*}+\theta _{1{\mathbf {a}}}^{*}\right) ^{2}\left( a^{o}+a^{*}\right) -2a^{o}\left( 1+\left( {\hat{\beta }}^{*}+\theta _{1{\mathbf {a}}}^{*}\right) ^{2}\right) +a^{o}+a^{*}\right] \\&=-\left( a^{o}-a^{*}\right) \left[ \left( {\hat{\beta }}^{*}+\theta _{1{\mathbf {a}}}^{*}\right) ^{2}\left( a^{*}-a^{o}\right) +a^{*}-a^{o}\right] \\&=-\left( a^{o}-a^{*}\right) \left[ \left( {\hat{\beta }} ^{*}+\theta _{1{\mathbf {a}}}^{*}\right) ^{2}+1\right] \left( a^{*}-a^{o}\right) \\&=\left( a^{o}-a^{*}\right) ^{2}\left[ \left( {\hat{\beta }}^{*}+\theta _{1{\mathbf {a}}}^{*}\right) ^{2}+1\right] =\left( a^{*}-a^{o}\right) ^{2}\left[ \left( {\hat{\beta }}^{*}+\theta _{1{\mathbf {a}} }^{*}\right) ^{2}+1\right] \\&=-\left( a^{*}-a^{o}\right) ^{2}\frac{\theta _{0}^{*}\left( \hat{ \beta }^{*}+\theta _{1{\mathbf {a}}}^{*}\right) }{a^{o}}=-\theta _{0}^{*}\left( {\hat{\beta }}^{*}+\theta _{1{\mathbf {a}}}^{*}\right) \frac{\left( a^{*}-a^{o}\right) ^{2}}{a^{o}} \\&=\left( 1+\left( {\hat{\beta }}^{*}+\theta _{1{\mathbf {a}}}^{*}\right) ^{2}\right) \left( a^{*}+\frac{\theta _{0}^{*}\left( {\hat{\beta }} ^{*}+\theta _{1{\mathbf {a}}}^{*}\right) }{1+\left( {\hat{\beta }}^{*}+\theta _{1{\mathbf {a}}}^{*}\right) ^{2}}\right) ^{2}. \end{aligned}$$

In the previous proof we showed that

$$\begin{aligned} a^{*}-a^{o}=\frac{\theta _{0}^{*}\left( \theta _{1{\mathbf {a}}}^{*}-{\mathbb {E}}_{\mu ^{*}}\left( \theta _{1{\mathbf {a}}}\right) \right) }{ \left( 1+\left( {\hat{\beta }}^{*}+\theta _{1{\mathbf {a}}}^{*}\right) ^{2}\right) \left( 1+\left( {\hat{\beta }}^{*}+\theta _{1{\mathbf {a}}}^{*}\right) \left( {\hat{\beta }}^{*}+{\mathbb {E}}_{\mu ^{*}}\left( \theta _{1{\mathbf {a}}}\right) \right) \right) }. \end{aligned}$$

Hence,

$$\begin{aligned} \ell \left( \mu ^{*},\theta ^{*}\right)&=-\theta _{0}^{*}\left( {\hat{\beta }}^{*}+\theta _{1{\mathbf {a}}}^{*}\right) \frac{ \left( a^{*}-a^{o}\right) ^{2}}{a^{o}} \\&=\theta _{0}^{*}\left( {\hat{\beta }}^{*}+\theta _{1{\mathbf {a}}}^{*}\right) \frac{\theta _{0}^{*2}\left( \theta _{1{\mathbf {a}}}^{*}- {\mathbb {E}}_{\mu ^{*}}\left( \theta _{1{\mathbf {a}}}\right) \right) ^{2}}{ \left( 1+\left( {\hat{\beta }}^{*}+\theta _{1{\mathbf {a}}}^{*}\right) ^{2}\right) ^{2}\left( 1+\left( {\hat{\beta }}^{*}+\theta _{1{\mathbf {a}} }^{*}\right) \left( {\hat{\beta }}^{*}+{\mathbb {E}}_{\mu ^{*}}\left( \theta _{1{\mathbf {a}}}\right) \right) \right) ^{2}}\\&\quad \frac{1+\left( {\hat{\beta }} ^{*}+\theta _{1{\mathbf {a}}}^{*}\right) ^{2}}{\theta _{0}^{*}\left( {\hat{\beta }}^{*}+\theta _{1{\mathbf {a}}}^{*}\right) } \\&=\frac{\theta _{0}^{*2}\left( \theta _{1{\mathbf {a}}}^{*}-{\mathbb {E}} _{\mu ^{*}}\left( \theta _{1{\mathbf {a}}}\right) \right) ^{2}}{\left( 1+\left( {\hat{\beta }}^{*}+\theta _{1{\mathbf {a}}}^{*}\right) ^{2}\right) \left( 1+\left( {\hat{\beta }}^{*}+\theta _{1{\mathbf {a}}}^{*}\right) \left( {\hat{\beta }}^{*}+{\mathbb {E}}_{\mu ^{*}}\left( \theta _{1{\mathbf {a}}}\right) \right) \right) ^{2}}. \end{aligned}$$

It is easy to check that, along with (46) and (47), this completes the proof. $\square$

#### Proof of eq. (40)

It holds

$$\begin{aligned} \ell \left( a^{*},\theta ^{*}\right)= & {} \left( 1+\left( {\hat{\beta }} ^{*}+\theta _{1{\mathbf {a}}}^{*}\right) ^{2}\right) \left( -\frac{ \theta _{0}^{*}{\hat{\beta }}^{*}\mu _{k}^{*}}{1+{\hat{\beta }}^{*}\mu _{k}^{*}\left( {\hat{\beta }}^{*}+\theta _{1{\mathbf {a}}}^{*}\right) }+\frac{\theta _{0}^{*}\left( {\hat{\beta }}^{*}+\theta _{1 {\mathbf {a}}}^{*}\right) }{1+\left( {\hat{\beta }}^{*}+\theta _{1\mathbf {a}}^{*}\right) ^{2}}\right) ^{2} \\= & {} \left( 1+\left( {\hat{\beta }}^{*}+\theta _{1{\mathbf {a}}}^{*}\right) ^{2}\right) \left( \frac{-\theta _{0}^{*}{\hat{\beta }}^{*}\mu _{k}^{*}\left( 1+\left( {\hat{\beta }}^{*}+\theta _{1{\mathbf {a}}}^{*}\right) ^{2}\right) +\theta _{0}^{*}\left( {\hat{\beta }}^{*}+\theta _{1{\mathbf {a}}}^{*}\right) \left( 1+{\hat{\beta }}^{*}\mu _{k}^{*}\left( {\hat{\beta }}^{*}+\theta _{1{\mathbf {a}}}^{*}\right) \right) }{ \left( 1+{\hat{\beta }}^{*}\mu _{k}^{*}\left( {\hat{\beta }}^{*}+\theta _{1{\mathbf {a}}}^{*}\right) \right) \left( 1+\left( {\hat{\beta }} ^{*}+\theta _{1{\mathbf {a}}}^{*}\right) ^{2}\right) }\right) ^{2} \\= & {} \frac{\theta _{0}^{*2}\left( {\hat{\beta }}^{*}\mu _{k}^{*}+\hat{ \beta }^{*}+\theta _{1{\mathbf {a}}}^{*}\right) ^{2}}{\left( 1+\hat{\beta }^{*}\mu _{k}^{*}\left( {\hat{\beta }}^{*}+\theta _{1{\mathbf {a}} }^{*}\right) \right) ^{2}\left( 1+\left( {\hat{\beta }}^{*}+\theta _{1 {\mathbf {a}}}^{*}\right) ^{2}\right) }, \end{aligned}$$

as desired. $$\square$$

## Footnotes

1 See, e.g., Easley and Kiefer (1988) and the references therein. We use boldface letters to distinguish random variables from their realizations. The working paper version of Battigalli et al. (2019) describes the exact relationship between our framework and the stochastic control framework of Easley and Kiefer, showing that they are essentially equivalent.

2 That is, to models that induce the same distribution of outcomes as $\sigma ^{*}$ when $a^{*}$ is chosen.

3 The one-person assumption matters only for two related results of our abstract analysis. We expand on this in the literature review.

4 One can easily prove also a kind of converse result: for every self-confirming equilibrium pair $\left( a^{*},\mu ^{*}\right)$ there are a prior belief, a discount factor, and a subjectively optimal strategy such that the resulting action process converges to $a^{*}$ almost surely. However, it may be necessary to allow for knife-edge cases, e.g., when $a^{*}$ is weakly dominated.

5 In order to remove pervasive inconsistencies of pre-rational-expectations models, rational-expectations models often assume that decision makers know the true data generating process, thus making decisions objectively optimal. The traditional Nash equilibrium concept shares this objective best-reply feature. The Bayesian Nash equilibrium concept of Harsanyi (1967) instead allows for subjective and incorrect beliefs about parameters, without imposing a confirmed-beliefs condition. Therefore, Bayesian Nash equilibrium does not refine self-confirming equilibrium.

6 Some further topics are analyzed in the working paper version.

7 See also, Cogley and Sargent (2005), Sargent et al. (2006), and Cogley et al. (2007).

8 The phrase “escaping Nash inflation” in the title of Cho et al. (2002) deserves an explanation. When the decision model is interpreted as a game between the monetary authority and a representative agent, a self-confirming equilibrium outcome is also a (possibly subgame imperfect) Nash equilibrium outcome. Battigalli (1987) and Fudenberg and Levine (1993a) provide sufficient conditions for the realization-equivalence between Nash and self-confirming equilibrium. Such conditions are satisfied in the model of Cho et al.

9 In his work on rational belief equilibria, Kurz (1994a, 1994b) analyzes stochastic dynamics where agents’ beliefs may be incorrect, but are eventually consistent with the long-run frequencies of observables, which is in the spirit of self-confirming equilibrium. The most important difference with the literature on the latter is that, although Kurz analyzes multi-agent systems, he does not use a game theoretic framework. Specifically, unlike game models, there is no function specifying how agents’ actions and—possibly—exogenous variables determine outcomes and observables.

10 The model is not explicitly represented as a game. Therefore the connection to the traditional self-confirming equilibrium concept is not immediate.

11 See Kechris (2012) for the properties of standard Borel spaces that we use.

12 That is, the sigma algebra generated by the evaluation maps $\xi \mapsto \xi \left( B\right)$ for all $B\in {\mathcal {X}}$.

13 The usual notation for the Dirac measure concentrated on *x* is $\delta _{x}.$

14 See Appendix A.1. In the applied probability literature, $\hat{ \varphi }(\xi )\left( B\right)$ is sometimes denoted by ${\hat{\varphi }}\left( B\mid \xi \right)$, interpreted as the probability of observing a realization in *B* given $\xi$ with “measurement” $\varphi$.

15 Model uncertainty is also called model ambiguity (see Hansen & Marinacci, 2016).

16 If, instead, the process of states were assumed to be Markovian, probability models would be kernel functions (with finitely many states, transition probability matrices) rather than elements of $\Delta \left( S\right)$.

17 The integral is well defined, for each $\Sigma$ and each $\mu \in \Delta \left( \Sigma \right)$, because the expected payoff function $R\left( a,\cdot \right) :\Delta \left( S\right) \rightarrow \left[ -\infty ,\infty \right)$ is, for every action *a*, measurable and bounded above on $\Delta \left( S\right)$ and hence on $\Sigma$.

18 See Marinacci (2015) for a discussion of this setup. Classical SEU is proposed by Cerreia-Vioglio et al. (2013), where “classical” refers to the fact that a posited set of probability models is a basic feature of classical statistics.

19 See Corollary 4 in Appendix A.1.

20 We write random variables in boldface font and their realization in normal font.

21 Ignoring the 0-price floor, $\left( {\bar{\theta }}-c\right) /2$ is the best reply to the most optimistic belief. The condition guarantees that, at this largest output, price is strictly positive even with the lowest inverse demand function. This implies that the standard first-order conditions identify a global subjective optimum.

22 Recall that $\Sigma \cong \left[ {\underline{\theta }},{\bar{\theta }}\right]$ means that $\Sigma$ and $\left[ {\underline{\theta }},{\bar{\theta }}\right]$ are isomorphic.

23 Here $f_{a}:S\rightarrow M$ denotes the section $f\left( a,\cdot \right)$ of *f* at *a*.

24 As previously observed, this is sometimes denoted ${\hat{f}}_{a}\left( B\mid \sigma \right)$.

25 We can also write ${\hat{\Sigma }}_{a}\left( \sigma \right) =\{ \sigma ^{\prime }\in \Sigma :\sigma _{|{\mathcal {F}}_{a}}^{\prime }=\sigma _{|{\mathcal {F}} _{a}}\}$, i.e., partial identification is determined by the information sigma algebra ${\mathcal {F}}_{a}$.

26 Here, since ${\hat{\Sigma }}_{a^{*}}\left( \sigma ^{*}\right)$ is a measurable subset of $\Sigma$, the set $\Delta ({\hat{\Sigma }}_{a^{*}}\left( \sigma ^{*}\right) )$ is identified with the family of elements of $\Delta \left( \Sigma \right)$ that assign probability 1 to $\hat{ \Sigma }_{a^{*}}\left( \sigma ^{*}\right)$.

27 See the example in the introduction.

28 The conjectural equilibrium conditions, stated for games by Battigalli (1987), are written in predictive form.

29 This lemma extends Lemma 1 of Battigalli et al. (2015) to the present setup with possibly infinite spaces of actions and states.

30 On the other hand, without noise (${\bar{\varepsilon }}=0$) a one-off experimentation with positive output would identify the true model, making the no-experimentation region in the belief-discount factor space very small.

31 Section  6 of the working paper version contains a more general analysis of self-confirming economic policies.

32 The economic interpretation is that planned inflation *a* affects agents’ expectations to an extent parameterized by $\theta _{1{\mathbf {a}}}$.

33 In the general case, for any measurable set of outcomes

$$O\subseteq U\times \Pi, \begin{aligned} {\hat{f}}_{a}\left( \sigma \right) \left( O\right) =\left( \sigma \circ f_{a}^{-1}\right) \left( O\right) =\sigma \left( \left\{ \left( w,\varepsilon ,\theta \right) :\left( {\varvec{u}}\left( a,w,\varepsilon ,\theta \right) ,\varvec{\pi }\left( a,w,\varepsilon ,\theta \right) \right) \in O\right\} \right). \end{aligned}$$

34 The map $\theta \mapsto q\times \delta _{\theta }$ is bijective and measurable. See Corollary 3 in the appendix.

35 Whenever convenient, in what follows we will use the shorthand notation ${\mathbb {E}}$ for integrals, for example ${\mathbb {E}}_{q}\left( \varepsilon \right) =\int _{W\times E}\varepsilon dq\left( w,\varepsilon \right)$.

36 Formally, ${\varvec{u}}_{a}$ and $\varvec{\pi }_{a}$ are the sections ${\varvec{u}}\left( a,\cdot \right)$ and $\varvec{\pi }\left( a,\cdot \right)$ at policy *a* of the random variables ${\varvec{u}}$ and $\varvec{\pi }$, respectively.

37 The Phillips regression $u=\alpha +\beta \pi$ is run by the monetary authority using long run data.

38 By Assumption 2, the beta coefficient of the Phillips regression is negative, that is, ${\hat{\beta }}<0$. This negative sign will be tacitly assumed when interpreting our findings.

39 Recall that ${\hat{\beta }}^{*}$ is the beta regression coefficient of unemployment over inflation (given the true model $\theta ^{*}$).

40 Note that, with the dogmatic value ${\bar{\theta }}_{1{\mathbf {a}}}$ of $\theta _{1{\mathbf {a}}}$ in place of its expectation ${\mathbb {E}}_{\mu ^{*}}\left( \theta _{1{\mathbf {a}}}\right)$, the dogmatic equilibrium relations are identical to the general ones. This is a consequence of the certainty equivalence principle stated in Proposition 3.

41 Both $\xi _{2}^{*}$ and ${\mathbb {E}}_{\mu ^{*}}(\xi _{2})$ are negative (Assumption 2), and so ${\mathbb {E}}_{\mu ^{*}}(\xi _{2})\gtrless \xi _{2}^{*}$ if and only if $\left| {\mathbb {E}}_{\mu ^{*}}(\xi _{2})\right| \lessgtr \left| \xi _{2}^{*}\right|$.

42 Since $\xi _{2}^{*}\le 0$ (Assumption 2), the cases considered in the proposition exhaust all possibilities. Also note that, since ${\mathbb {E}}_{\mu ^{*}}\left( \theta _{1a}\right) \le -{\hat{\beta }}^{*}$, ${\mathbb {E}}_{\mu ^{*}}\left( \theta _{1a}\right) =-{\hat{\beta }}^{*}$ is equivalent to $\mu ^{*}(\theta _{1a}=-{\hat{\beta }}^{*})=1$.

43 Cogley and Sargent (2005) and Cogley et al. (2007) study dynamic Bayesian policy problems where beliefs assign positive probability to three model economies (dynamic specifications of the two models we consider here and a third related model).

44 That is, lack of certainty about the objective probabilities of consequences of choices.
